# Novel Trifluoromethylcoumarinyl Urea Derivatives: Synthesis, Characterization, Fluorescence, and Bioactivity

**DOI:** 10.3390/molecules23030600

**Published:** 2018-03-07

**Authors:** Lili Qiao, Shuanghong Hao

**Affiliations:** Research Center of Agro-bionic Engineering & Tech. of Shandong Province, College of Chemistry & Pharm., Qingdao Agricultural University, Qingdao 266109, China; qiaolili07@163.com

**Keywords:** trifluoromethylcoumarinyl urea, synthesis, fluorescence, herbicidal activity, antifungal activity

## Abstract

A series of novel trifluoromethylcoumarinyl urea derivatives were designed, synthesized, and characterized by ^1^H-NMR, ^13^C-NMR, and HR-ESI-MS. The fluorescence spectra of the target compounds were recorded. The spectra show that most of the title compounds glow green with λ_max_^em^ of 500–517 nm, while compounds **5r**, **5s**, **5u**, and **5l** (compounds named by authors) glow violet with λ_max_^em^ of 381–443 nm. Moreover, the herbicidal and antifungal activities of the synthesized compounds were evaluated for their potential use as pesticides. The results indicate that compound **5f** against the caulis of *Amaranthus* *retroflexus* and compounds **5j** and **5l** against the taproot of *Digitaria* *sanguinalis* are equivalent to the commercial herbicide Acetochlor. Nine of the title compounds are more antifungal than commercial fungicide Carbendazim against *Botrytis cinerea*.

## 1. Introduction

Ureas are very important compounds in organic chemistry because of their extensive applications in medicine and agriculture. The substituted ureas exhibit diversified medicinal activities such as antimicrobial [1], antiviral [2], anti-HIV [3], anti-AIDS [4], anti-malaria [5], anti-proliferative [6], antitumor [7], anticancer [8], anti-inflammatory [9], anti-ulcerogenic [10], anticonvulsant [11], anti-nociceptive [12], antihypertensive [9], antidepressant [13], complement inhibition [14], HDL-elevating [15], and inhibition of nitric oxide [16]. Urea compounds also possess a broad spectrum of pesticidal activities, such as benzoylurea type insecticides (Diflubenzuron, Chlorbenzuron, etc.) [17], fungicides (Triclocarban, Pencycuron, etc.) [18], sulfonylurea type herbicides (Chlorsulfuron, Nicosulfuron, etc.), and urea herbicides (Benzthiazuron, Siduron, etc.) [19], as well as plant growth regulators (Thidiazuron, Forchlorfenuron, etc.) [20].

On the other hand, coumarin is a subunit of many natural products and a wide range of synthetic compounds, constituting an important class of heterocycles. These kinds of compounds are widely used in the food, cosmetic, and perfume industries. Coumarins are also an important class of fluorescents and are used as laser dyes, brighteners, fluorescent labels, and other organic optical materials, such as emission layers of organic light-emitting diodes (OLED) [21,22,23,24]. Additionally, they are sometimes used as probes to monitor certain physiological process and trace harmful substances in environment [25,26,27]. It has been noted that the photophysical and spectroscopic properties of coumarin can be easily adjusted by the introduction of different substituents into the core, which leads them to be more flexible and fit well in various applications [28]. It is reported that the electron-withdrawing groups on 3- or 4-position and electron-releasing groups on 6- or 7-position of the coumarin skeleton could enhance the band intensity of fluorescence [29]. Moreover, coumarins have been extensively studied for their use as biological agents with anti-depressant, anticoagulant, anti-inflammatory, antibacterial, antifungal, antimalarial, moluscicidal, and anthelmintic activities [30,31,32,33,34,35,36]. Much research has been focused on coumarin derivatives for their pesticidal activities too. One of the representative compounds is 7-hydroxyl-4-methylcoumarin, which exhibited interesting pesticidal activities of allelopathy and antifungal, and it is reported that the C7 hydroxyl group and C4 methyl substitution contributed significantly to the activity [37,38]. Moreover, brominated derivatives of the compound showed remarkable larvicidal and ovicidal activities against vector mosquitoes [39]. Derivatives of 7-hydroxyl-4-methylcoumarin are also reported to show anthelmintic and acaricidal activities [40,41].

In addition, fluorine is the most electronegative of all elements, which endows it with unique properties. Indeed, it is commonly accepted that introducing a fluorine-containing group onto the molecule scaffold often leads to improvement of the molecular chemical, physical, and biological properties [42]. To date, the trifluoromethyl (CF_3_) group has appeared as the archetypal and most sought-after fluorine-containing group, and there have been reported a huge and ever-increasing number of trifluoromethylated compounds in the literature [43,44]. The CF_3_ group is very important in medicinal chemistry [45] due to its high lipophilicity, easy transportation, and hard degradation in vivo [46,47]. Thus, the introduction of a CF_3_ group into bioactive molecules has become a preferable approach in pharmaceutical studies [48,49]. However, the literature about CF_3_-substituted coumarins is limited, and the reports about these compounds are mainly focused on their use as substrate probes to detect the physiology [50,51,52]. Also, in view of these observations and in continuation of our research program on the synthesis of fluorinated coumarins [53], we herein report the synthesis and characterization of some novel 4-trifluoromethyl-6-substituted-ureido-7-methoxylcoumarins, whose fluorescent properties and antifungal and herbicidal activities are also evaluated.

## 2. Results and Discussion

### 2.1. Chemistry

**Scheme 1:**
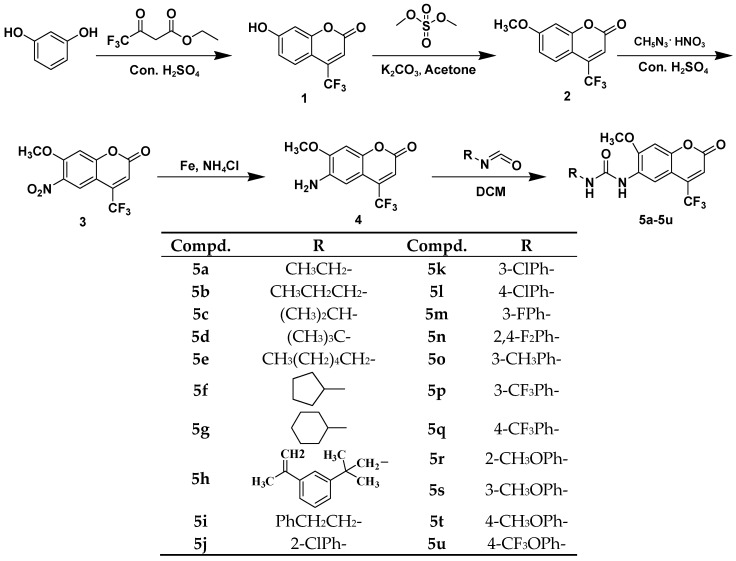
Synthetic route of title compounds.

The synthesis of target compounds was carried out according to Scheme 1. Compound **1** was prepared from the reaction of resorcinol with ethyl trifluoroacetoacetate catalyzed by Con. (concentrated) H_2_SO_4_. Compound **2** was synthesized by methylation of compound **1** with dimethyl sulfate using anhydrous K_2_CO_3_ as catalyst in acetone. Compound **3** was synthesized by nitration of compound **2** with guanidine nitrate in Con. H_2_SO_4_. Compound **4** was obtained by reduction of compound **3** with Fe powder in aqueous solution of NH_4_Cl. The target compounds **5a**–**5u** were furnished by addition of compounds **4** to a series of isocyanates catalyzed by triethylamine in CH_2_Cl_2_ (DCM). The structures of all target compounds were well characterized by ^1^H-NMR, ^13^C-NMR, and HR-ESI-MS. The spectrograms were listed in the supplementary materials.

### 2.2. UV Absorption and Fluorescence Characteristics

**Figure 1:**
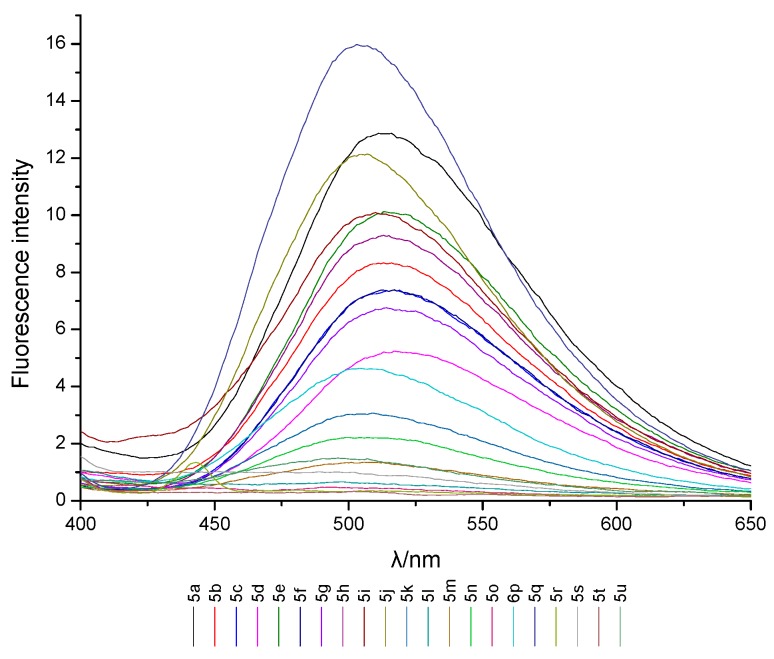
Fluorescence spectrum of title compounds.

The λ_max_^ab^ (maximum absorption wavelength), ε_max_ (molar absorptivity), λ_max_^em^ (maximum emission wavelength), Δλ (Stokes shift) and Φ_f_ (fluorescence quantum yield) of the title compounds are listed in Table 1. The λ_max_^ab^ of all the title compounds are 360–374 nm, and the ε_max_ are 5.24–7.67 × 10^3^ L·mol^−1^·cm^−1^. Most of the title compounds glow green fluorescence with λ_max_^em^ of 500–517 nm when illuminated by ultraviolet light, while compounds **5r**, **5s**, **5u**, and **5l** glow violet fluorescence with λ_max_^em^ of 381–443 nm (Figure 1). However compounds **5o** and **5t** do not fluoresce at the concentration. The values of Φ_f_ indicate that all the title compounds are weaker fluorescent compare to the standard reference compound quinine sulfate. Table 1 shows that alkyl substituted compounds **5a**–**5i** have higher fluorescence quantum yield than the phenyl substituted compounds **5k**–**5p** and **5r**–**5u**, but compounds **5j** and **5q** with high Φ_f_ were the exceptions. From the phenomenon, it can be concluded that electron-donating groups may favor the fluorescence of the compounds.

### 2.3. Herbicidal Activities

**Figure 2:**
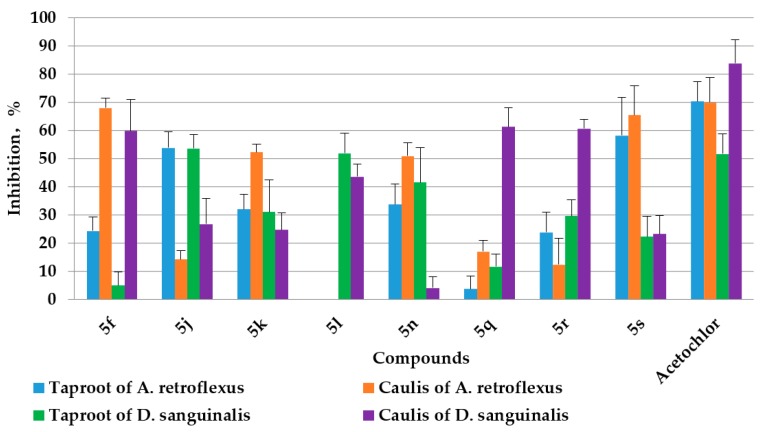
Herbicidal activities of title compounds.

The herbicidal activities of the 21 title compounds of 20 mg·L^−1^ and 100 mg·L^−1^ against the taproot and caulis growth of dicotyledonous weed *A. retroflexus* and monocotyledonous weed *D. sanguinalis* were assayed. The inhibition of the compounds with effectiveness of greater than 50% to at least one organ of the test weeds at 100 mg·L^−1^ are displayed in Figure 2. It indicates that compounds **5j** and **5s** show medium inhibition against the taproot growth of *A. retroflexus*, and compounds **5f**, **5s**, **5k** and **5n** show medium to high inhibition against the caulis growth of *A. retroflexus*. Moreover, compounds **5j** and **5l** show medium inhibition against the taproot growth of *D. sanguinalis*, and compounds **5f**, **5q**, and **5r** show medium inhibition against the caulis growth of *D. sanguinalis*. Furthermore, the herbicidal activities of **5f** against the caulis of *A. retroflexus*, **5j** and **5l** against the taproot of *D. sanguinalis* are equivalent to the commercial herbicide Acetochlor. It also shows that only compounds **5j** and **5s** are both active to the two test herbs; the other compounds display certain selectivity between the test herbs. Contrary to the fluorescence, the phenyl substituted compounds show higher herbicidal activity than the alkyl substituted compounds except compound **5f** substituted by cyclopentyl. All the compounds substituted by phenyls with chlorine (**5j**–**5l**) exhibit better activity. Compounds **5o** (3-CH_3_Ph-), **5r** (2-CH_3_OPh-) and **5s** (3-CH_3_OPh-) substituted by phenyls with electron donating groups are more active.

### 2.4. Antifungal Activities

**Figure 3:**
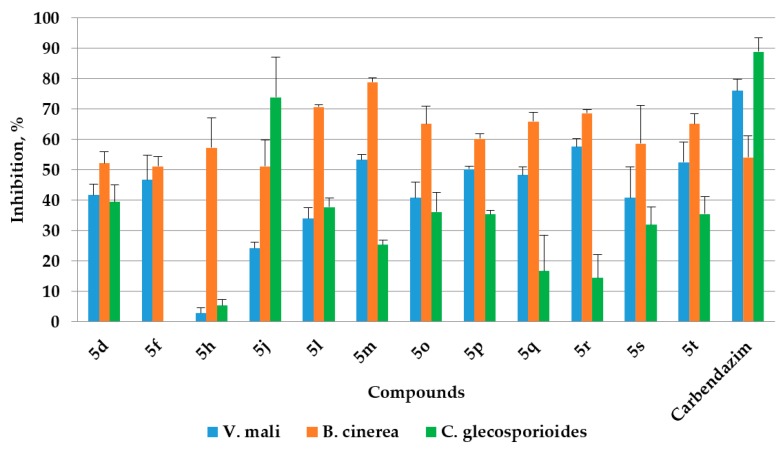
Antifungal activities of title compounds.

The in vitro antifungal activities of the title compounds of 20 mg·L^−1^ and 100 mg·L^−1^ against the mycelium growth of the phytopathogens *Valsa* *mali*, *B. cinerea*, *Colletotrichum glecosporioides*, and *Fusarium oxysporum* were assayed. The inhibition of the synthesized compounds with effectiveness of greater than 50% at 100 mg·L^−1^ are exhibited in Figure 3. This indicates that more compounds exhibit stronger inhibition against *B. cinerea* than the other three plant disease fungi (the inhibitory rates of all the title compounds against *F. oxysporum* are less than 50%, and they do not appear in Figure 3). There are only four title compounds showing medium inhibition against *V. mali*, and only one compound having an inhibitory rate of more than 50% to *C. glecosporioides*. Meanwhile, 12 of the title compounds exhibit medium to high activity against *B. cinerea*, and nine of them are more antifungal than the commercial fungicide Carbendazim. Based on the above description, it seems that the phenyl substitutes are of more benefit to the antifungal activity. Compounds **5j**, **5l**, and **5o**–**5t** with substitutes of –Cl, –CH_3_, –CF_3_, or –OCH_3_ on phenyls have more potential.

## 3. Experimental Section

### 3.1. Chemistry

#### 3.1.1. General Procedures

All chemicals were obtained from commercial sources and used without further purification. Glass plates pre-coated with silica gel 60 GF_254_ (Qingdao Haiyang Chemical Co., Ltd., Qingdao, China) were used for analytical thin layer chromatography (TLC). An uncorrected WRS-1B digital melting-point apparatus (Shanghai Precision Optical Instrument Co., Ltd., Shanghai, China) was used to determine the melting points. A Bruker Avance III HD 500 MHz nuclear magnetic resonance instrument (Bruker, Fällanden, Switzerland) was used to record the ^1^H- and ^13^C-NMR spectra in CDCl_3_ or DMSO-*d*_6_ (^1^H at 500 MHz and ^13^C at 126 MHz) using tetramethylsilane (TMS) as the internal standard. A maXis Q-TOF high-resolution mass spectra instrument (Bruker, Karlsruhe, Germany) was used to measure the HR-ESI-MS. An F-4600 fluorescence spectrophotometer (Hitachi, Tokyo, Japan) was used to record the fluorescence.

#### 3.1.2. Synthesis of 4-Trifluoromethyl-7-hydroxycoumarin (**1**)

Concentrated sulfuric acid (2 mL) and trifluoroacetoacetate (3.7 g, 10 mmol) were added dropwise in turn to the solution of resorcinol (2.2 g, 10 mmol) in ethanol (4 mL) with stirring. Then the mixture was heated to maintain at 80 °C until the end of the reaction was detected by TLC. The resulted thick magenta liquid was dripped into 10 mL of cold water with rigorous stirring. The precipitate was filtered and dried to give compound **1**. Yield 88.9%, m.p. (melting point) 175–176 °C [lit. (literature) 178–179 °C] [54].

#### 3.1.3. Synthesis of 4-Trifluoromethyl-7-methoxycoumarin (**2**)

Dimethyl sulfate (2.5 g, 20 mmol) was added dropwise to the suspension of compound **1** (5.8 g, 25 mmol) and anhydrous K_2_CO_3_ (4.1 g, 30 mmol) in 66 mL of acetone with stirring. Then the mixture was heated to reflux for 8 h. After K_2_CO_3_ was filtered out, the filtrate was rotary evaporated to 1/3 of the total volume. Then the residue was allowed to cool to room temperature until a white solid precipitated. The solid product was filtered out and washed with cold water to neutral pH to get compound **2**. Yield 97.8%, m.p. 110–112 °C (lit. 111–112 °C) [55].

#### 3.1.4. Synthesis of 4-Trifluoromethyl-6-nitro-7-methoxycoumarin (**3**)

Guanidine nitrate (1.4 g, 11.6 mmol) was added batch-wise to the solution of **2** (2.6 g, 10.7 mmol) in 16 mL Conc. H_2_SO_4_ with stirring at 0–5 °C. The mixture was stirred at room temperature for 3 h until the end of the reaction. Then the reaction mixture was poured into 40 mL of ice water with stirring to obtain a pale yellow solid. The solid product was filtered and dried to give compound **3**. Yield 97.7%, m.p. 118–119 °C (as reported) [53].

#### 3.1.5. Synthesis of 4-Trifluoromethyl-6-amino-7-methoxycoumarin (**4**)

The mixture of compound **3** (2.9 g, 10 mmol), iron powder (5.0 g, 90 mmol), NH_4_Cl (1.1 g, 20 mmol) and 50 mL water was mechanically stirred at room temperature until the end of the reaction. The mixture was extracted with ethyl acetate (50 mL × 3). The extract was dried over anhydrous sodium sulfate, then filtered and rotary evaporated to get a dark yellow crystal of compound **4**. Yield 70%, m.p. 179–180 °C (as reported) [53]. The compound is unstable and needs to be used as soon as possible for the next step.

#### 3.1.6. General Procedure for Synthesis of 4-Trifluoromethyl-6-amino-7-methoxycoumarinyl Urea Derivatives (**5a**–**5u**)

To a cool suspension of **4** (0.65 g, 2.5 mmol) in 3.5 mL of anhydrous dichloromethane, isocyanate (3 mmol) was added dropwise with stirring. The mixture was allowed to return to room temperature until the end of the reaction. The solid produced was filtered and chromatographed on a flash silica gel column (ethyl acetate/petroleum ether = 1:3) to give the target compounds **5a**–**5u**. The yield, appearance, m.p., NMR and HR-ESI-MS (MS) date of the synthesized compounds are listed below.

*1-Ethyl-3-(7-methoxy-2-oxo-4-(trifluoromethyl)-2H-chromen-6-yl) urea* (**5a**). Light yellow solid; yield: 42.8%; m.p. 232–233 °C; ^1^H-NMR (500 MHz, DMSO) δ: 8.68 (s, 1H), 8.14 (s, 1H), 7.23 (s, 1H), 6.95 (t, *J* = 5.3 Hz, 1H), 6.83 (s, 1H), 4.01 (s, 3H), 3.20–3.09 (m, 2H), 1.08 (t, *J* = 7.2 Hz, 3H); ^13^C-NMR (126 MHz, DMSO) δ: 159.46, 155.39, 152.17, 150.33, 140.41 (q, *J* = 31.5 Hz), 127.99, 122.27 (q, *J* = 275.94 Hz), 113.47 (q, *J* = 6.3 Hz), 111.52 (q, *J* = 1.26Hz), 106.07, 100.16, 57.24, 34.31, 15.72.; HR-ESI-MS *m*/*z*: 353.0712 [M + Na]^+^ (calculated for C_14_H_13_F_3_N_2_NaO_4_, 353.0725).

*1-Propyl-3-(7-methoxy-2-oxo-4-(trifluoromethyl)-2H-chromen-6-yl) urea* (**5b**). White solid; yield: 31.1%; m.p. 229 °C. ^1^H-NMR (500 MHz, DMSO) δ: 8.68 (s, 1H), 8.16 (s, 1H), 7.22 (s, 1H), 6.97 (t, *J* = 5.3 Hz, 1H), 6.82 (s, 1H), 4.00 (s, 3H), 3.06 (q, *J* = 5 Hz, 2H), 1.52–1.38 (m, 2H), 0.90 (t, *J* = 7.4 Hz, 3H); ^13^C-NMR (126MHz, DMSO) δ: 159.47, 155.50, 152.16, 150.32, 140.41 (q, *J* = 31.5 Hz), 128.03, 122.27 (q, *J* = 275.94 Hz), 113.49 (q, *J* = 6.3 Hz), 111.48, 106.09, 100.18, 57.26, 41.24, 23.31, 11.83; HR-ESI-MS *m*/*z*: 367.0882 [M + Na]^+^ (calculated for C_15_H_15_F_3_N_2_NaO_4_, 367.0882).

*1-Isopropyl-3-(7-methoxy-2-oxo-4-(trifluoromethyl)-2H-chromen-6-yl) urea* (**5c**). Light yellow solid; yield: 54.2%; m.p. 224 °C; ^1^H-NMR (500 MHz, DMSO) δ: 8.66 (s, 1H), 8.07 (s, 1H), 7.20 (s, 1H), 6.90 (d, *J* = 7.2 Hz, 1H), 6.80 (s, 1H), 3.99 (s, 3H), 3.82–3.72 (m, 1H), 1.10 (d, *J* = 6.4 Hz, 6H); ^13^C-NMR (126 MHz, DMSO) δ: 159.45, 154.74, 152.11, 150.28, 140.42 (q, *J* = 31.5 Hz), 128.03, 122.16 (q, *J* = 275.94 Hz), 113.43 (q, *J* = 5.0 Hz), 111.36 (q, *J* = 2.52 Hz), 106.06, 100.11, 57.22, 41.36, 23.41; ESI-MS *m*/*z*: 345.30 [M + Na]^+^ (calculated for C_15_H_16_F_3_N_2_O_4_, 345.21).

*1-(tert-Butyl)-3-(7-methoxy-2-oxo-4-(trifluoromethyl)-2H-chromen-6-yl) urea* (**5d**). yellow solid; Yield: 31.1%; m.p. 237 °C; ^1^H-NMR (500 MHz, CDCl_3_) δ: 8.54 (s, 1H), 6.87 (s, 1H), 6.83 (s, 1H), 6.64 (s, 1H), 4.90 (s, 1H), 3.93 (s, 3H), 1.40 (s, 9H); ^13^C-NMR (126 MHz, CDCl_3_) δ: 159.76 (q, *J* = 6.2 Hz), 152.2 (q, *J* = 22.68 Hz), 151.92 (q, *J* = 5.5 Hz), 150.76 (q, *J* = 7.3 Hz), 142.11 (q, *J* = 32.76 Hz), 127.15 (q, *J* = 12.2 Hz), 121.57 (q, *J* = 275.94 Hz), 113.46 (q, *J* = 2.52 Hz), 112.58 (q, *J* =5.04 Hz), 106.85, 98.98 (q, *J* = 6.2 Hz), 56.33, 51.03, 29.31; HR-ESI-MS *m*/*z*: 381.1038 [M + Na]^+^ (calculated for C_16_H_17_F_3_N_2_NaO_4_, 381.1038).

*1-Hexyl-3-(7-methoxy-2-oxo-4-(trifluoromethyl)-2H-chromen-6-yl) urea* (**5e**). Light yellow solid; yield: 31.2%; m.p. 143 °C; ^1^H-NMR (500 MHz, CDCl_3_) δ: 8.68 (s, 1H), 7.29 (s, 1H), 6.82 (s, 1H), 6.63 (s, 1H), 5.47 (t, *J* = 5.0 Hz, 1H), 3.89 (s, 3H), 3.28 (q, *J* = 5 Hz, 2H), 1.58–1.48 (m, 2H), 1.38–1.20 (m, 6H), 0.85 (t, *J* = 6.6 Hz, 3H); ^13^C-NMR (126 MHz, CDCl_3_) δ: 159.73, 154.77, 151.80, 150.85, 142.13 (q, *J* = 32.76 Hz), 126.90, 122.63 (q, *J* = 122.37 Hz), 113.61 (q, *J* = 1.26 Hz), 112.66 (q, *J* = 5.04 Hz), 106.90, 98.98, 56.38, 40.53, 31.50, 29.99, 26.56, 22.57, 14.00; HR-ESI-MS *m*/*z*: 409.1346 [M + Na]^+^ (calculated for C_18_H_21_F_3_N_2_NaO_4_, 409.1351).

*1-Cyclopentyl-3-(7-methoxy-2-oxo-4-(trifluoromethyl)-2H-chromen-6-yl) urea* (**5f**). Light yellow solid; yield: 43.6%; m.p. 225 °C; ^1^H-NMR (500 MHz, DMSO) δ: 8.68 (s, 1H), 8.07 (s, 1H), 7.21 (s, 1H), 7.01 (d, *J* = 6.9 Hz, 1H), 6.81 (s, 1H), 4.00 (s, 3H), 3.97-3.91 (m, 1H), 1.90–1.81 (m, 2H), 1.72–1.48 (m, 4H), 1.45–1.31 (m, 2H); ^13^C-NMR (126 MHz, DMSO) δ: 159.49, 155.02, 152.10, 150.30, 140.42 (q, *J* = 32.76 Hz), 128.04, 122.29(q, *J* = 273.42 Hz),113.52 (q, *J* = 1.26 Hz), 111.34 (q, *J* = 2.52 Hz), 106.10, 100.18, 57.27, 51.28, 33.30, 23.59; HR-ESI-MS *m*/*z*: 393.1033 [M + Na]^+^ (calculated for C_17_H_17_F_3_N_2_NaO_4_, 393.1038).

*1-Cyclohexyl-3-(7-methoxy-2-oxo-4-(trifluoromethyl)-2H-chromen-6-yl) urea* (**5g**). Light yellow solid; yield 48.9%; m.p. 265 °C; ^1^H-NMR (500 MHz, DMSO) δ: 8.68 (s, 1H), 8.13 (s, 1H), 7.23 (s, 1H), 6.95 (d, *J* = 7.5 Hz, 1H), 6.82 (s, 1H), 3.99 (s, 3H), 3.54–3.45 (m, 1H),1.86–1.76 (m, 2H), 1.72–1.63 (m, 2H), 1.37–1.11 (m, 6H); ^13^C-NMR (126 MHz, DMSO) δ: 159.48, 154.70, 152.14, 150.29, 140.42 (q, *J* = 31.5 Hz), 128.06, 122.28(q, *J* = 275.94 Hz), 113.49(q, *J* = 6.3 Hz), 111.40 (q, *J* = 2.52 Hz), 106.09, 100.18, 57.27, 48.02, 33.35, 25.70, 24.71; HR-ESI-MS *m*/*z*: 407.1191 [M + Na]^+^ (calculated for C_18_H_19_F_3_N_2_NaO_4_, 407.1195).

*1-(2-(3-(Prop-1-en-2-yl)phenyl)propan-2-yl)-3-(7-methoxy-2-oxo-4-(trifluoromethyl)-2H-chromen-6-yl) urea* (**5h**). Light yellow solid; yield: 20.0%; m.p. 228–229 °C; ^1^H-NMR (500 MHz, DMSO) δ: 8.52 (s, 1H), 8.29 (s, 1H), 7.50 (s, 1H), 7.47 (s, 1H), 7.39–7.29 (m, 3H), 7.22 (s, 1H), 6.79 (s, 1H), 5.38 (s, 1H), 5.08 (s, 1H), 4.01 (s, 3H), 2.11 (s, 3H), 1.62 (s, 6H); ^13^C-NMR (126 MHz, DMSO) δ: 159.43, 154.39, 152.20, 150.33, 148.72, 143.45, 140.62, 140.34 (q, *J* = 31.5 Hz), 128.54, 128.04, 124.66, 123.56, 122.22 (q, *J* = 275.94 Hz), 122.21, 113.47 (q, *J* = 6.3 Hz), 112.89, 111.22, 106.03, 100.21, 57.29, 55.01, 30.25, 22.01; HR-ESI-MS *m*/*z*: 483.1504 [M + Na]^+^ (calculated for C_24_H_23_F_3_N_2_NaO_4_, 483.1508).

*1-Phenethyl-3-(7-methoxy-2-oxo-4-(trifluoromethyl)-2H-chromen-6-yl) urea* (**5i**). Light yellow solid; yield: 41.7%; m.p. 222 °C; ^1^H-NMR (500 MHz, DMSO) δ: 8.68 (s, 1H), 8.22 (s, 1H), 7.36–7.29 (m, 2H), 7.27–7.24 (m, 2H), 7.23–7.18 (m, 2H), 6.99 (t, *J* = 5.1 Hz, 1H), 6.82 (s, 1H), 3.98 (s, 3H), 3.38 (q, *J* = 6.6 Hz, 2H), 2.75 (t, *J* = 7.0 Hz, 2H); ^13^C-NMR (126 MHz, DMSO) δ: 159.46, 155.48, 152.21, 150.38, 140.40 (q, *J* = 31.5 Hz), 139.96, 129.17, 128.84, 127.96, 126.57, 122.28 (q, *J* = 275.94 Hz), 113.53(q, *J* = 6.3Hz), 111.57 (q, *J* = 1.26 Hz), 106.09, 100.21, 57.25, 40.92, 36.20; HR-ESI-MS *m*/*z*: 429.1038 [M + Na]^+^ (calculated for C_20_H_17_F_3_N_2_NaO_4_, 429.1038).

*1-(2-Chlorophenyl)-3-(7-methoxy-2-oxo-4-(trifluoromethyl)-2H-chromen-6-yl) urea* (**5j**). Yellow solid; yield: 45.0%; m.p. 231 °C; ^1^H-NMR (500 MHz, DMSO) δ: 9.76 (s, 1H), 8.64 (s, 1H), 8.52 (s, 1H), 7.96 (s, 1H), 7.68–7.44 (m, 2H), 7.33 (d, *J* = 6.2 Hz, 1H), 7.27 (s, 1H), 6.83 (s, 1H), 4.03 (s, 3H); ^13^C-NMR (126 MHz, DMSO) δ: 159.34, 152.77, 152.54, 151.06, 140.72, 140.24 (q, *J* = 32.76 Hz), 130.54, 130.08 (q, *J* = 31.5 Hz), 126.78, 122.25 (q, *J* =274.68 Hz), 122.16, 118.80 (q, *J* = 3.6 Hz), 114.32 (q, *J* = 4.0 Hz), 113.83 (q, *J* = 5.2 Hz), 112.27, 106.13, 100.50, 57.44; HR-ESI-MS *m*/*z*: 435.0328, 437.0298 [M + Na]^+^ (calculated for C_18_H_12_ClF_3_N_2_NaO_4_, 435.0335, 437.0306).

*1-(3-Chlorophenyl)-3-(7-methoxy-2-oxo-4-(trifluoromethyl)-2H-chromen-6-yl) urea* (**5k**). Yellow solid; yield: 41.5%; m.p. 241 °C; ^1^H-NMR (500 MHz, DMSO) δ: 9.57 (s, 1H), 8.62 (s, 1H), 8.50 (s, 1H), 7.69 (s, 1H), 7.32–7.25 (m, 2H), 7.21 (d, *J* = 8.0 Hz, 1H), 7.02 (d, *J* = 7.7 Hz, 1H), 6.83 (s, 1H), 4.01 (s, 3H); ^13^C-NMR (126 MHz, DMSO) δ: 159.36, 152.70, 152.54, 151.04, 141.38, 140.24 (q, *J* = 31.2 Hz), 133.77, 131.00, 126.83, 122.27(q, *J* = 275.94 Hz), 122.21, 117.87, 117.03, 113.91 (q, *J* = 6.3 Hz), 112.28 (q, *J* = 1.6 Hz), 106.15, 100.54, 57.47; HR-ESI-MS *m*/*z*: 435.0323, 437.0294 [M + Na]^+^ (calculated for C_18_H_12_ClF_3_N_2_NaO_4_, 435.0335, 437.0306).

*1-(4-Chlorophenyl)-3-(7-methoxy-2-oxo-4-(trifluoromethyl)-2H-chromen-6-yl) urea* (**5l**). White solid; yield: 44.8%; m.p. 243 °C; ^1^H-NMR (500 MHz, DMSO) δ: 9.52 (s, 1H), 8.67 (s, 1H), 8.51 (s, 1H), 7.49 (d, *J* = 8.5 Hz, 2H), 7.39–7.27 (m, 3H), 6.87 (s, 1H), 4.04 (s, 3H); ^13^C-NMR (126 MHz, DMSO) δ: 159.39, 152.75, 152.52, 150.97, 140.34 (q, *J* = 52.92 Hz), 138.85, 129.22, 126.97, 126.06, 122.29 (q, *J* = 287.28 Hz), 120.11, 113.92 (q, *J* = 2.52 Hz), 112.22 (q, *J* = 2.2 Hz), 106.17, 100.53, 57.48; HR-ESI-MS *m*/*z*: 435.0323, 437.0294 [M + Na]^+^ (calculated for C_18_H_12_ClF_3_N_2_NaO_4_, 435.0335, 437.0306).

*1-(3-Fluorophenyl)-3-(7-methoxy-2-oxo-4-(trifluoromethyl)-2H-chromen-6-yl) urea* (**5m**). Yellow solid; yield: 53.8%; m.p. 292 °C; ^1^H-NMR (500 MHz, DMSO) δ: 9.62 (s, 1H), 8.66 (s, 1H), 8.55 (s, 1H), 7.48 (d, *J* = 11.8 Hz, 1H), 7.38–7.27 (m, 2H), 7.11 (d, *J* = 8.1 Hz, 1H), 6.87 (s, 1H), 6.81 (t, *J* = 8.4 Hz, 1H), 4.04 (s, 3H); ^13^C-NMR (126 MHz, DMSO) δ: 162.90 (d, *J* = 240 Hz), 159.37, 152.71, 152.53, 151.01, 141.69 (d, *J* = 11.3 Hz), 140.25 (q, *J* = 32.76 Hz), 130.92 (d, *J* = 9.8 Hz), 126.86, 122.26 (q, *J* = 275.94 Hz), 114.33 (d, *J* = 2.3 Hz), 113.85 (q, *J* = 6.3 Hz), 112.26, 108.90 (d, *J* = 21.42 Hz), 106.14, 105.28 (d, *J* = 26.46 Hz), 100.50, 57.45; HR-ESI-MS *m*/*z*: 419.0620 [M + Na]^+^ (calculated for C_18_H_12_F_4_N_2_NaO_4_, 419.0631).

*1-(2,4-Difluorophenyl)-3-(7-methoxy-2-oxo-4-(trifluoromethyl)-2H-chromen-6-yl) urea* (**5n**). Light yellow solid; yield: 47.6%; m.p. 240 °C; ^1^H-NMR (500 MHz, DMSO) δ: 9.28 (s, 1H), 8.95 (s, 1H), 8.67 (s, 1H), 8.13 (q, *J* = 10, 1H), 7.35–7.26 (m, 2H), 7.05 (t, *J* = 8.6 Hz, 1H), 6.85 (s, 1H), 4.04 (s, 3H); ^13^C-NMR (126 MHz, DMSO) δ: 159.38, 157.39 (dd, *J*_1_ =240 Hz, *J*_2_ =11.25 Hz), 152.86, 152.68 (dd, *J*_1_ = 243.75 Hz, *J*_2_ = 12.5 Hz), 152.64, 151.01, 140.27 (q, *J* = 31.5 Hz), 126.96, 124.24 (dd, *J*_1_ = 7.5 Hz, *J*_2_ = 3.75 Hz), 122.69 (dd, *J*_1_ = 6.25 Hz, *J*_2_ = 2.5 Hz), 122.25 (q, *J* = 273.75 Hz), 113.82 (q, *J* = 6.3 Hz), 112.40 (q, *J* = 2.0 Hz), 111.52 (dd, *J*_1_ = 21.25 Hz, *J*_2_ = 3.75 Hz), 106.12, 104.25 (dd, *J*_1_ = 22.5 Hz, *J*_2_ = 3.75 Hz), 100.51, 57.42; HR-ESI-MS *m*/*z*: 437.0527 [M + Na]^+^ (calculated for C_18_H_11_F_5_N_2_NaO_4_, 437.0537).

*1-(m-Tolyl)-3-(7-methoxy-2-oxo-4-(trifluoromethyl)-2H-chromen-6-yl) urea* (**5o**). Yellow solid, yield: 24.7%; m.p. 217–218 °C; ^1^H-NMR (500 MHz, DMSO) δ: 9.33 (s, 1H), 8.68 (s, 1H), 8.47 (s, 1H), 7.32 (s, 1H), 7.29 (s, 1H), 7.23 (d, *J* = 8.3 Hz, 1H), 7.18 (t, *J* = 7.7 Hz, 1H), 6.86 (s, 1H), 6.82 (d, *J* = 7.3 Hz, 1H), 4.04 (s, 3H), 2.30 (s, 3H); ^13^C-NMR (126 MHz, DMSO) δ: 159.39, 152.83, 152.47, 150.84, 140.30 (q, *J* = 31.5 Hz), 139.80, 138.56, 129.19, 127.22, 123.31, 122.29 (q, *J* = 275.94 Hz), 119.07, 115.82, 113.78 (q, *J* = 5.04 Hz), 112.03 (q, *J* = 1.6 Hz), 106.15, 100.46, 57.44, 21.70; HR-ESI-MS *m*/*z*: 415.0874 [M + Na]^+^ (calculated for C_19_h_15_f_3_n_2_nao_4_, 415.0882).

*1-(3-(Trifluoromethyl)phenyl)-3-(7-methoxy-2-oxo-4-(trifluoromethyl)-2H-chromen-6-yl) urea* (**5p**). Light yellow solid; yield 39.0%; m.p. 219 °C; ^1^H-NMR (500 MHz, DMSO) δ: 9.24 (s, 1H), 9.06 (s, 1H), 8.66 (s, 1H), 8.08 (d, *J* = 8.1 Hz, 1H), 7.46 (d, *J* = 7.7 Hz, 1H), 7.35–7.28 (m, 2H), 7.07 (t, *J* = 7.3 Hz, 1H), 6.86 (s, 1H), 4.05 (s, 3H); ^13^C-NMR (126 MHz, DMSO) δ: 159.40, 152.95, 151.10, 140.26(q, *J* = 31.5 Hz), 136.13, 129.76, 127.98, 126.94, 124.36, 123.32, 123.17, 122.27 (q, *J* = 275.94 Hz), 118.98, 113.84 (q, *J* = 6.3 Hz), 112.99, 106.11, 100.56, 57.43; HR-ESI-MS *m*/*z*: 469.0593 [M + Na]^+^ (calculated for C_19_H_12_F_6_N_2_NaO_4_, 469.0599).

*1-(4-(Trifluoromethyl)phenyl)-3-(7-methoxy-2-oxo-4-(trifluoromethyl)-2H-chromen-6-yl) urea* (**5q**). Yellow solid; yield: 16.9%; m.p. 212–213 °C; ^1^H-NMR (500 MHz, DMSO) δ: 9.80 (s, 1H), 8.67 (s, 1H), 8.59 (s, 1H), 7.66 (q, *J* = 9.0 Hz, 4H), 7.29 (s, 1H), 6.86 (s, 1H), 4.04 (s, 3H); ^13^C-NMR (126 MHz, DMSO) δ : 159.35, 152.62, 152.56, 151.08, 143.58, 140.24 (q, *J* = 32.76 Hz), 126.74, 126.65 (q, *J* = 3.78 Hz), 125.00 (q, *J* = 270 Hz), 122.47 (q, *J* = 31.5 Hz), 122.26 (q, *J* = 275.94 Hz), 118.28, 113.88 (q, *J* = 6.3 Hz), 112.34, 106.14, 100.54, 57.47; HR-ESI-MS *m*/*z*: 469.0593 [M + Na]^+^ (calculated for C_19_H_12_F_6_N_2_NaO_4_, 469.0599).

*1-(2-Methoxyphenyl)-3-(7-methoxy-2-oxo-4-(trifluoromethyl)-2H-chromen-6-yl) urea* (**5r**). Light yellow solid, yield: 30.9%; m.p. 152 °C; ^1^H-NMR (500 MHz, DMSO) δ: 9.15 (s, 1H), 9.01 (s, 1H), 8.69 (s, 1H), 8.09 (dd, *J*_1_ = 7.9 Hz, *J*_2_ = 1.1 Hz, 1H), 7.27 (s, 1H), 7.06–6.95 (m, 2H), 6.93–6.87 (m, 1H), 6.84 (s, 1H), 4.03 (s, 3H), 3.88 (s, 3H); ^13^C-NMR (126 MHz, DMSO) δ: 159.42, 153.15, 152.90, 150.89, 148.76, 140.35 (q, *J* = 32.76 Hz), 128.81, 127.36, 122.77, 122.28 (q, *J* = 275.94 Hz), 120.94, 119.89, 113.65 (q, *J* = 6.3 Hz), 112.69 (q, *J* = 1.9 Hz), 111.34, 106.08, 100.40, 57.28, 56.17; HR-ESI-MS *m*/*z*: 431.0826 [M + Na]^+^ (calculated for C_19_H_15_F_3_N_2_NaO_5_, 431.0831).

*1-(3-Methoxyphenyl)-3-(7-methoxy-2-oxo-4-(trifluoromethyl)-2H-chromen-6-yl) urea* (**5s**). Yellow solid; yield: 37.8%; m.p. 196 °C; ^1^H-NMR (500 MHz, DMSO) δ: 9.40 (s, 1H), 8.67 (s, 1H), 8.47 (s, 1H), 7.29 (s, 1H), 7.20 (t, *J* = 8.1 Hz, 1H), 7.11 (t, *J* = 2.1 Hz, 1H), 6.98 (dd, *J*_1_ = 8.0 Hz, *J*_2_ = 1.1 Hz, 1H), 6.86 (s, 1H), 6.58 (dd, *J*_1_ = 8.2 Hz, *J*_2_ =2.3 Hz, 1H), 4.04 (s, 3H), 3.75 (s, 3H); ^13^C-NMR (126 MHz, DMSO) δ: 160.21, 159.39, 152.78, 152.52, 150.90, 141.06, 140.29(q, *J* = 32.76 Hz), 130.16, 127.12, 122.29 (q, *J* = 275.94 Hz), 113.82 (q, *J* = 6.3Hz) 112.13 (q, *J* = 1.9 Hz), 111.00, 107.86, 106.16, 104.50, 100.49, 57.45, 55.46; HR-ESI-MS *m*/*z*: 431.0829 [M + Na]^+^ (calculated for C_19_H_15_F_3_N_2_NaO_5_, 431.0831).

*1-(4-Methoxyphenyl)-3-(7-methoxy-2-oxo-4-(trifluoromethyl)-2H-chromen-6-yl) urea* (**5t**). Light yellow solid; yield: 37.7%; m.p. 212–213 °C; ^1^H-NMR (500 MHz, DMSO) δ: 9.21 (s, 1H), 8.68 (s, 1H), 8.40 (s, 1H), 7.36 (d, *J* = 7.9 Hz, 2H), 7.28 (s, 1H), 6.91–6.83 (m, 3H), 4.03 (s, 3H), 3.72 (s, 3H); ^13^C-NMR (126 MHz, DMSO) δ: 159.43, 155.10, 153.01, 152.42, 150.75, 140.32 (q, *J* = 31.5 Hz), 132.85, 127.36, 122.28 (q, *J* = 275.94 Hz), 120.42, 114.55, 113.76 (q, *J* = 5.04 Hz), 111.94 (q, *J* = 1.1 Hz), 106.16, 100.42, 57.43, 55.66; HR-ESI-MS *m*/*z*: 431.0826 [M + Na]^+^ (calculated for C_19_H_15_F_3_N_2_NaO_5_, 431.0831).

*1-(4-(Trifluoromethoxy) phenyl)-3-(7-methoxy-2-oxo-4-(trifluoromethyl)-2H-chromen-6-yl) urea* (**5u**). Light yellow solid; yield: 43.7%; m.p. 209 °C; ^1^H-NMR (500 MHz, DMSO) δ: 9.61 (s, 1H), 8.70 (s, 1H), 8.54 (s, 1H), 7.62–7.56 (m, 2H), 7.39–7.29 (m, 3H), 6.88 (s, 1H), 4.07 (s, 3H); ^13^C-NMR (126 MHz, DMSO) δ: 159.37, 152.79, 152.52, 150.98, 143.24 (q, *J* = 2.52 Hz), 140.28 (q, *J* = 31.5 Hz), 139.12, 126.95, 122.29, 122.27 (q, *J* = 273.75 Hz), 120.67 (q, *J* = 273.75 Hz), 119.84, 113.86 (q, *J* = 5.04 Hz), 112.25, 106.16, 100.52, 57.47; HR-ESI-MS *m*/*z*: 463.3020 [M + Na]^+^ (calculated for C_19_H_13_F_6_N_2_O_5_, 463.3024).

### 3.2. Determination of UV Absorption and Fluorescence Spectrum

The title compounds were dissolved in methanol to prepare 1 and 10 μg·mL^−1^ solutions, and the same concentrations of the reference compound, quinine sulfate, were obtained using 0.1 mol·L^−1^ H_2_SO_4_ aqueous solution as the solvent. The 10 μg·mL^−1^ solutions of all the compounds were used to record UV absorption spectra. The intersection point wavelength of UV spectrums of title compound and quinine sulfate that lay between 300 to 400 nm was selected as the excitation wavelength to record the fluorescence spectrum. The 1 μg·mL^−1^ solutions were used to determine the fluorescence to ensure the absorbance was less than 0.05. The refraction indexes (n) of the 1 μg·mL^−1^ solutions were also measured to calculate the Φ_f_. Δλ and Φ_f_ were calculated according to the following formulas [56]:

Δλ = λ_max_^em^ − λ_max_^ab^ (1)

Φ_f_ = (n^2^_x_·D_x_·Φ_fstd_)/(n^2^_std_·D_std_), (2)

where n_x_, n_std_—refraction index of title compound and reference compound, respectively; D_x_, D_std_—fluorescence peak area of title compound and reference compound, respectively; Φ_fstd_—fluorescence quantum efficiency of reference compound with value of 0.55.

### 3.3. Procedures for Activities Evaluation

#### 3.3.1. Herbicidal Activity

The inhibition of the title compounds against the taproot and caulis growth of dicotyledonous weed *A. retroflexus* and monocotyledonous weed *D. sanguinalis* were determined in vitro [57]. Two-milligram and 10-mg portions of the title compounds were dissolved in 1 mL of acetone to give 2 and 10 g·L^−1^ of stock solutions, respectively. A suspension of 0.3 g agar powder in 60 mL distilled water was microwave heated to melt. Then 0.6 mL of the stock solution was added to 60 mL of the melting agar of about 50 °C to achieve the required concentration. Then, 10 mL of the compound–agar medium was poured into a small beaker (10 mL) and cooled to room temperature. After that, 10 just-germinated weed seeds were planted onto the surface of the medium. Then the beaker was sealed with a piece of plastic wrap with several small holes made in it by a needle. Finally, cultivation was achieved in an illumination incubator (28 ± 1 °C, 50–55% relative humidity, 12 h L: 12 h D) for seven days. Acetone was used as a blank control, and Acetochlor was used as a positive control. Each treatment was repeated three times. After cultivation, the taproot and caulis lengths of the weed seedlings were measured, and the growth inhibitory rate of the treatment to the untreated control was determined.

#### 3.3.2. Antifungal Activity

The in vitro antifungal activities of the title compounds against the mycelium growth of four phytopathogenic fungi, *V. mali*, *B. cinerea*, *F. oxysporium*, and *C. glecosporioides*, were tested [57]. The stock solutions of the title compounds were prepared according to the procedures in Section 3.3.1. Then 1 mL of the stock solutions was added to 100 mL portions of melted (~50 °C) potato dextrose agar (PDA), and the mixtures were fully shaken to obtain the required concentrations of compound mixed medium. Then 5 mL portions of the mixed medium were poured into 6 cm petri dishes and allowed to cool to ambient temperature to form solid plates. After that, one 4 mm activated mycelium disk was inoculated on each of the PDA plates and incubated at 28 °C in the dark for a sufficient period. Acetone was used as a blank control, and Carbendazim was used as a positive control. Each treatment was repeated three times. The radius (mm) of the fungus mycelium settlements was measured and the growth inhibition rate relative to the untreated control was calculated.

## 4. Conclusions

In conclusion, 21 novel trifluoromethylcoumarinyl urea derivatives have been designed and synthesized according to the principle of bioactive substructure combination. The fluorescence spectrums of the title compounds showed that most of them glow green with λ_max_^em^ of 500–517 nm when illuminated by ultraviolet light, while compounds **5r**, **5s**, **5u**, and **5l** glow violet with a λ_max_^em^ of 381–443 nm. However, the fluorescent intensity of all the title compounds is weaker than the standard reference compound, quinine sulfate. Moreover, the pesticidal activities evaluation of the title compounds indicated that compound **5f** against the caulis of *A. retroflexus* and compounds **5j** and **5l** against the taproot of *D. sanguinalis* are equivalent to the commercial herbicide Acetochlor. Nine of the title compounds are more antifungal than commercial fungicide Carbendazim against *B. cinerea*.

## Figures and Tables

**Table 1:** Properties of UV absorption and fluorescence of title compounds.

Compd.	λ_max_^ab^ (nm)	A	ε_max_ × 10^3^ (L·mol^−1^·cm^−1^)	λ_max_^em^ (nm)	Δλ (nm)	Φ_f_
**5a**	369	0.216	7.13	512	143	0.114
**5b**	369	0.186	6.40	511	142	0.110
**5c**	374	0.180	6.19	516	143	0.065
**5d**	373	0.188	6.73	517	145	0.044
**5e**	360	0.189	7.30	513	154	0.087
**5f**	369	0.160	5.92	512	144	0.064
**5g**	370	0.154	5.92	513	143	0.053
**5h**	370	0.150	6.90	512	142	0.070
**5i**	366	0.129	5.24	510	144	0.082
**5j**	367	0.182	7.50	507	141	0.098
**5k**	367	0.171	7.05	509	143	0.024
**5l**	369	0.174	7.17	381	12	0.028
**5m**	363	0.162	6.71	506	143	0.013
**5n**	367	0.183	7.25	500	134	0.020
**5o**	367	0.164	6.43	-	-	-
**5p**	363	0.166	7.40	504	141	0.041
**5q**	367	0.134	5.98	503	137	0.115
**5r**	372	0.143	5.84	443	72	0.005
**5s**	363	0.134	5.47	396	33	0.013
**5t**	365	0.136	5.55	-	-	-
**5u**	367	0.166	7.67	387	21	0.013

- no fluorescence was detected.

## Supplementary Materials

Supplementary materials are available online. Figures S1–S71.
