# Supplementary material for: Novel Trifluoromethylcoumarinyl Urea Derivatives: Synthesis, Characterization, Fluorescence, and Bioactivity
Source: Molecules. 2018 Mar 7;23(3):600. doi: 10.3390/molecules23030600 (PMC6017327; doi:10.3390/molecules23030600)
Supplement: Supplementary file 1 [file molecules-23-00600-s001.pdf]

Supplementary Materials

Novel Trifluoromethylcoumarinyl Urea Derivatives: Synthesis, Characterization, Fluorescence and Bioactivity

Li-Li Qiao and Shuang-Hong Hao*

Research Center of Agro-bionic Engineering & Tech. of Shandong Province, College of Chemistry & Pharm., Qingdao Agricultural University, Qingdao 266109, China; qiaolili07@163.com (L.-L. Q.)

* Correspondence: doublered74@sina.com; Tel.: +86-532-8803-0522

^{1} H NMR of **1-4** --S2-S3

^{1} H NMR of **5a-5u** --S4-S14

^{13}C NMR of **5a-5u** --S15-S25

HR-ESI-MS of **1-4** --S26-S27

HR-ESI-MS of **5a-5u** --S28-S34

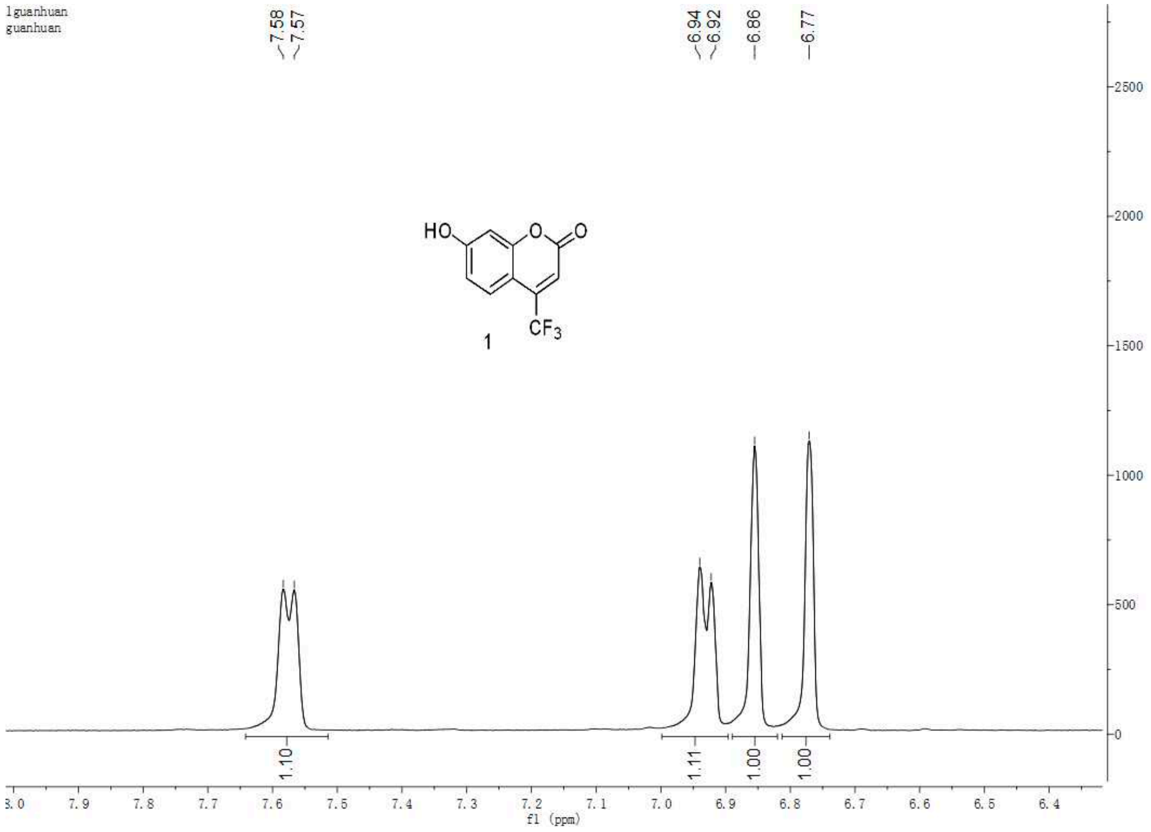

Figure S1. ^{1}H NMR of **1**

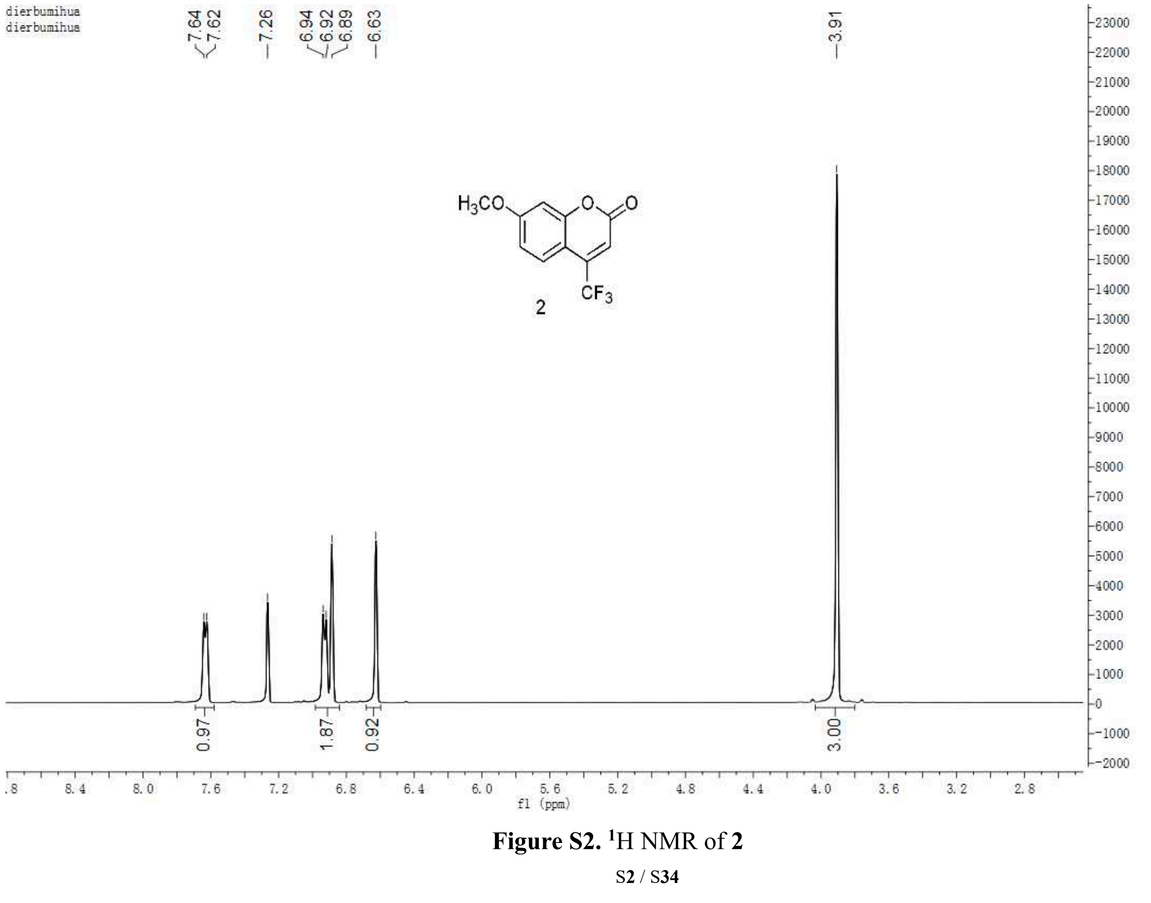

Figure S2. ^{1}H NMR of **2**

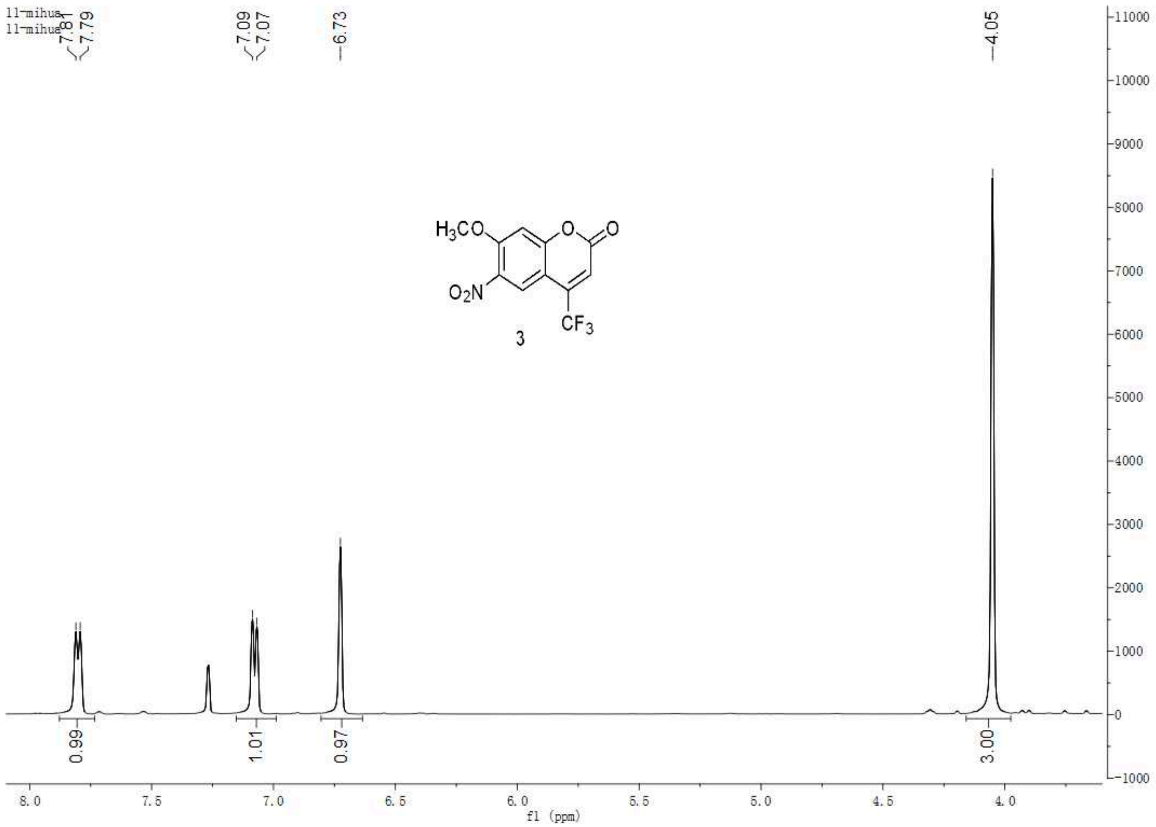

Figure S3. ^{1}H NMR of 3

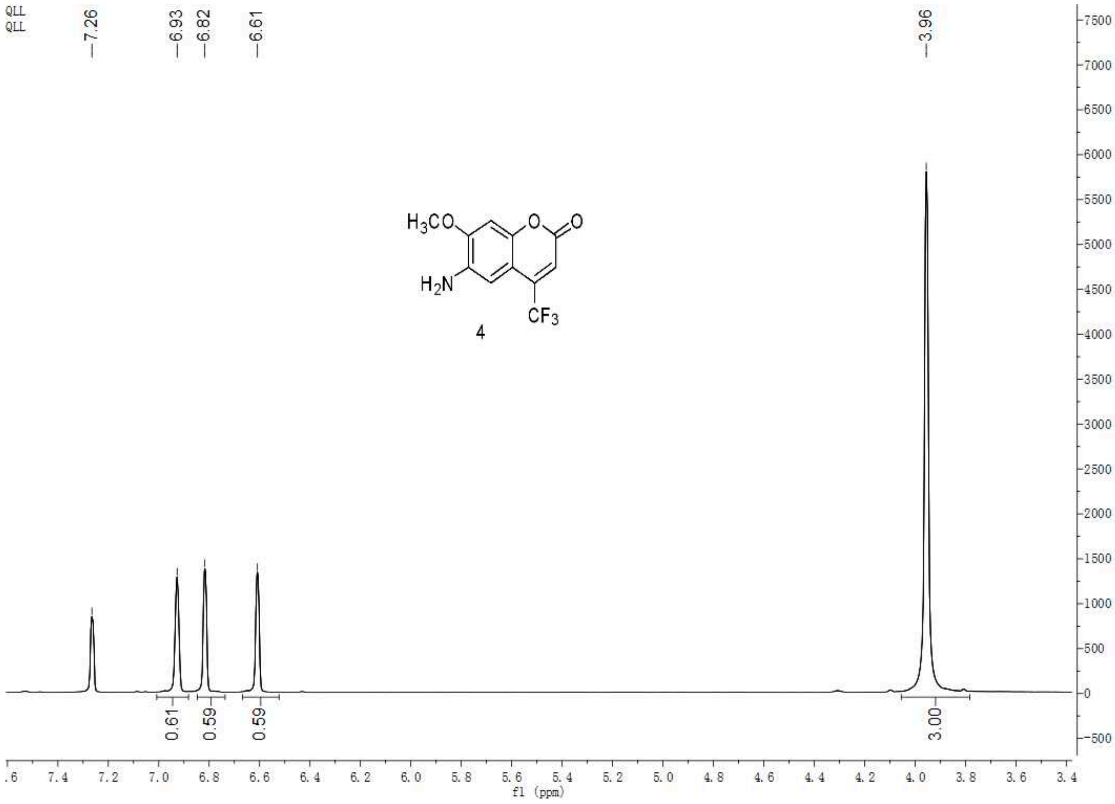

Figure S4. ^{1}H NMR of 4

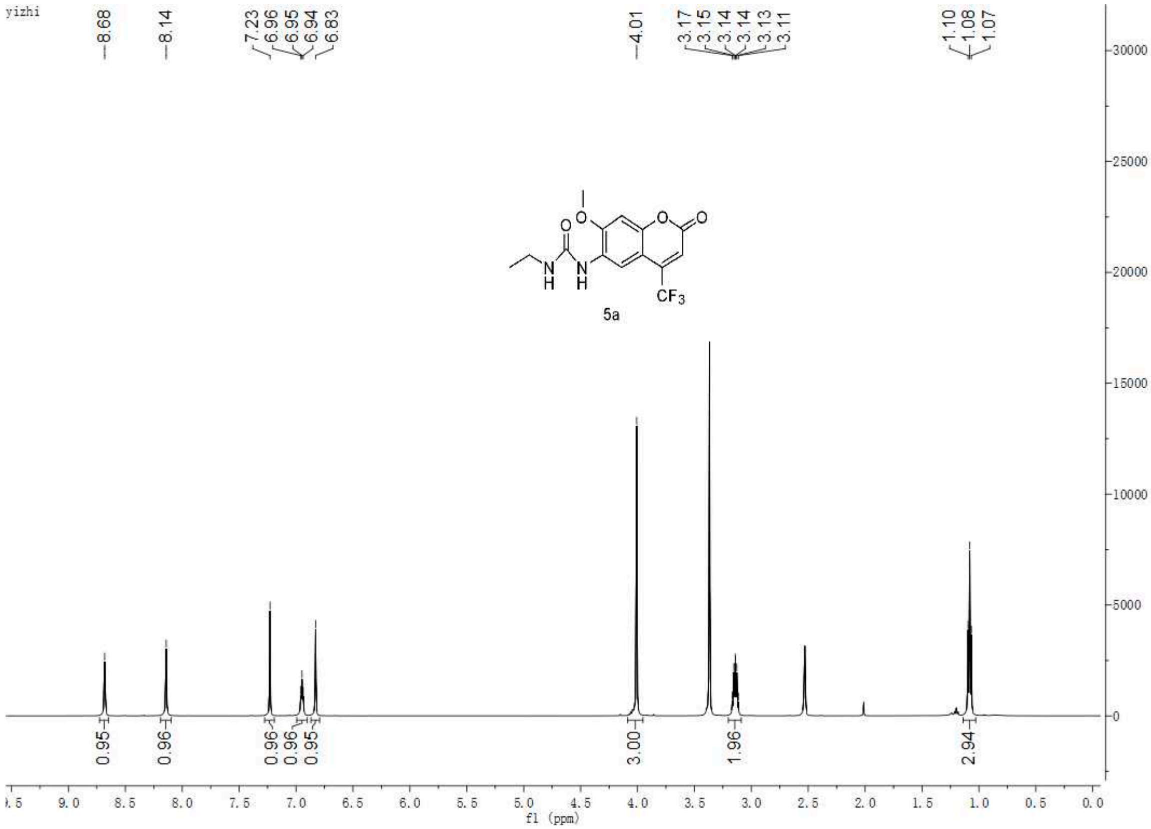

Figure S5. ^{1}H NMR of 5a

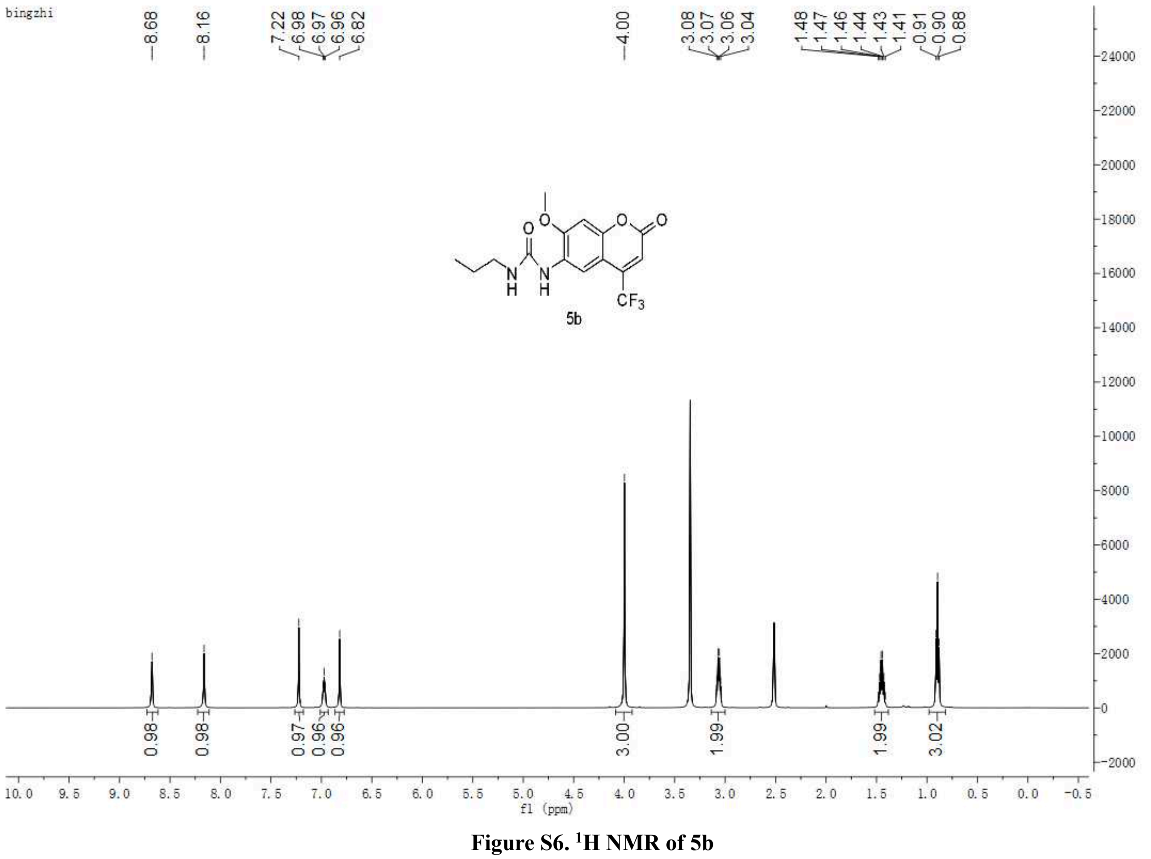

Figure S6. ^{1}H NMR of 5b

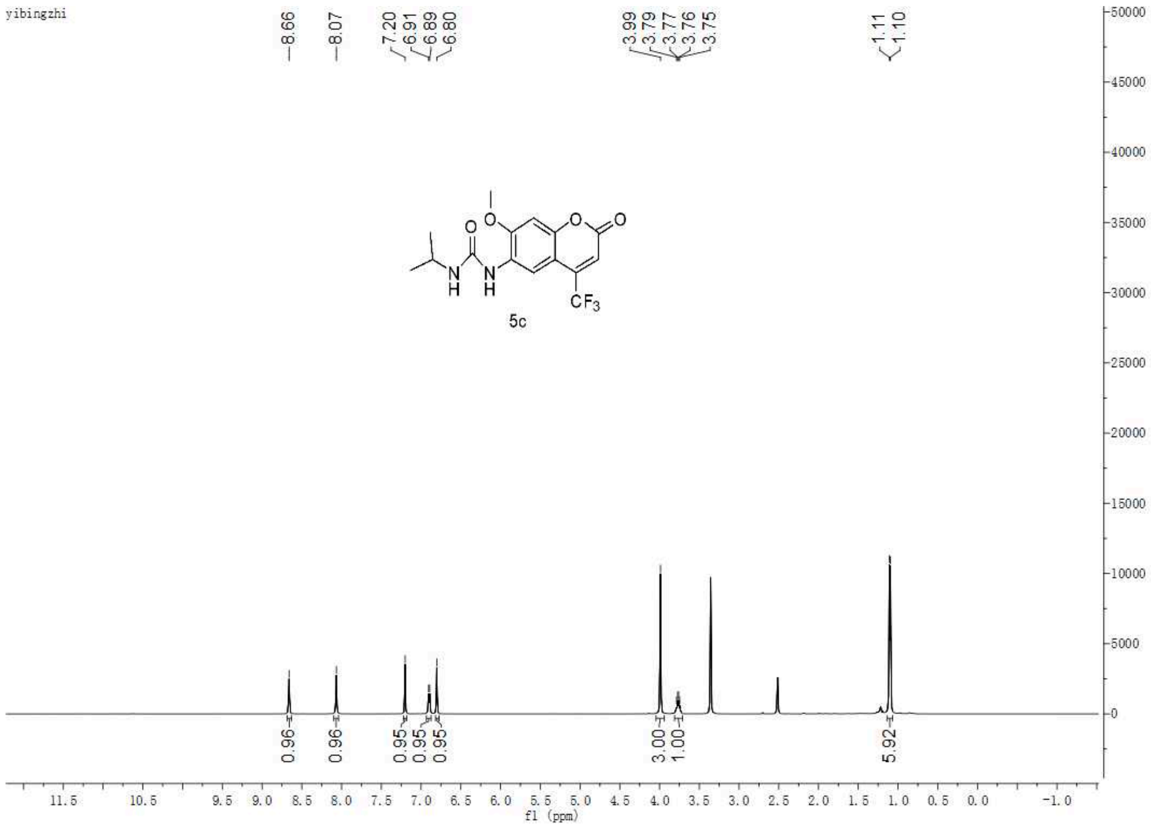

Figure S7. **^{1}H NMR of 5c**

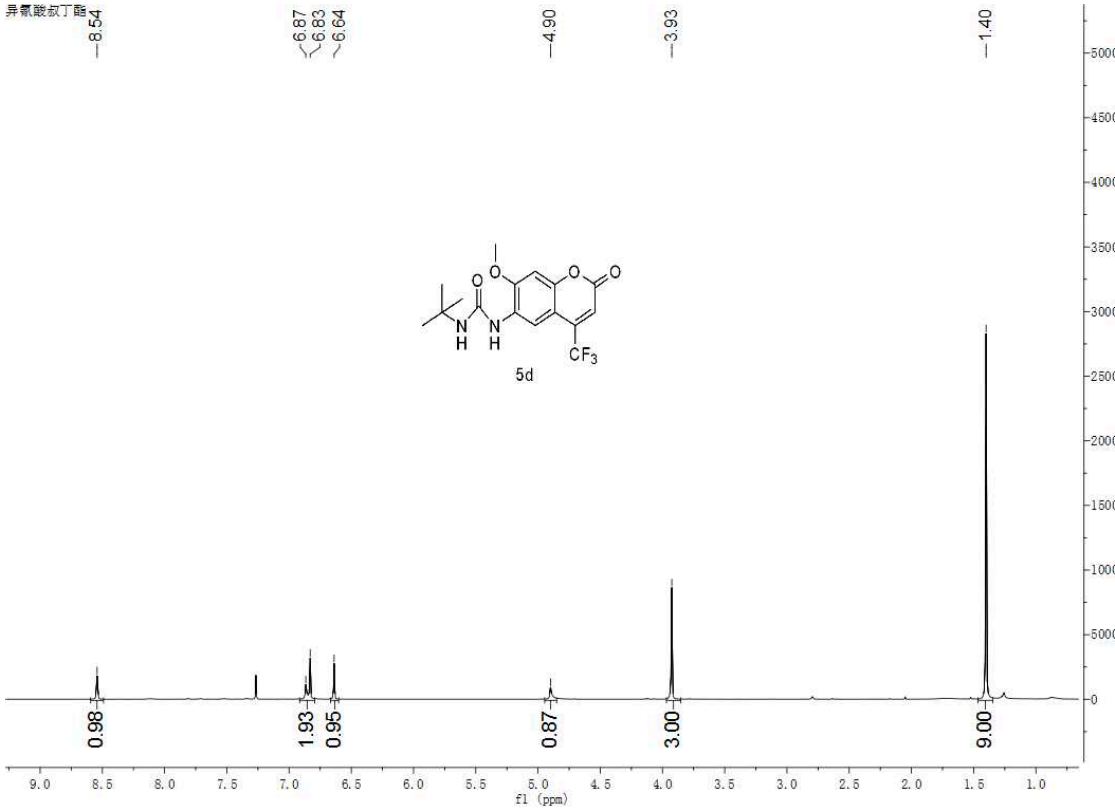

Figure S8. **^{1}H NMR 5d**

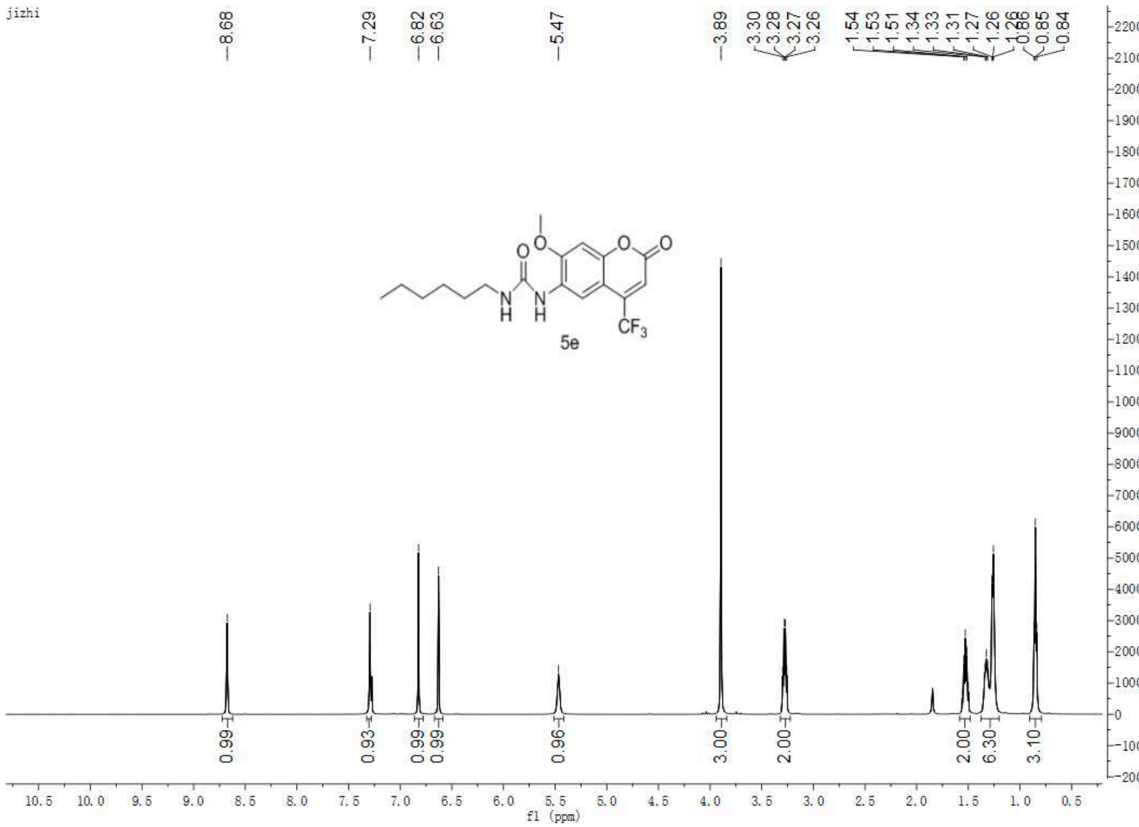

Figure S9. ^{1}H NMR of 5e

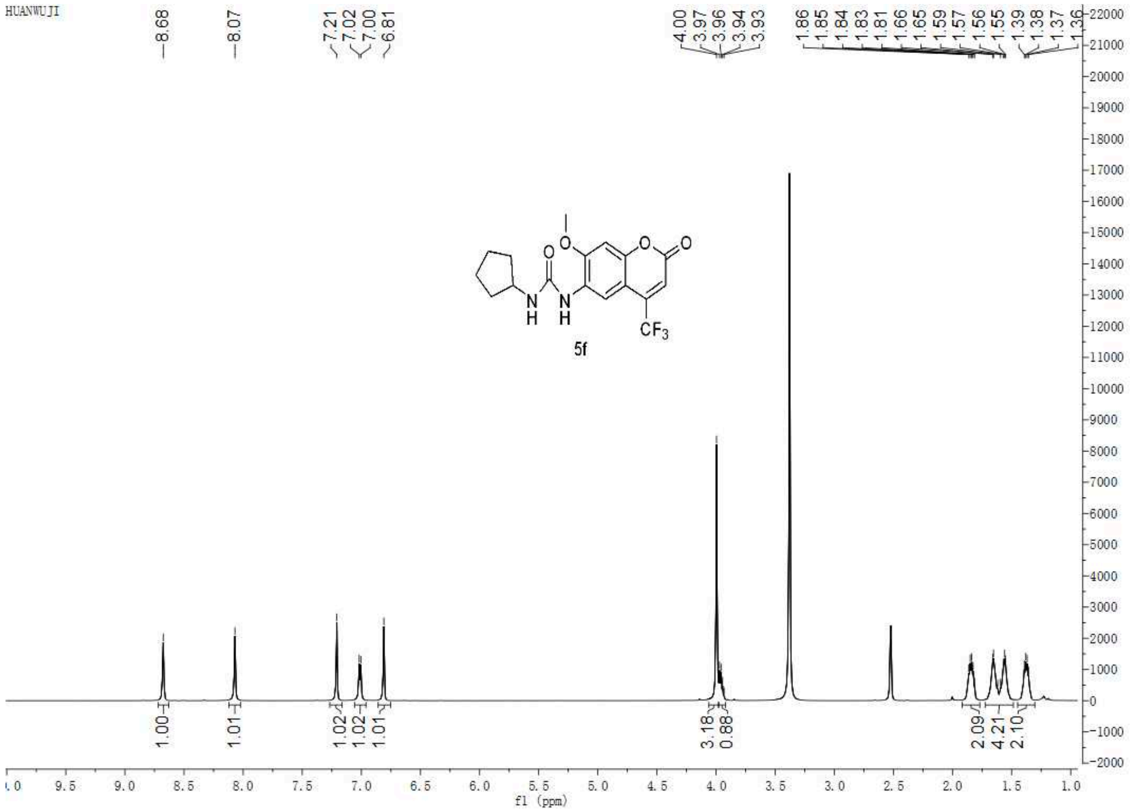

Figure S10. ^{1}H NMR of 5f

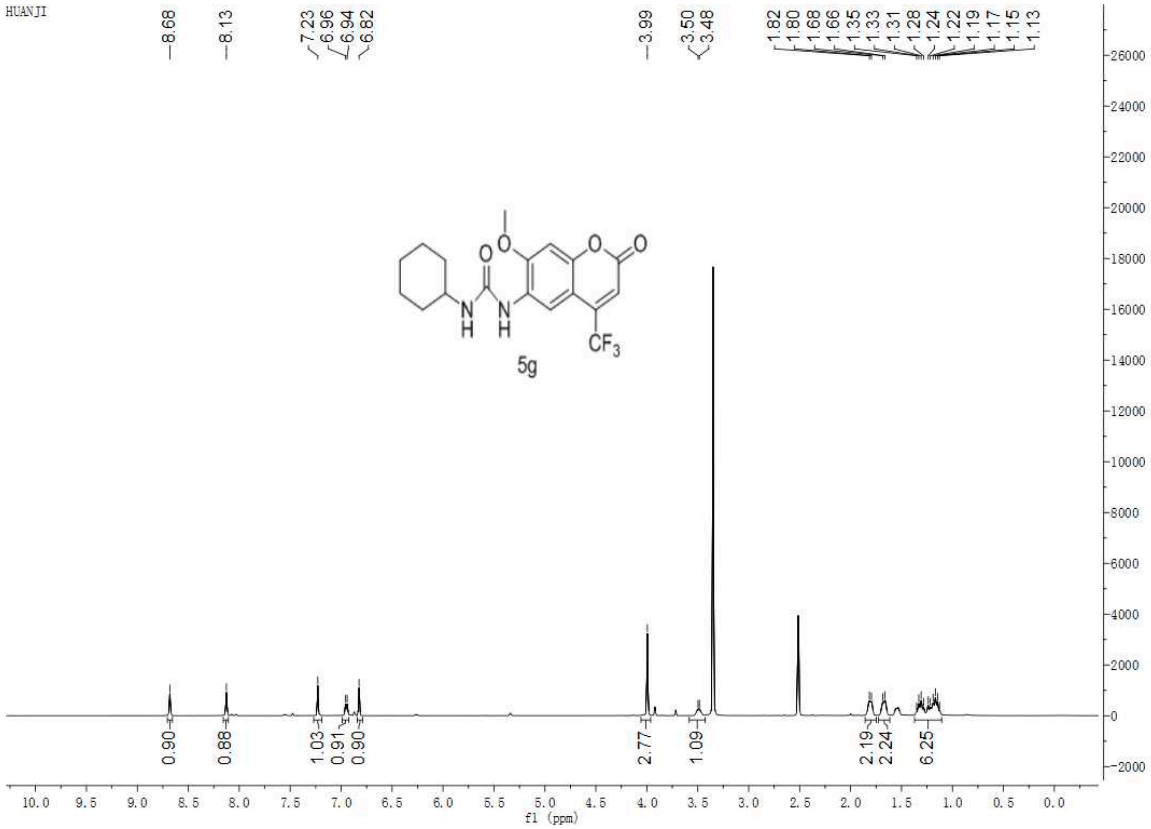

Figure S11. ^{1}**H NMR of 5g**

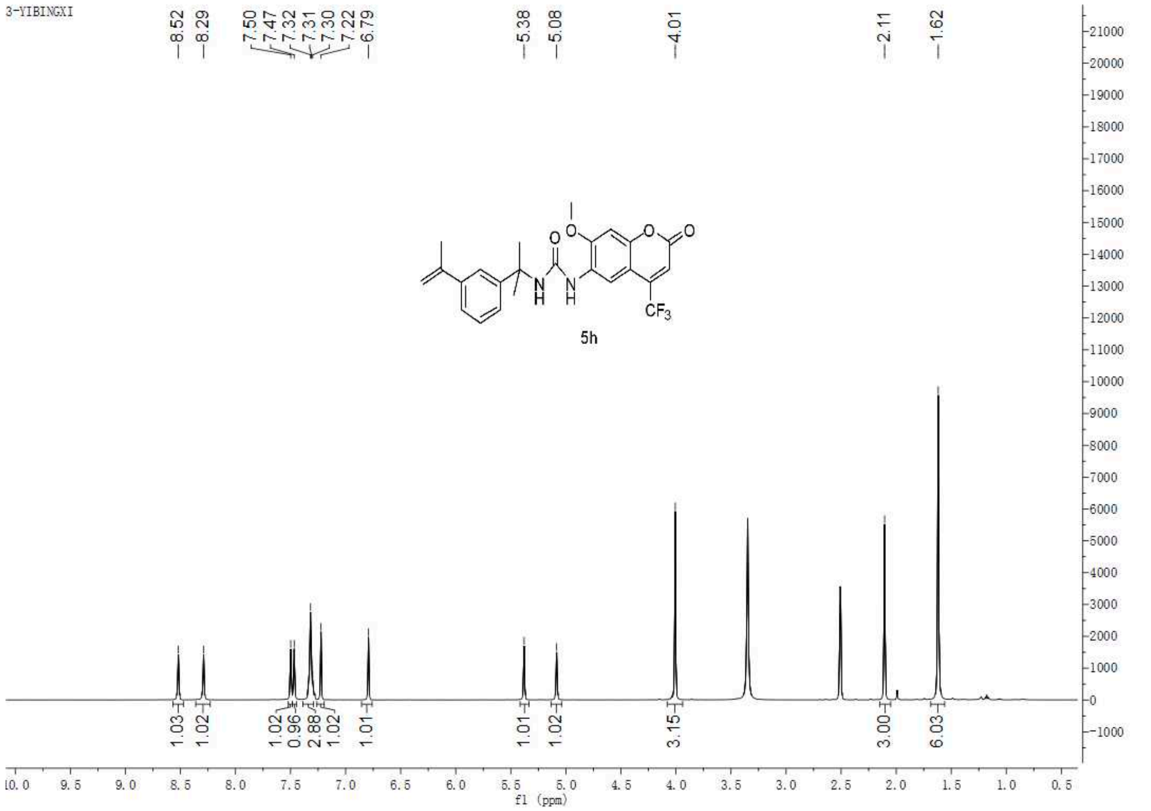

Figure S12. ^{1}**H NMR of 5h**

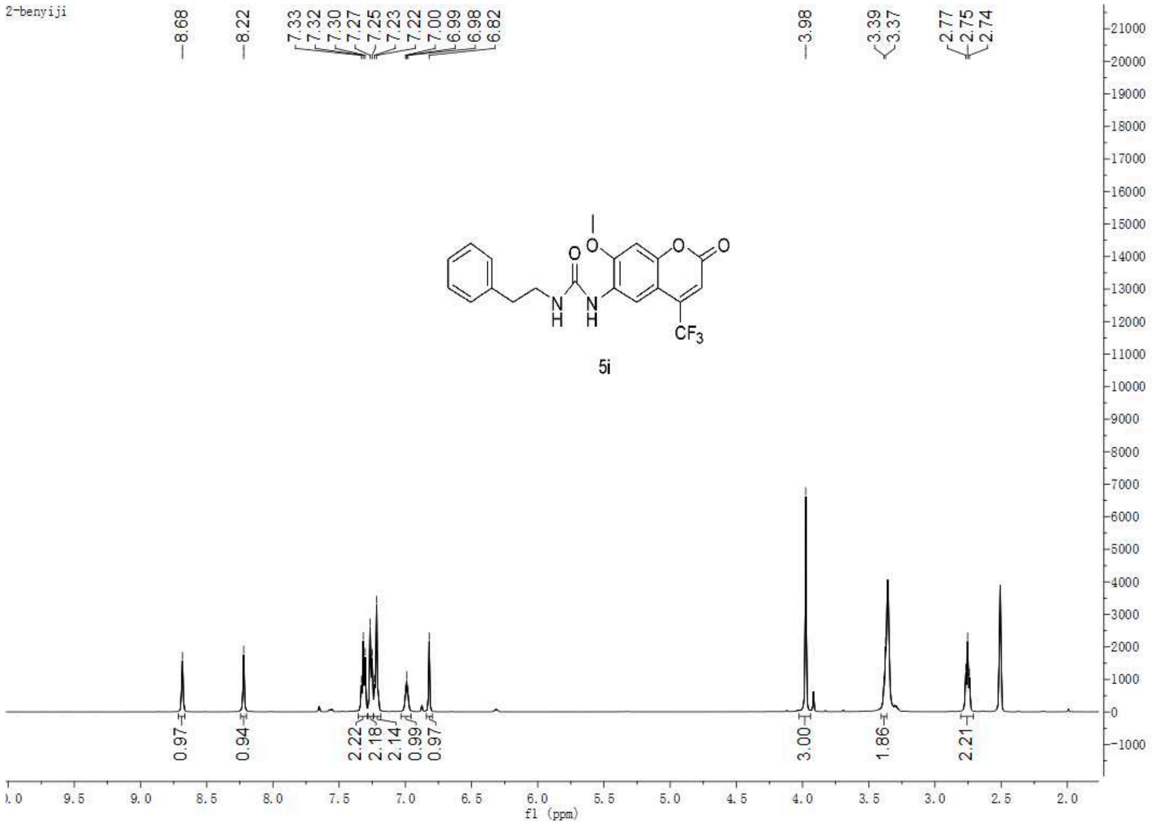

Figure S13. ^{1}H NMR of 5i

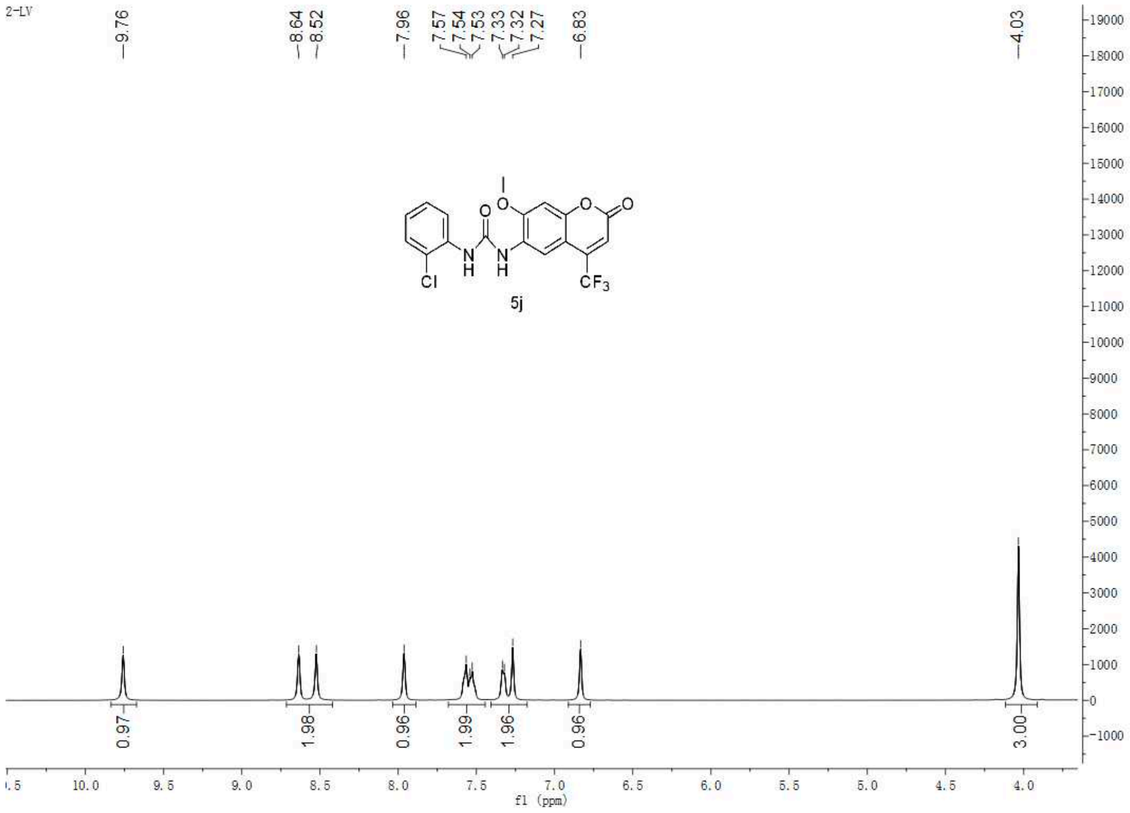

Figure S14. ^{1}H NMR of 5j

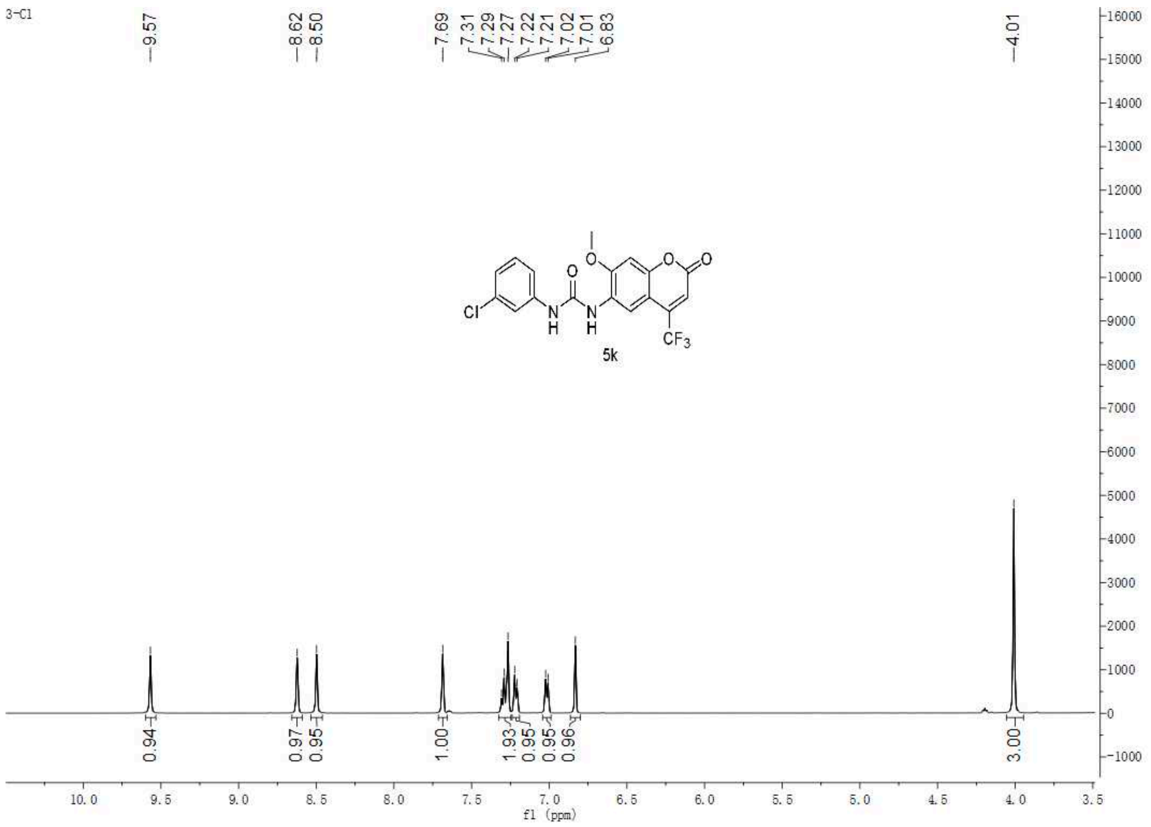

Figure S15. ^{1}H NMR of 5k

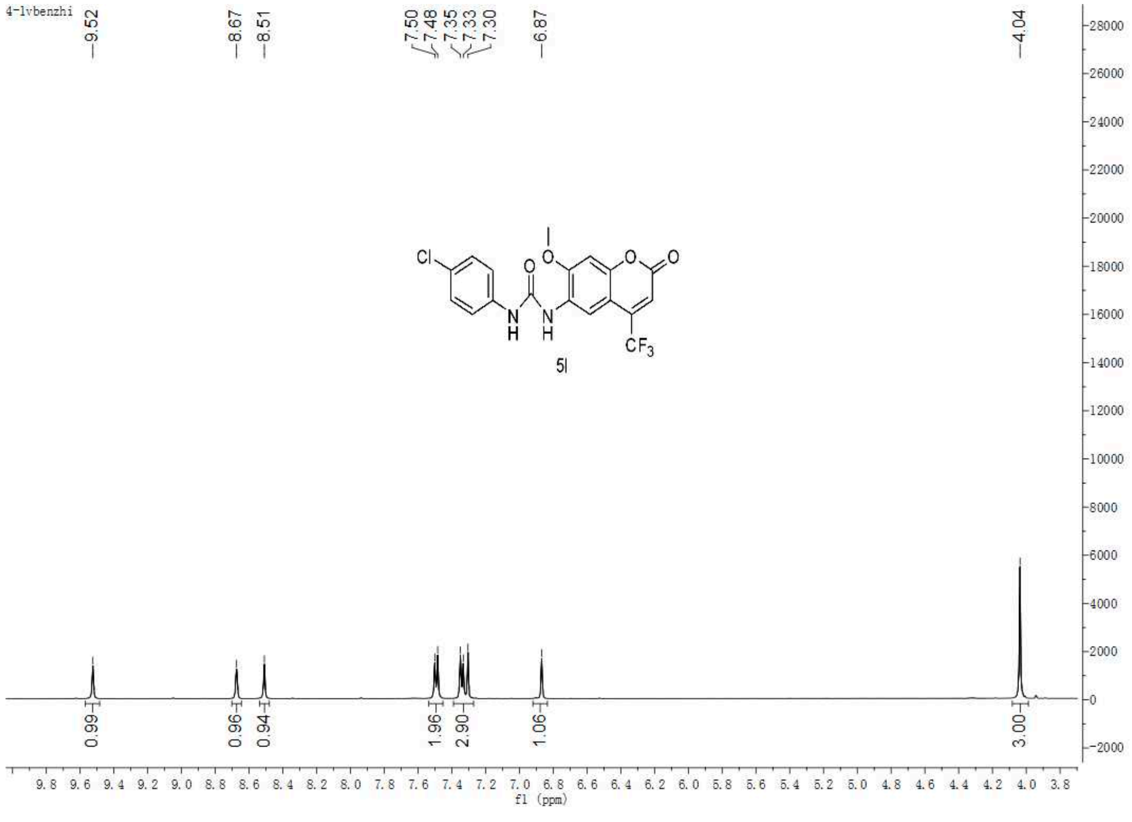

Figure S16. ^{1}H NMR of 5l

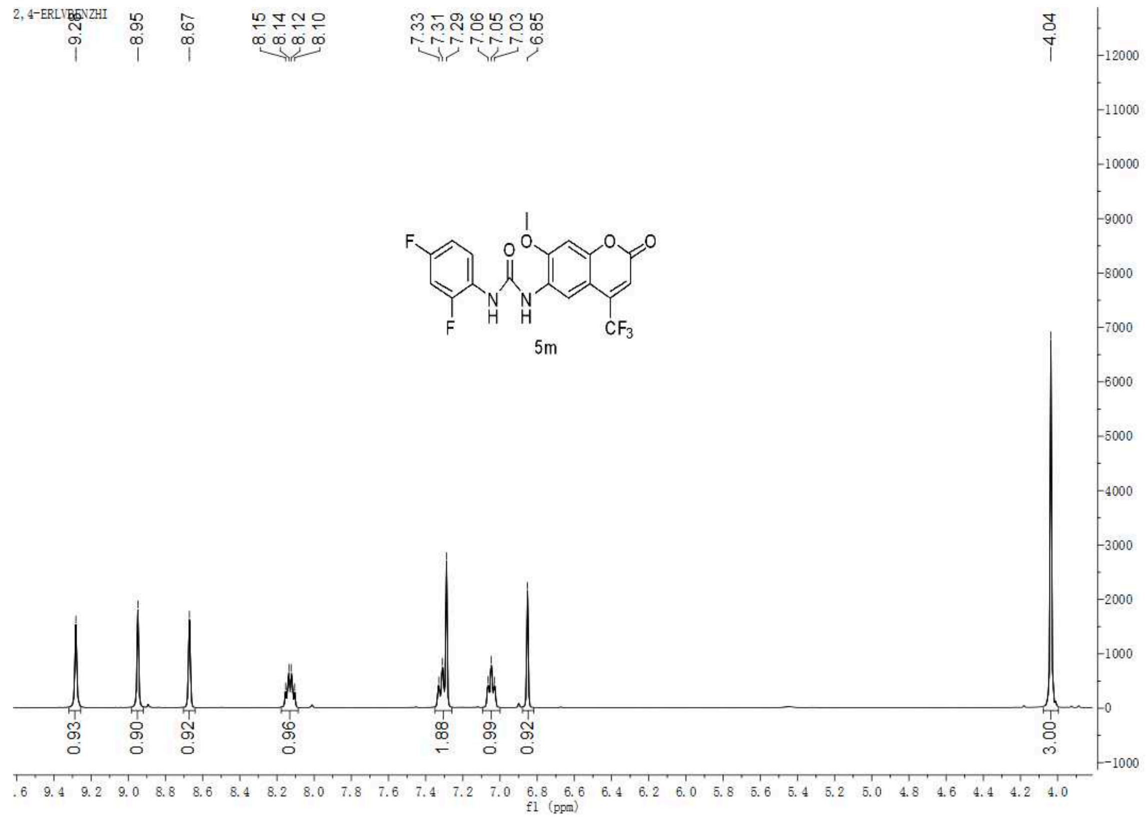

Figure S17. ¹H NMR of 5m

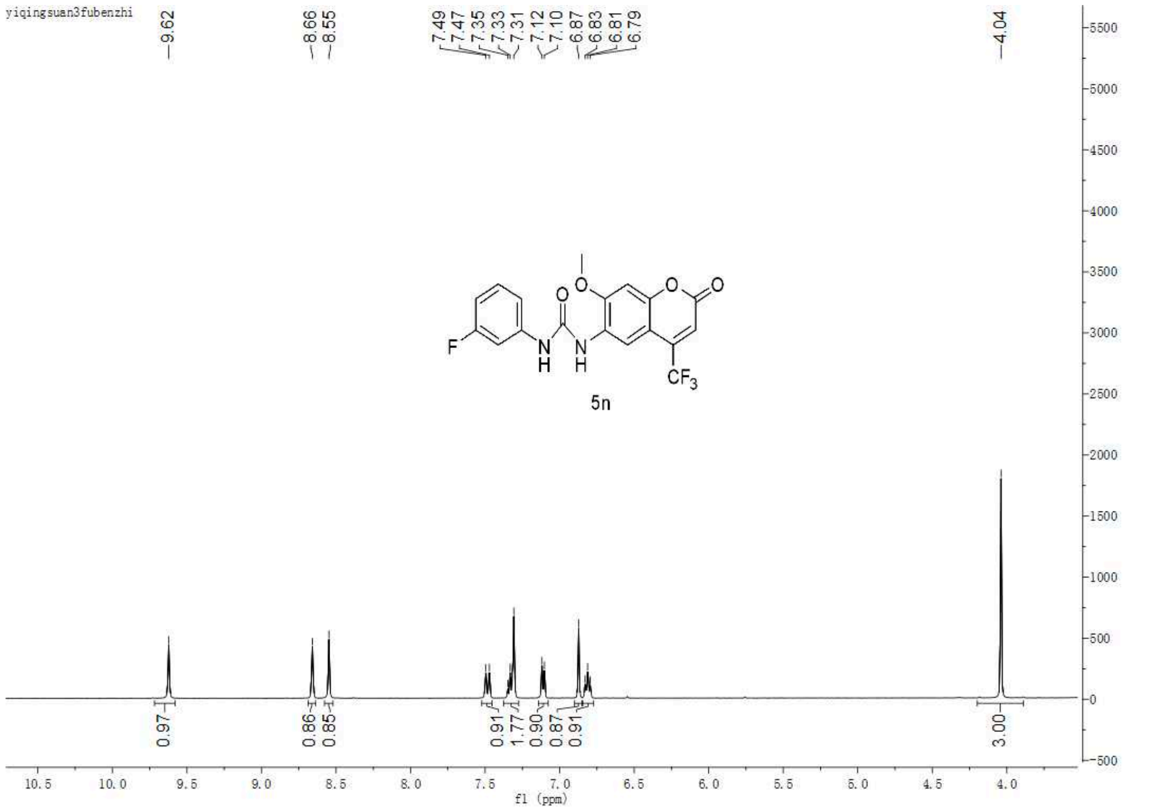

Figure S18. ¹H NMR of 5n

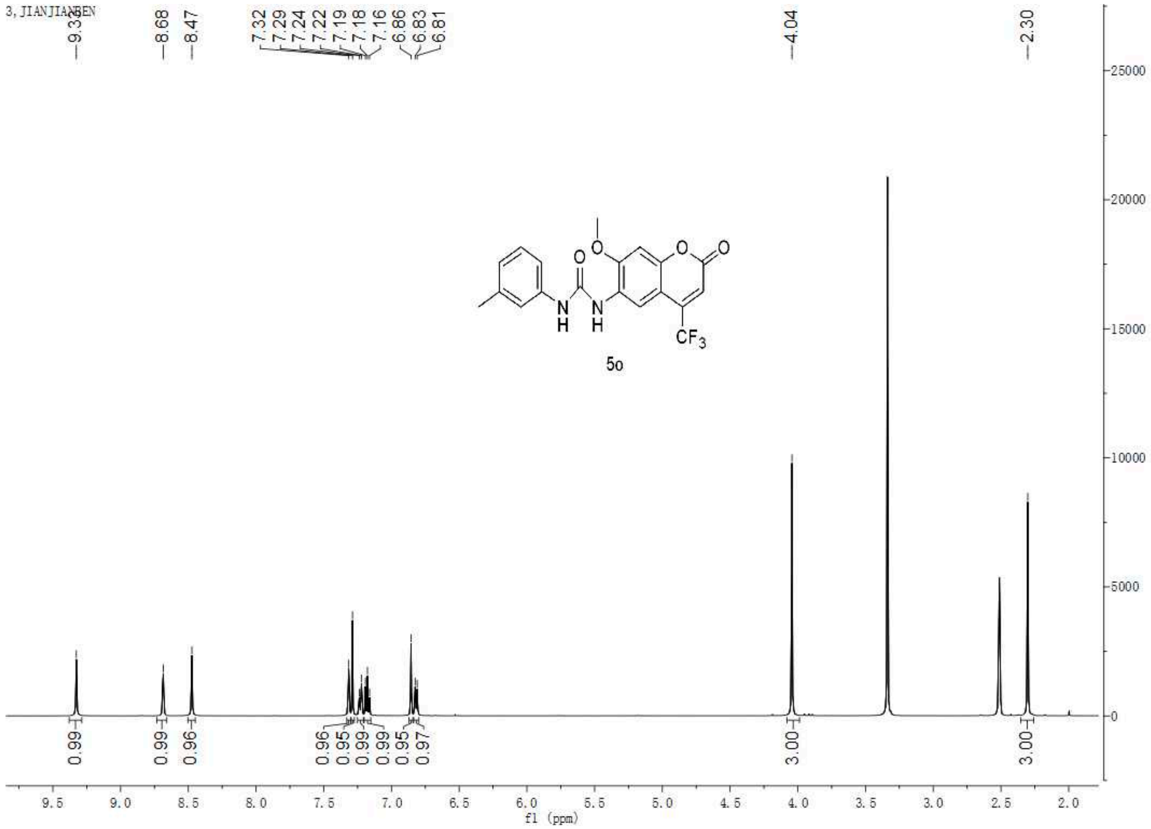

Figure S19. ¹H NMR of 5o

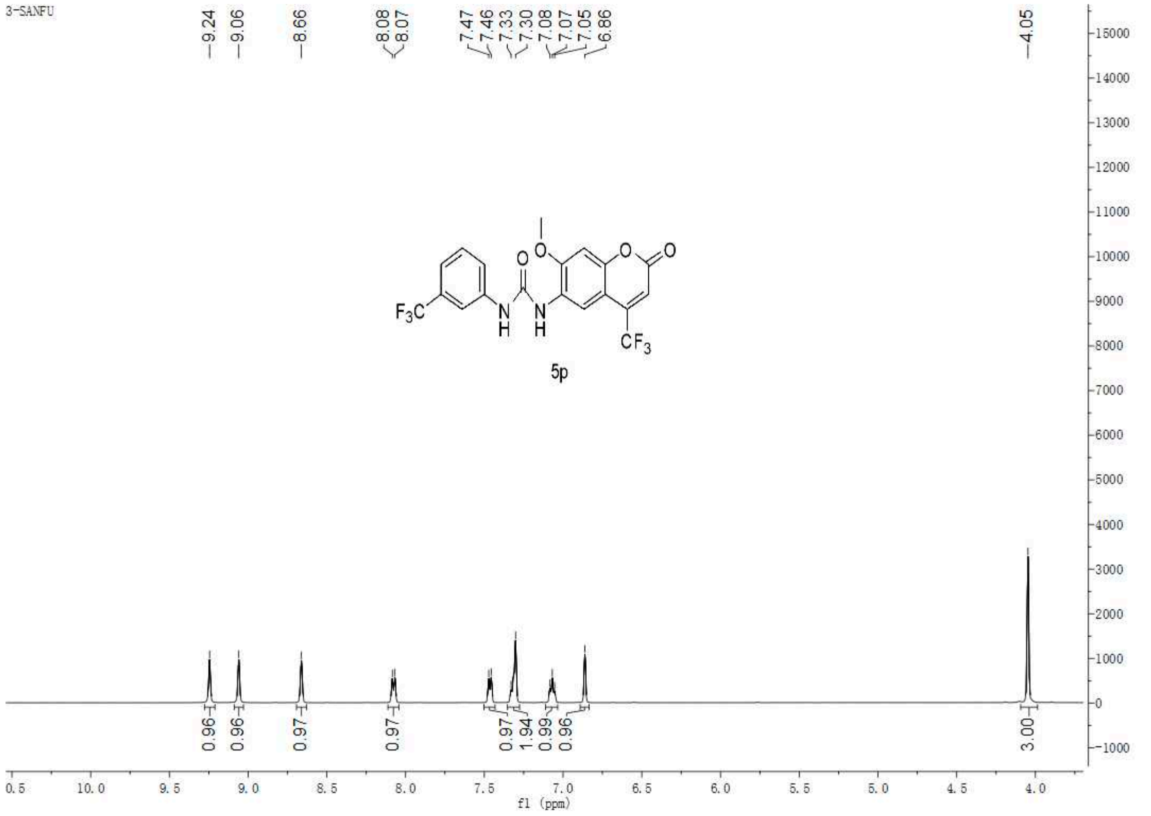

Figure S20. ¹H NMR of 5p

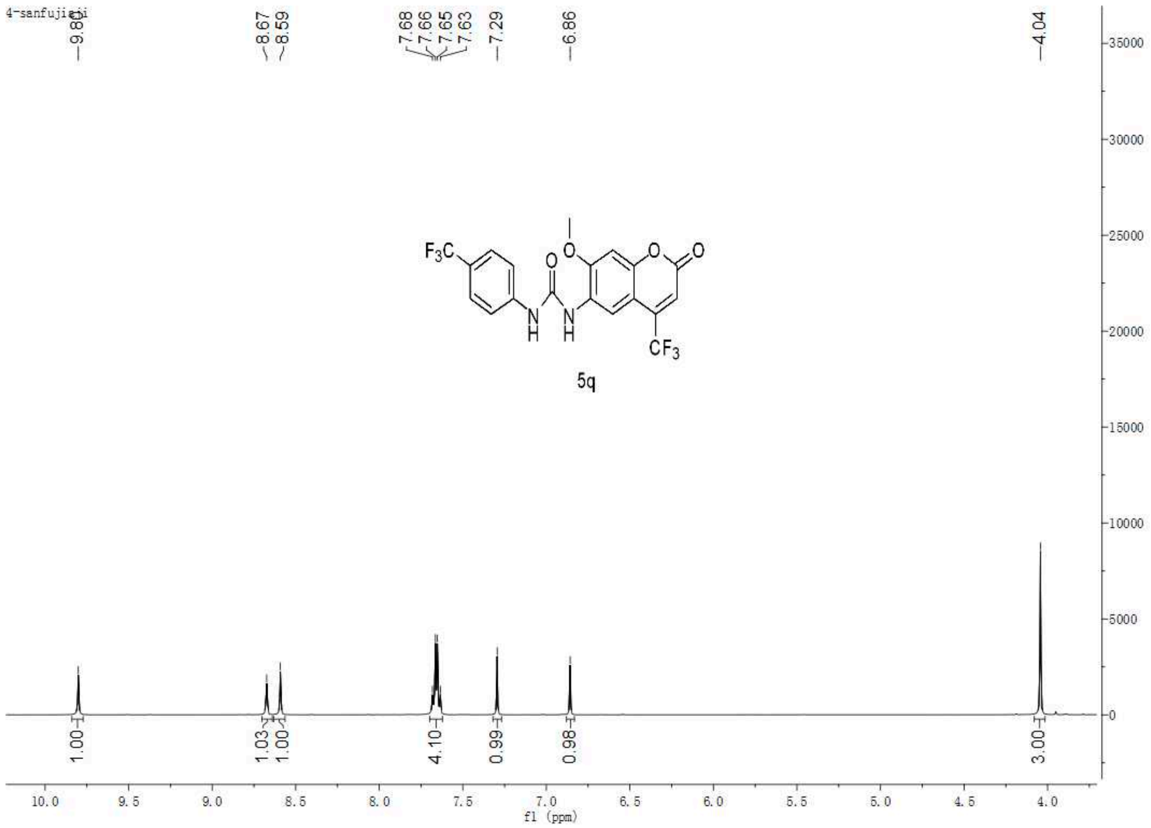

Figure S21. ^{1}H NMR of 5q

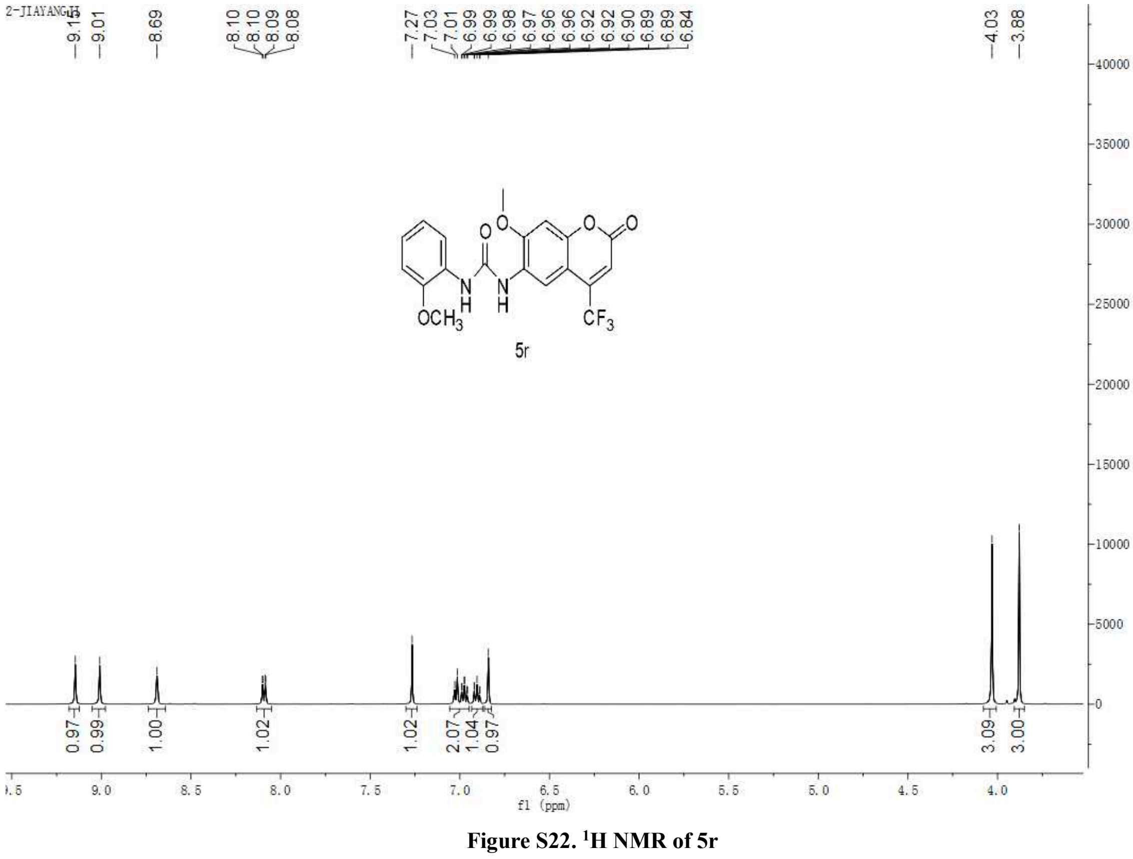

Figure S22. ^{1}H NMR of 5r

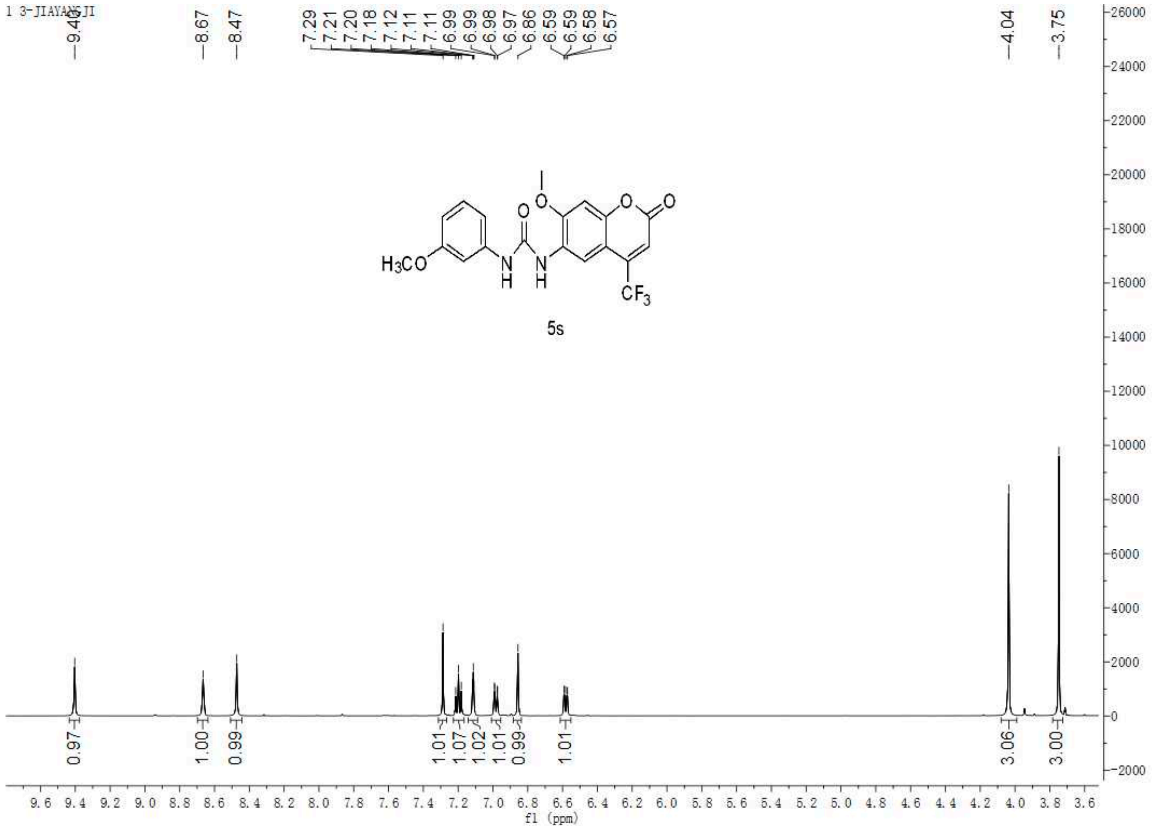

Figure S23. ¹H NMR of 5s

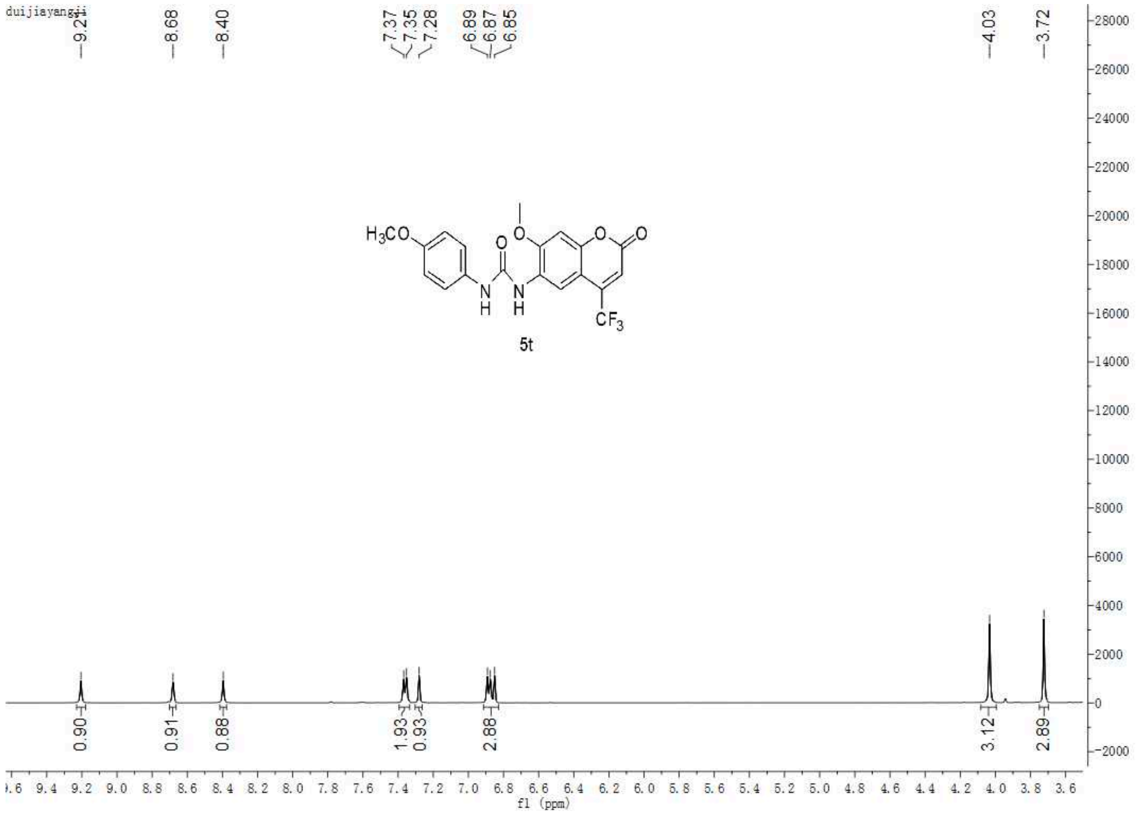

Figure S24. ¹H NMR of 5t

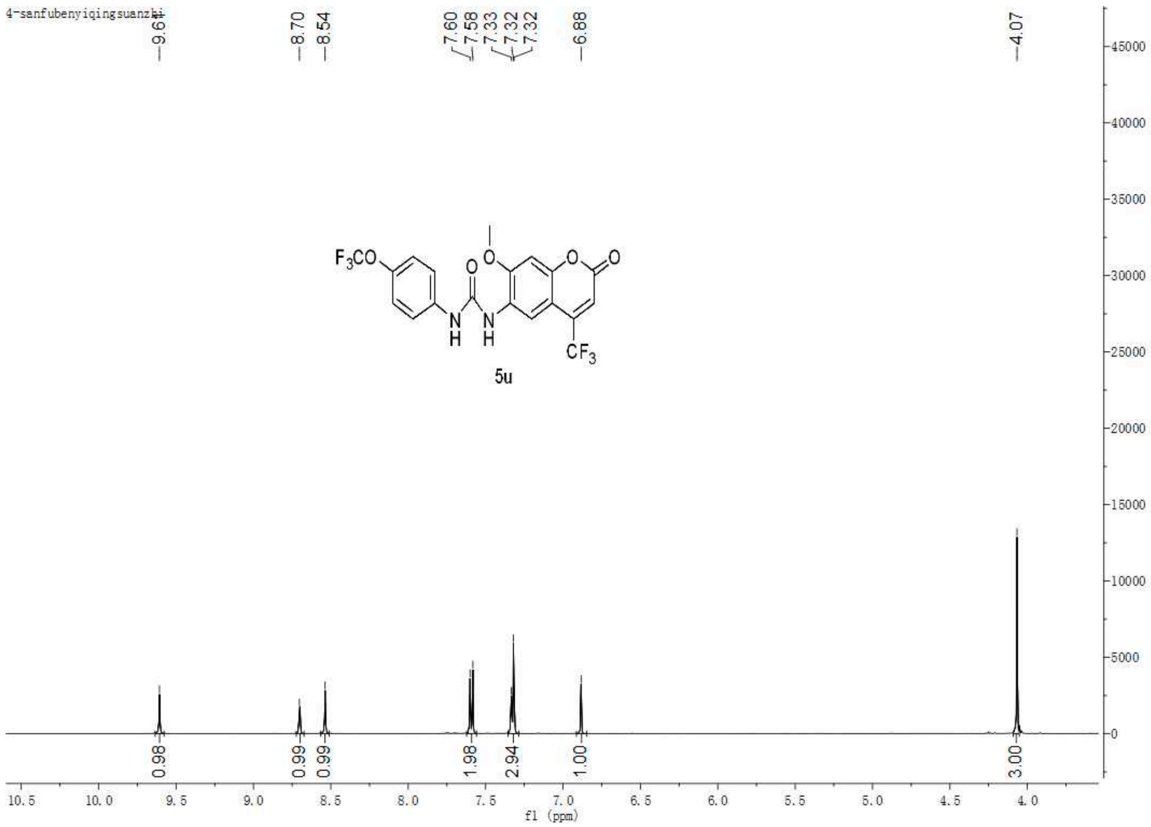

Figure S25. ^{1}**H NMR of 5u**

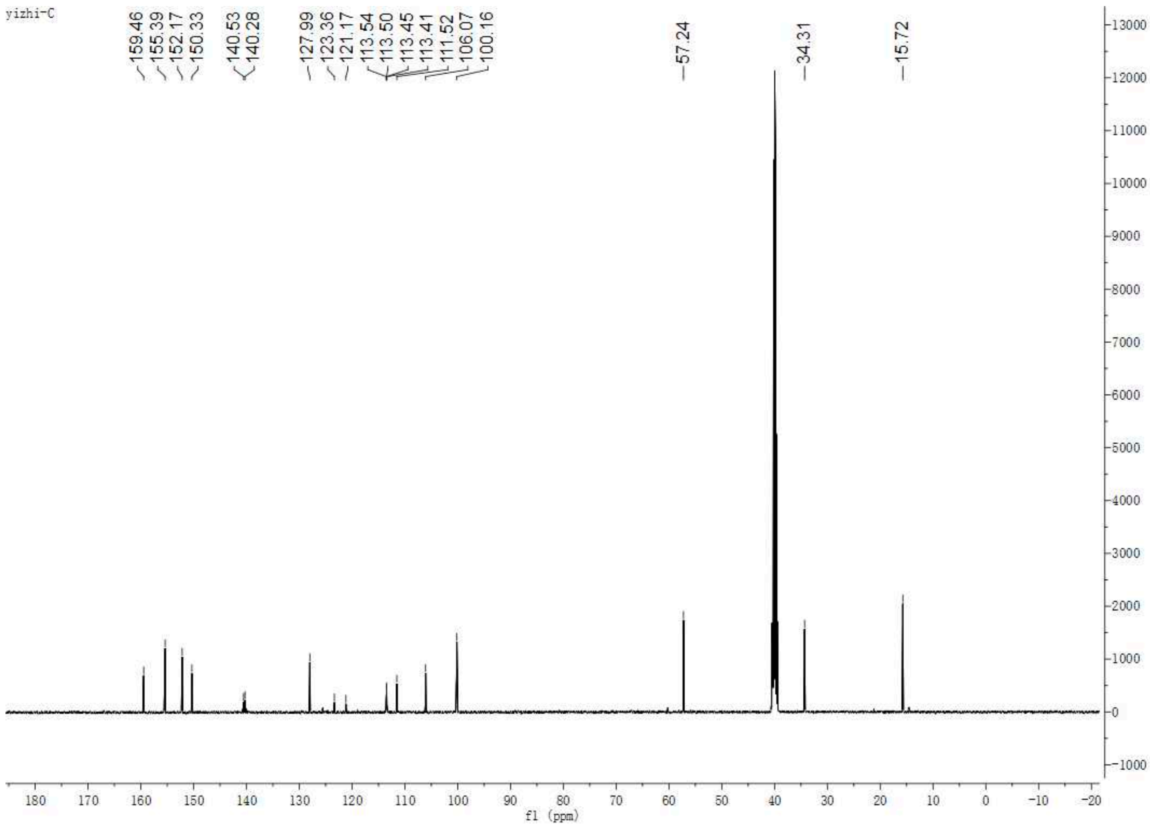

Figure S26. ^{13}C NMR of 5a

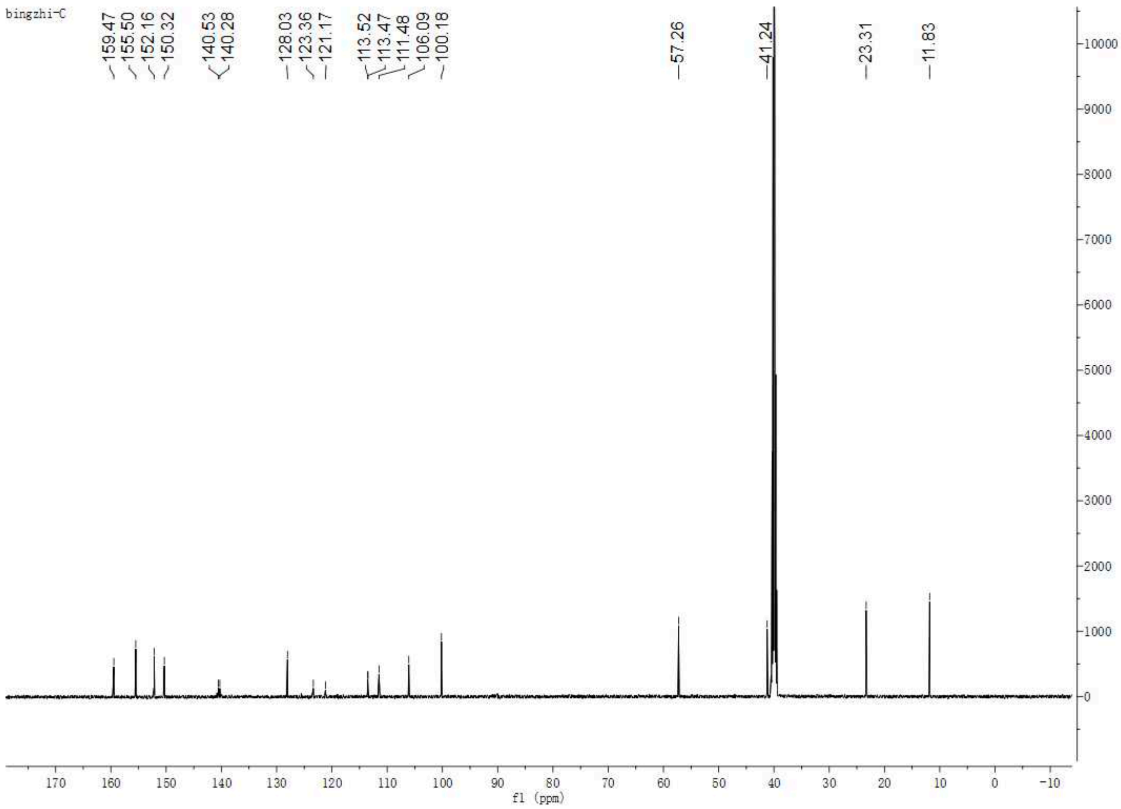

Figure S27. ^{13}C NMR of 5b

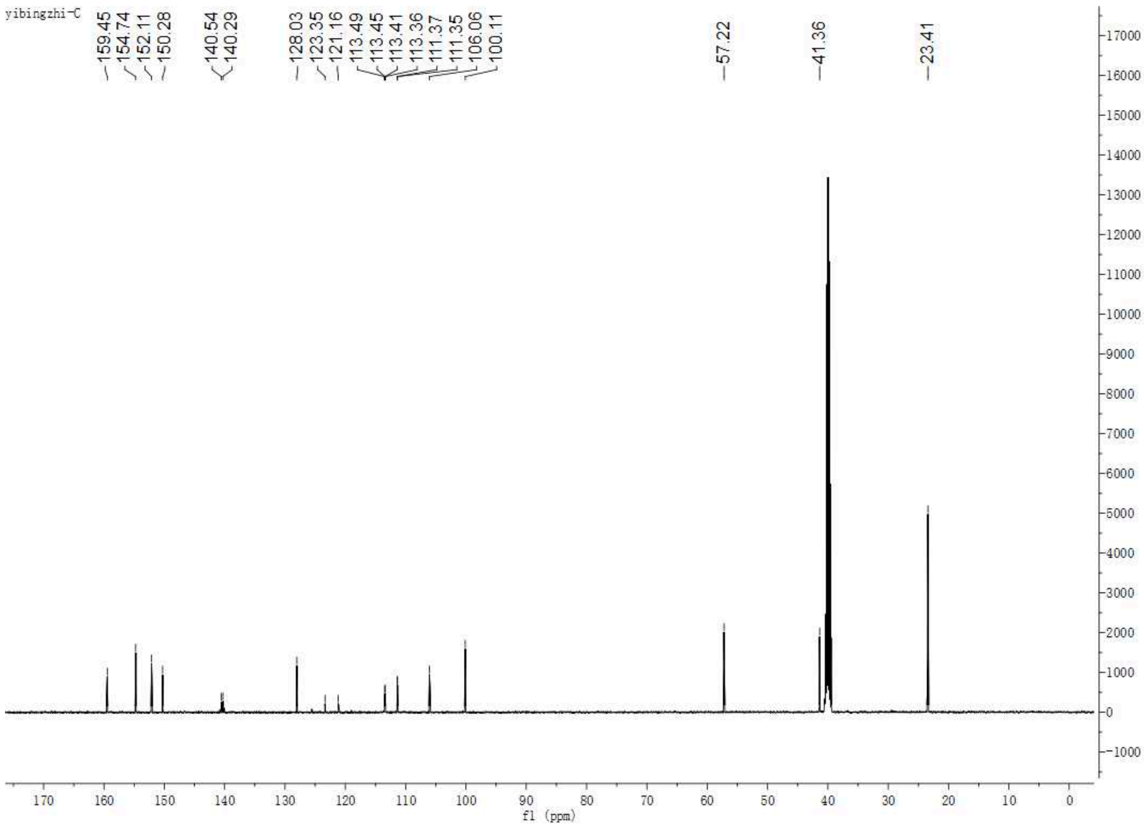

Figure S28. ^{13}C NMR of 5c

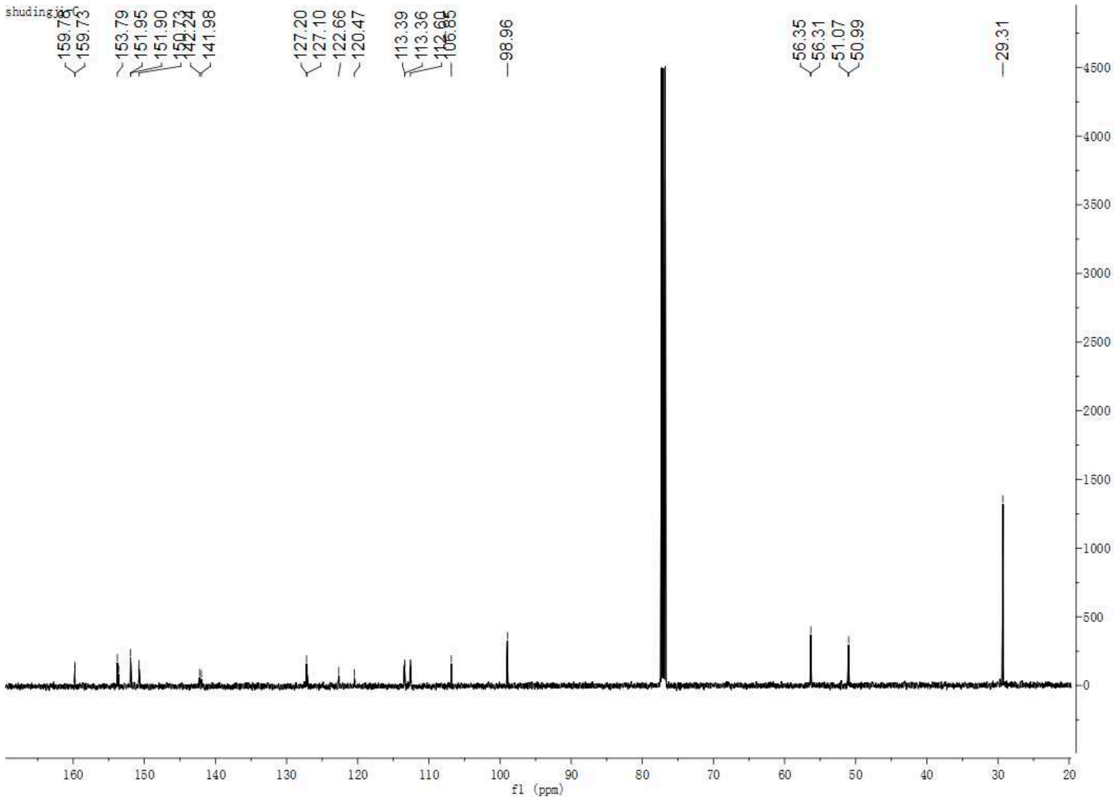

Figure S29. ^{13}C NMR of 5d

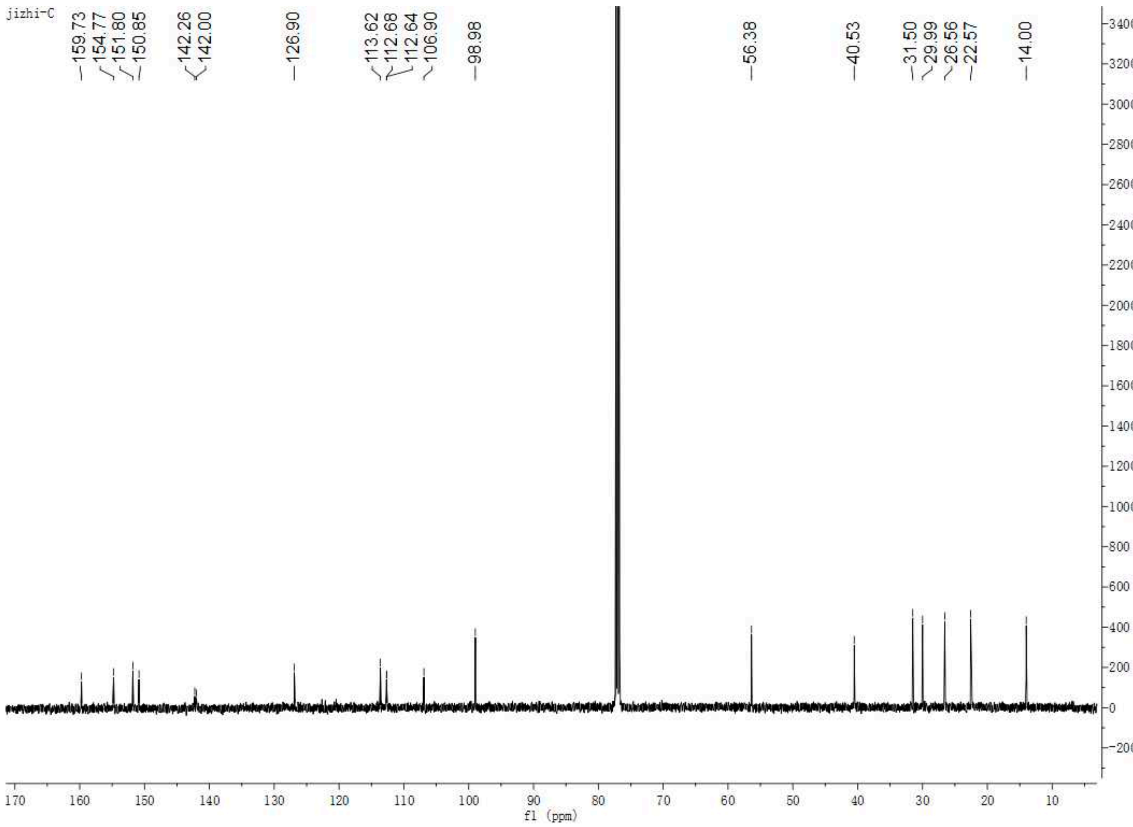

Figure S30. ^{13}C NMR of **5e**

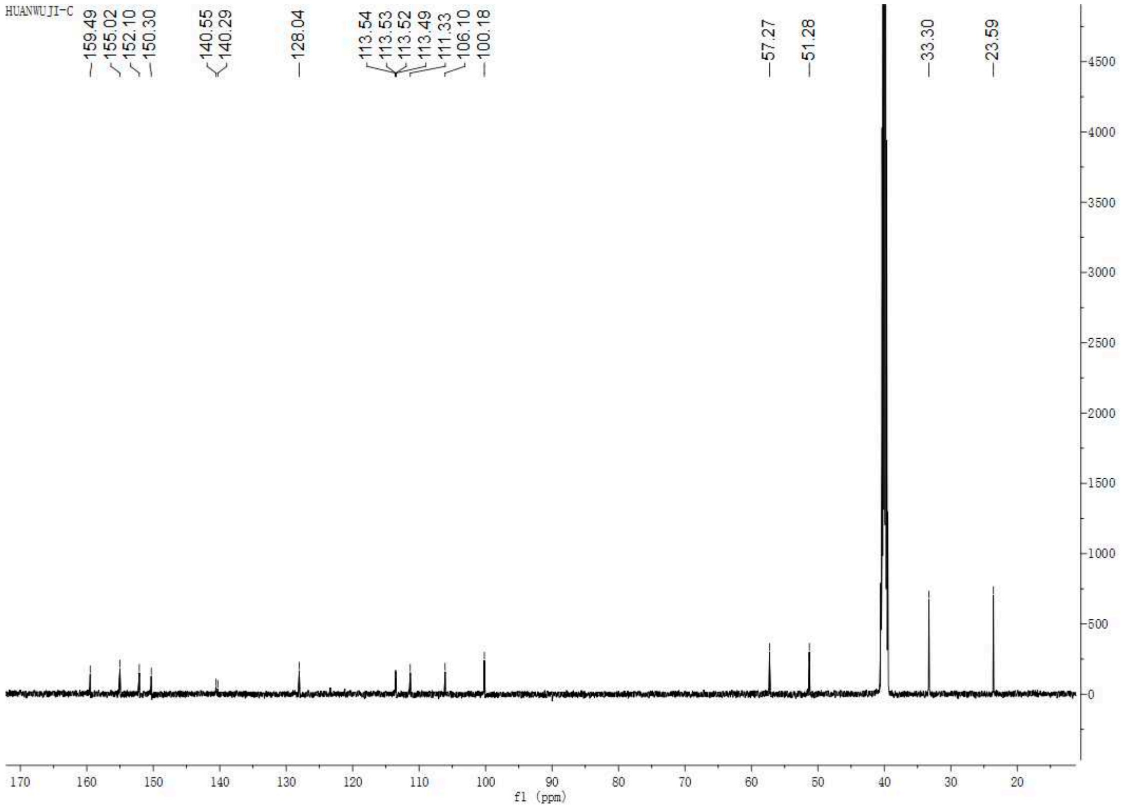

Figure S31. ^{13}C NMR of **5f**

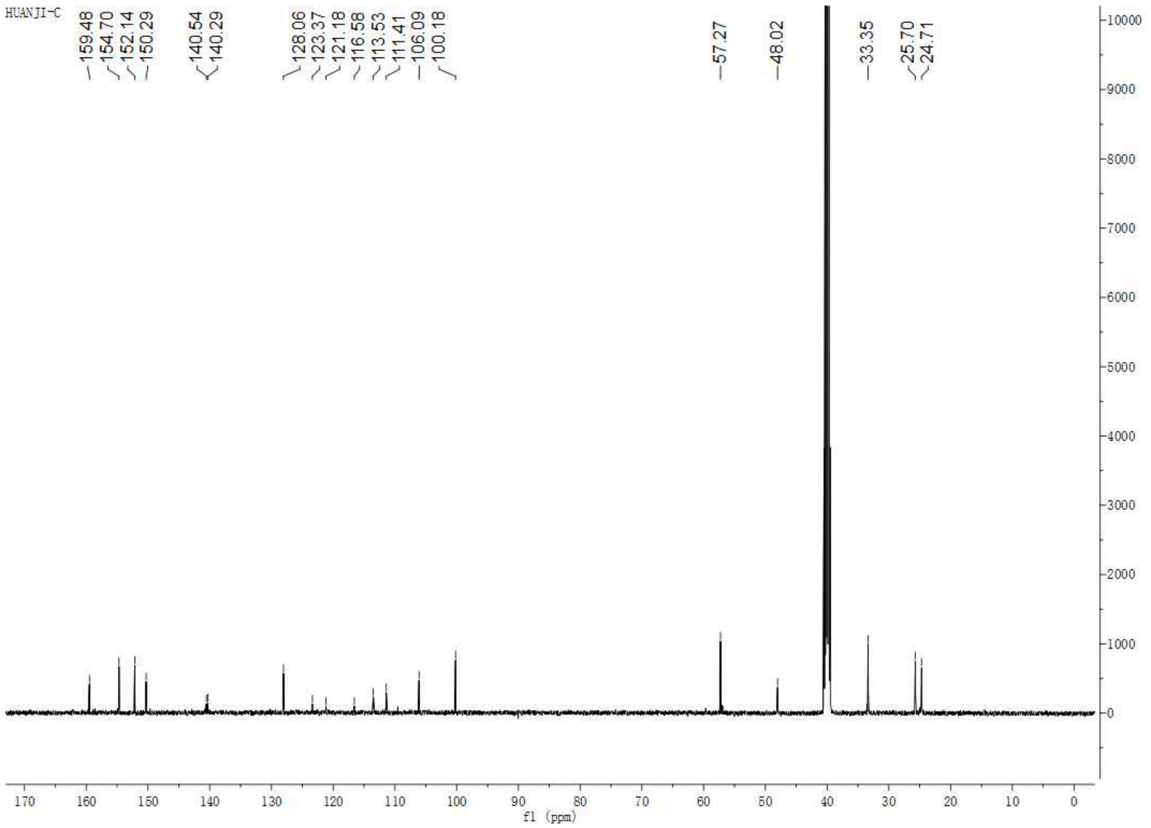

Figure S32. ^{13}**CNMR of 5g**

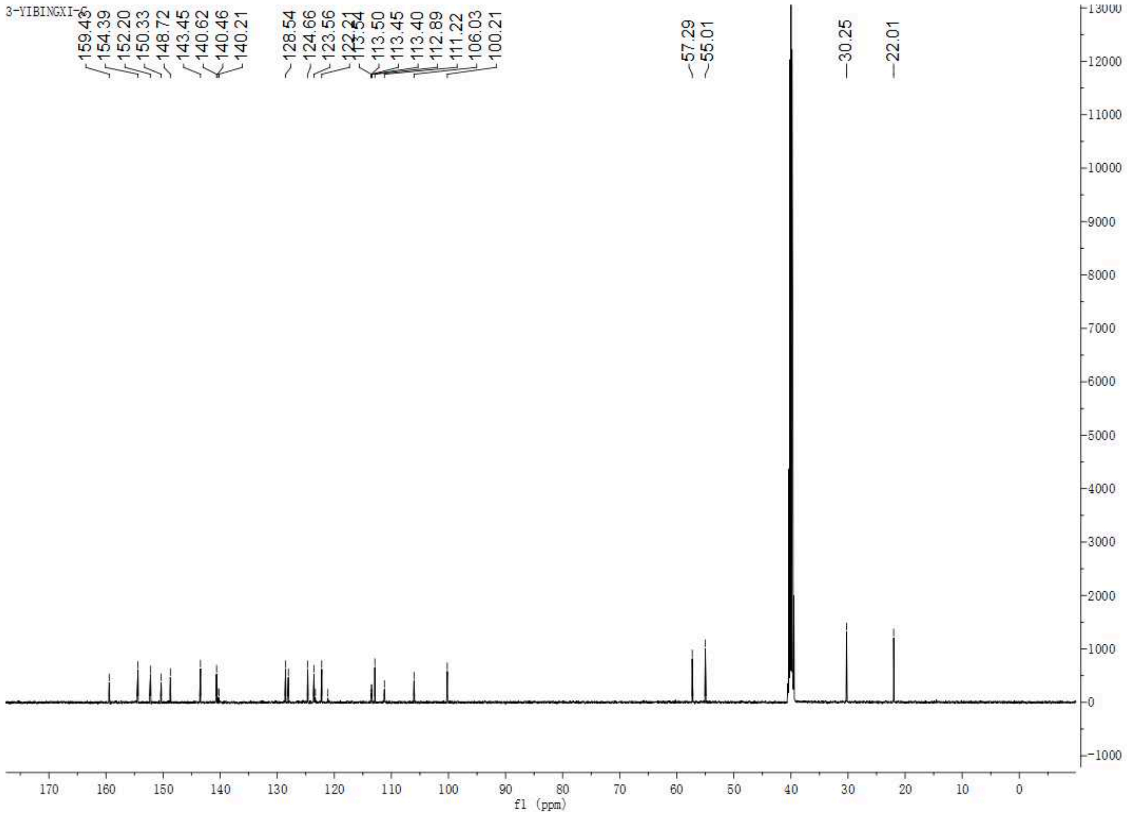

Figure S33. ^{13}**C NMR of 5h**

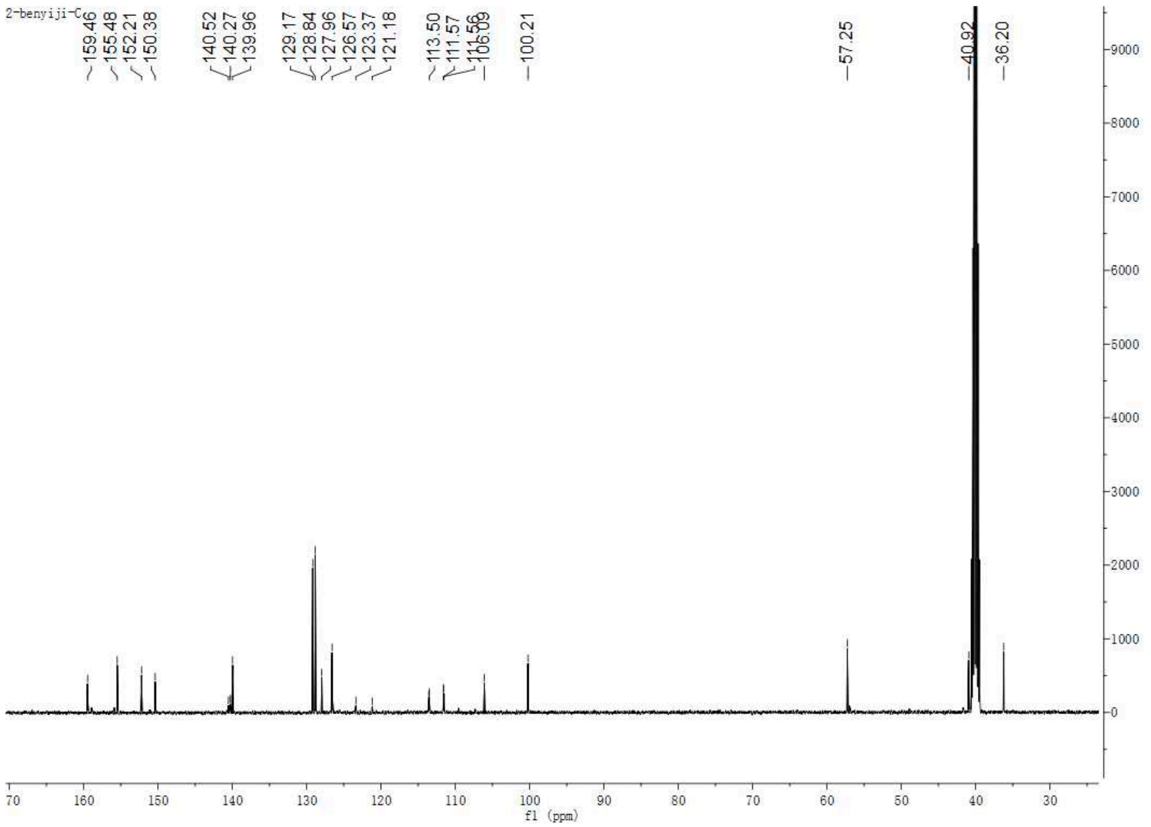

Figure S34. ^{13}C **NMR of 5i**

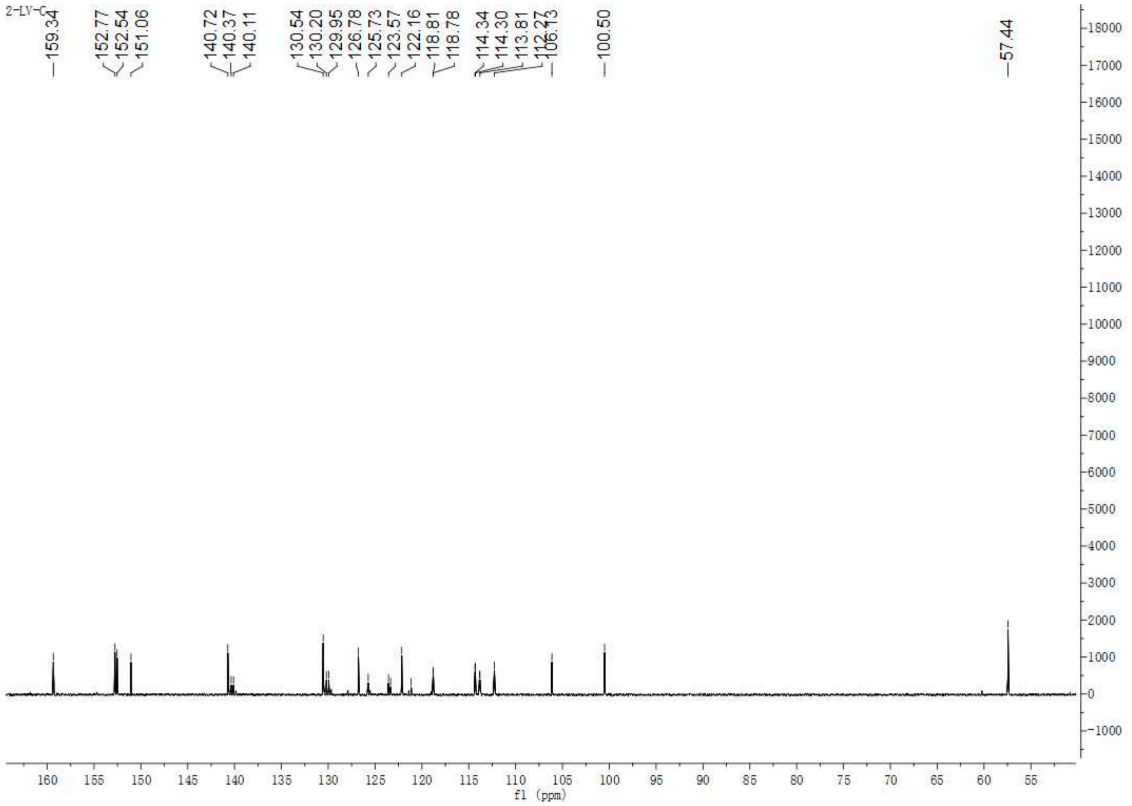

Figure S35. ^{13}C **NMR of 5j**

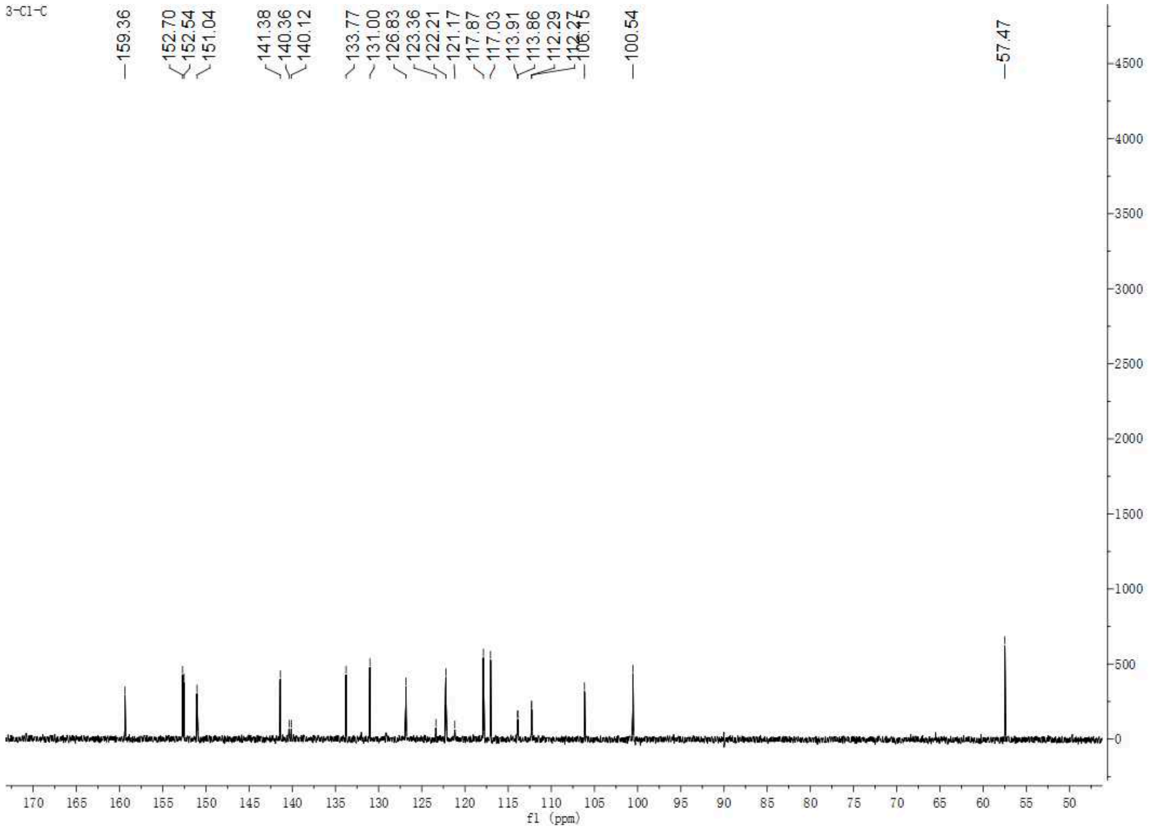

Figure S36. ^{13}C NMR of 5k

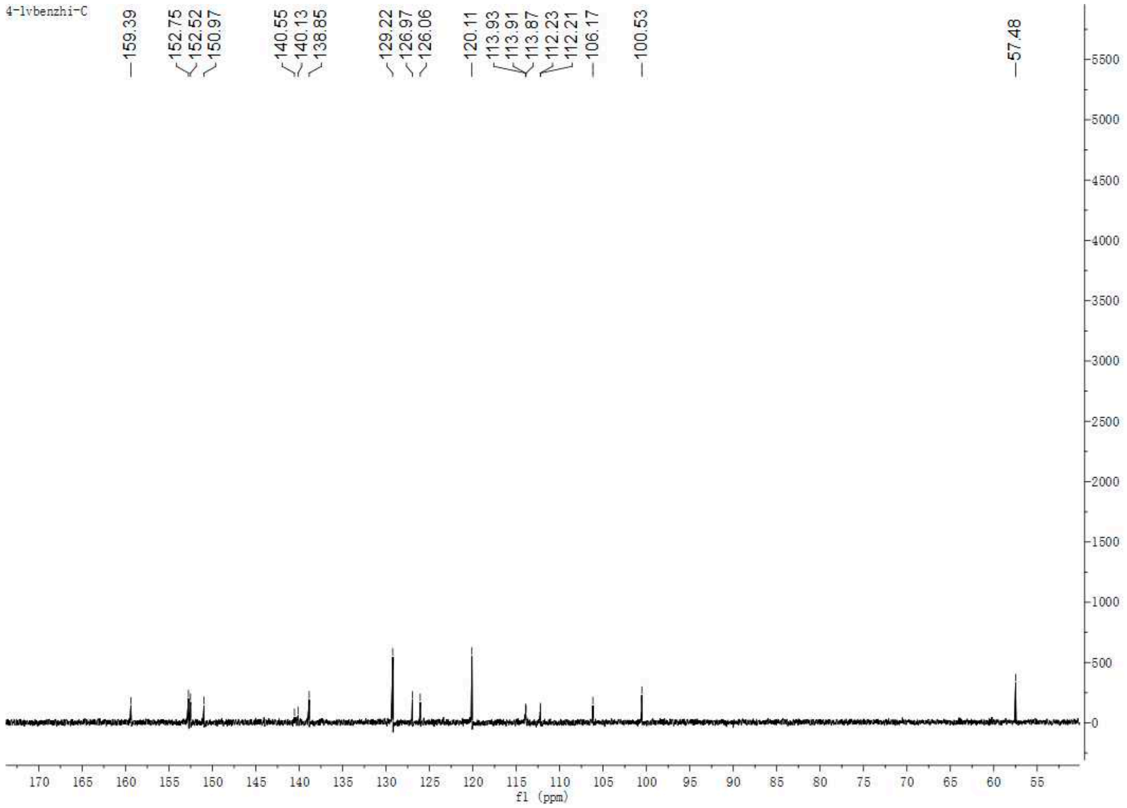

Figure S37. ^{13}C NMR of 5l

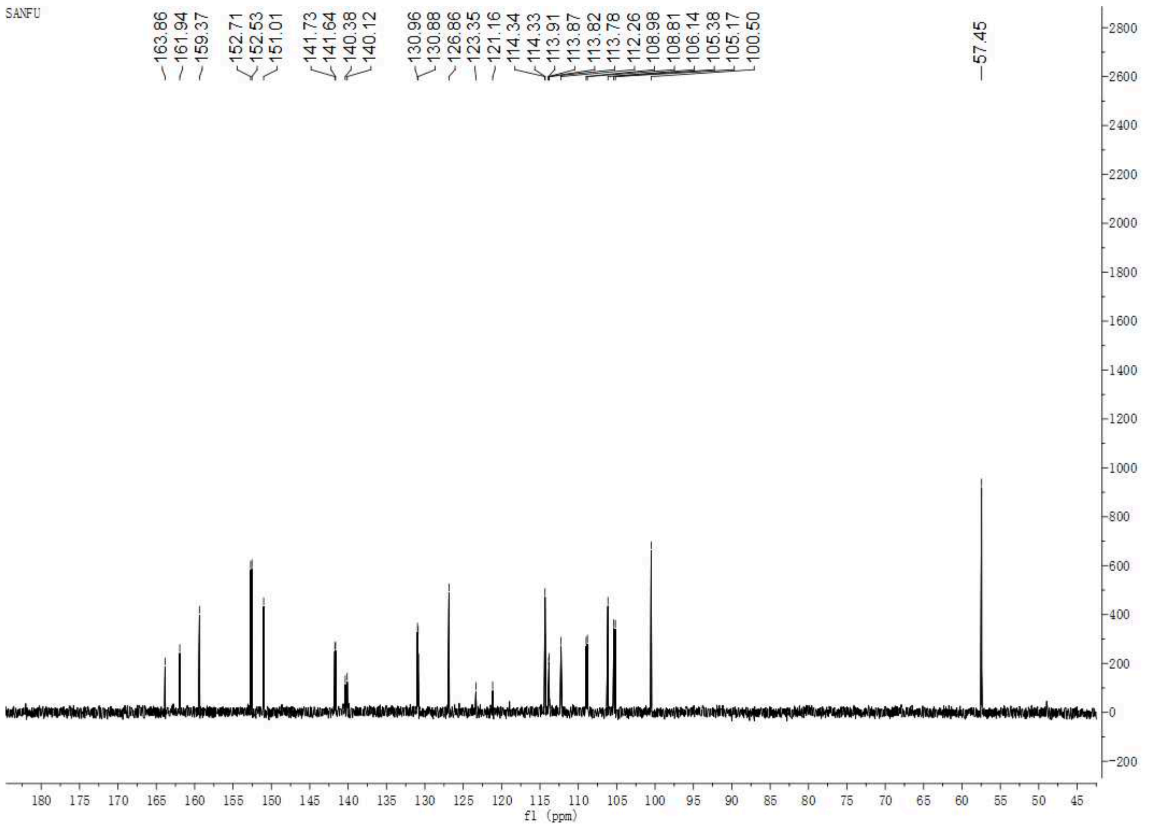

Figure S38. ^{13}C NMR of 5m

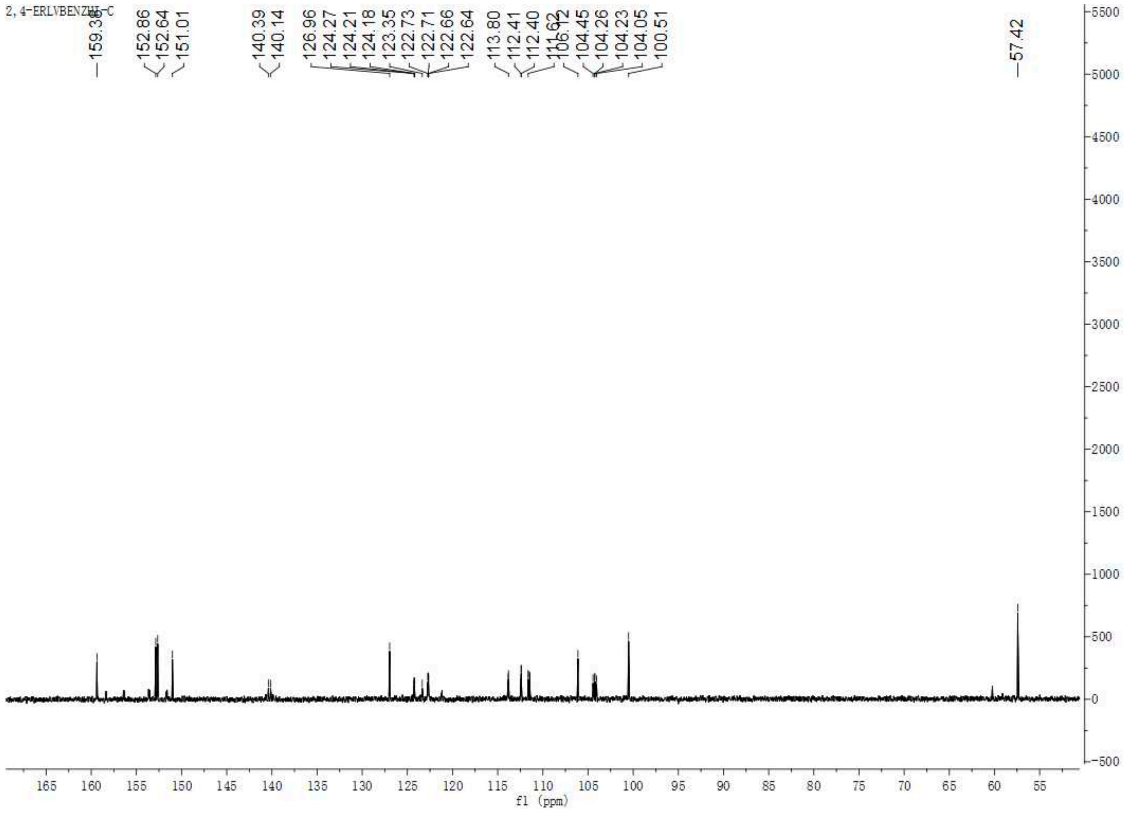

Figure S39. ^{13}C NMR of 5n

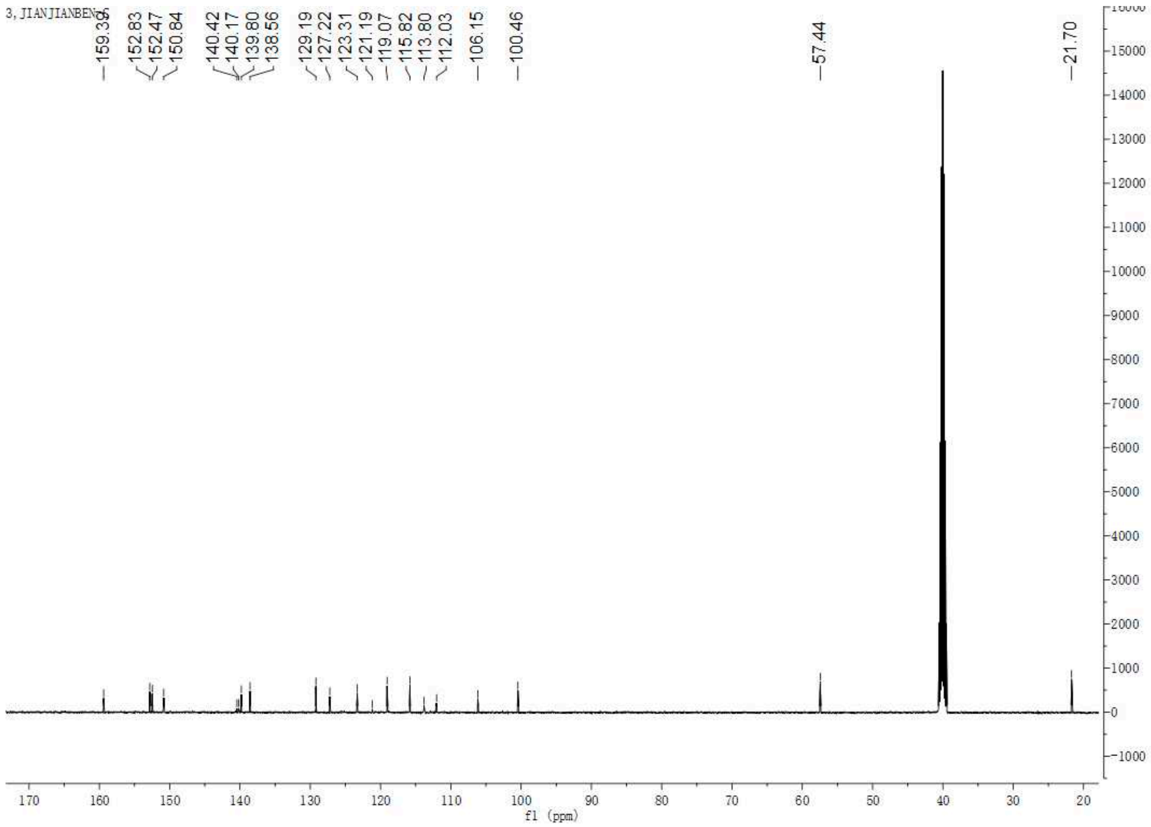

Figure S40. ^{13}**C NMR of 5o**

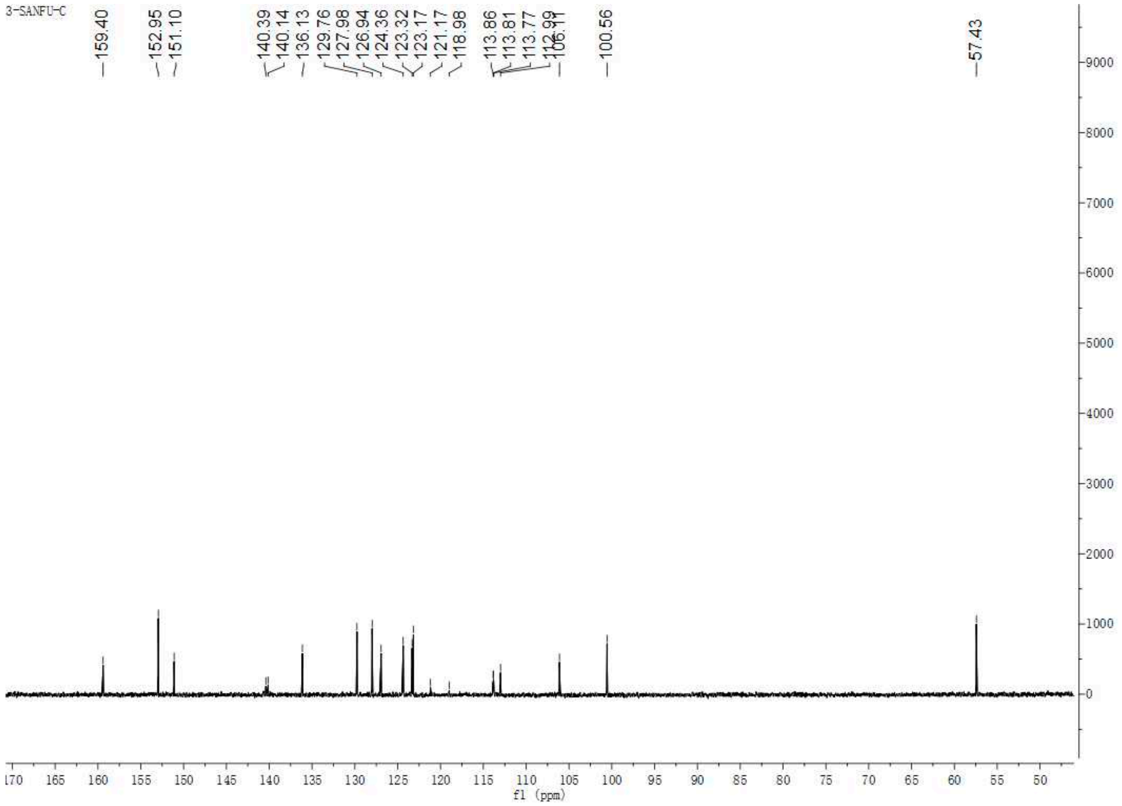

Figure S41. ^{13}**C NMR of 5p**

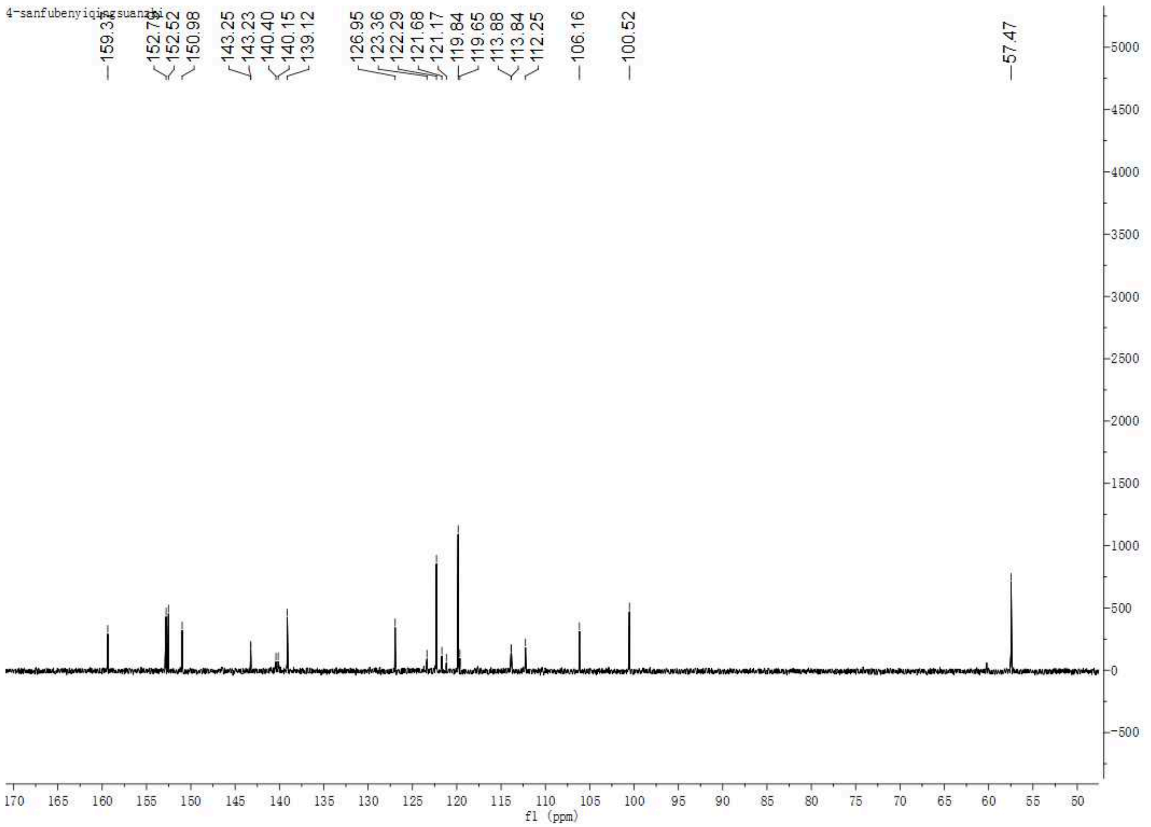

Figure S42. ^{13}**C NMR of 5q**

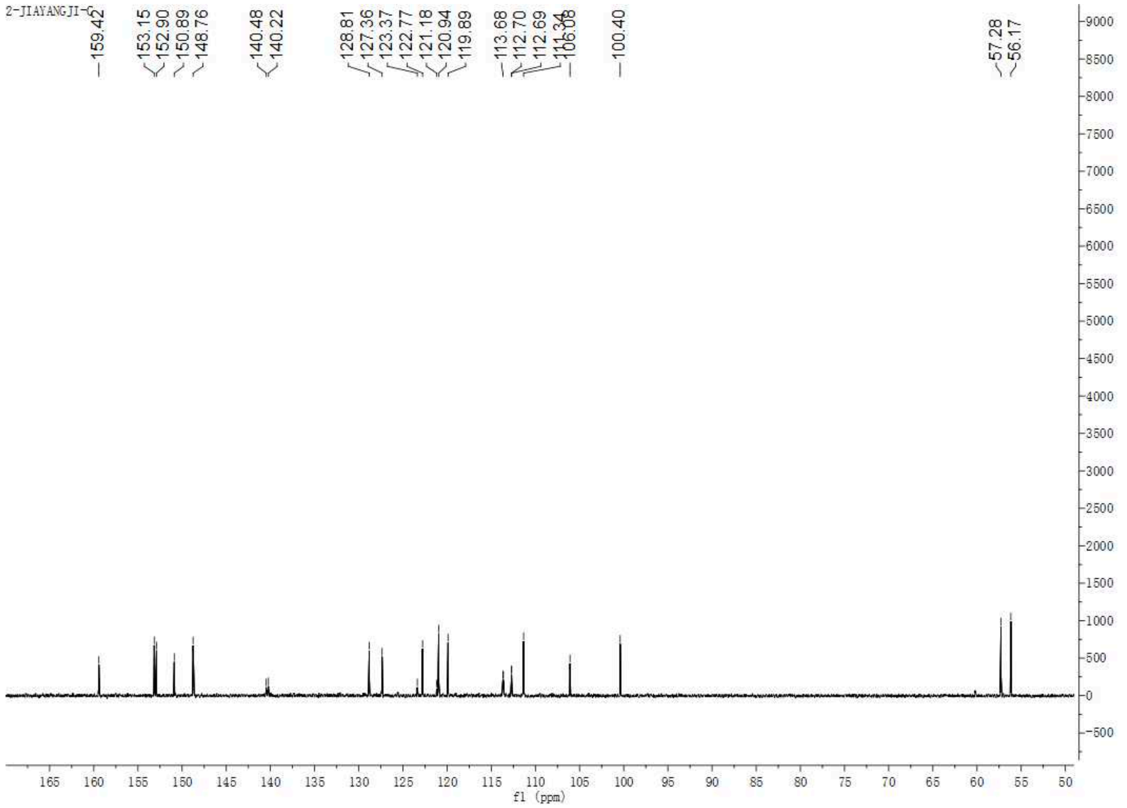

Figure S43. ^{13}**C NMR of 5r**

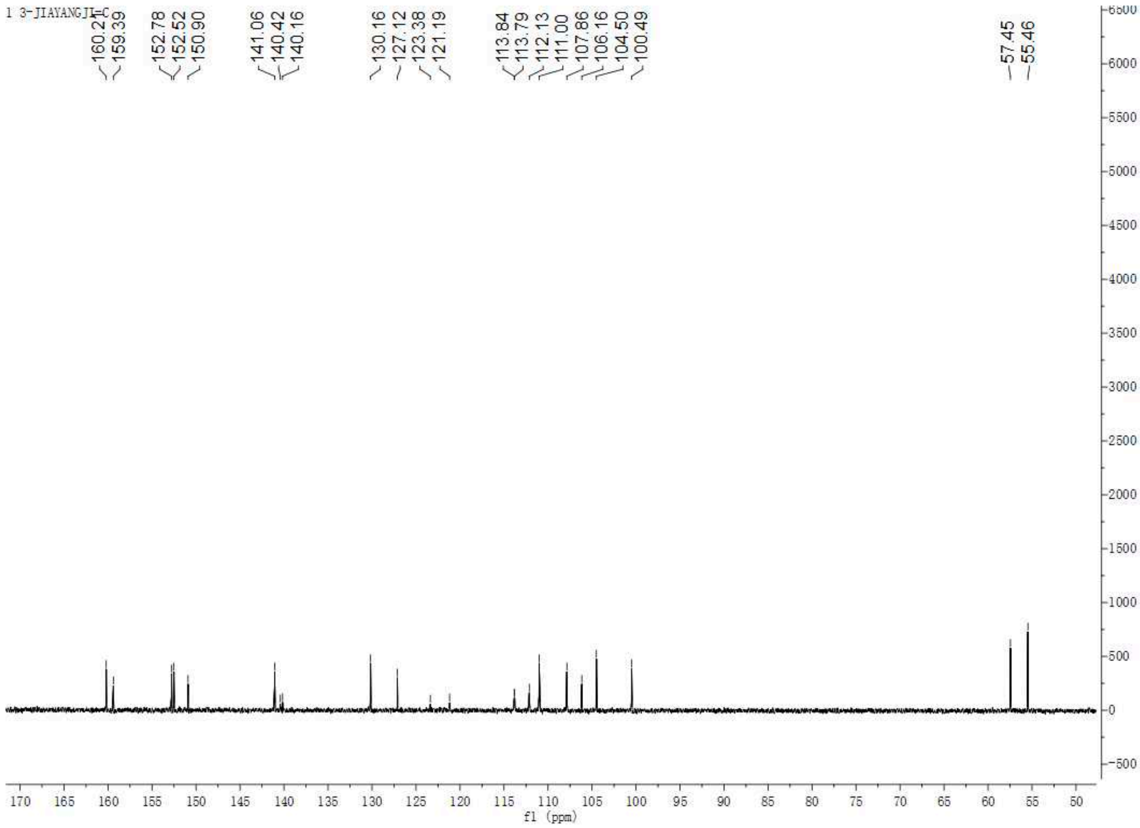

Figure S44. ^{13}C NMR of 5s

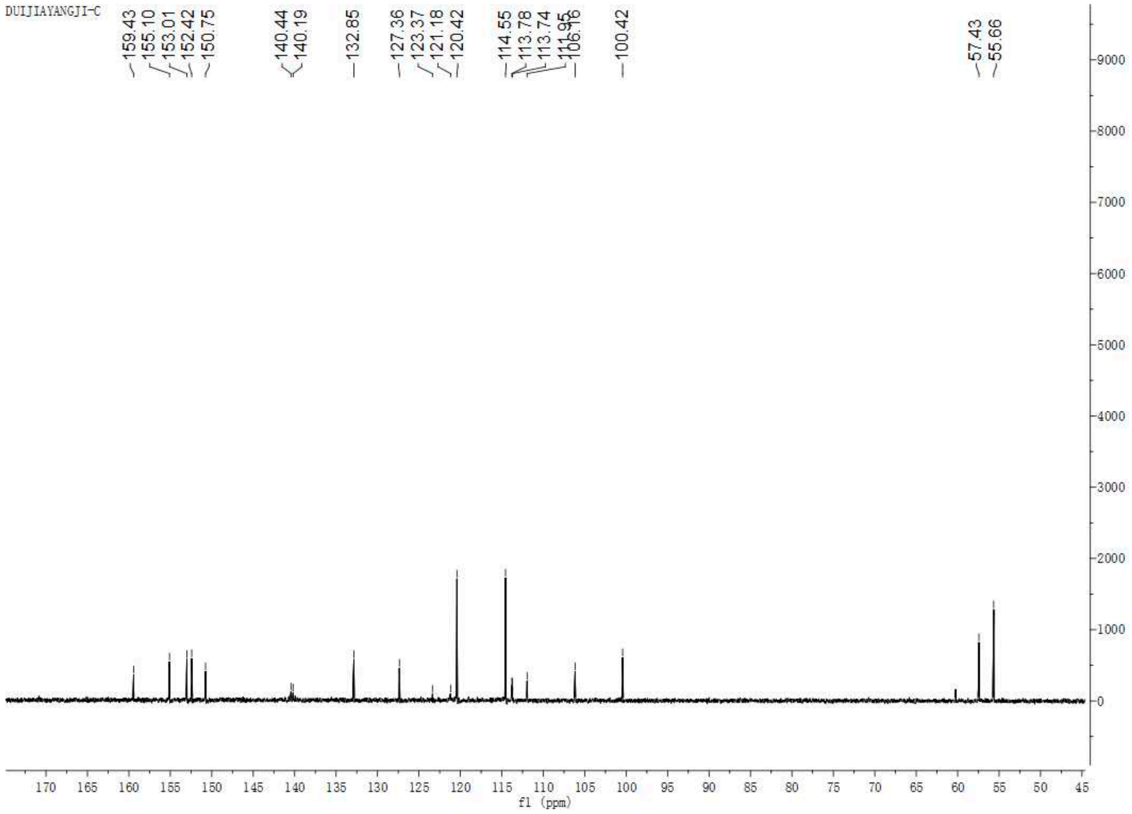

Figure S45. ^{13}C NMR of 5t

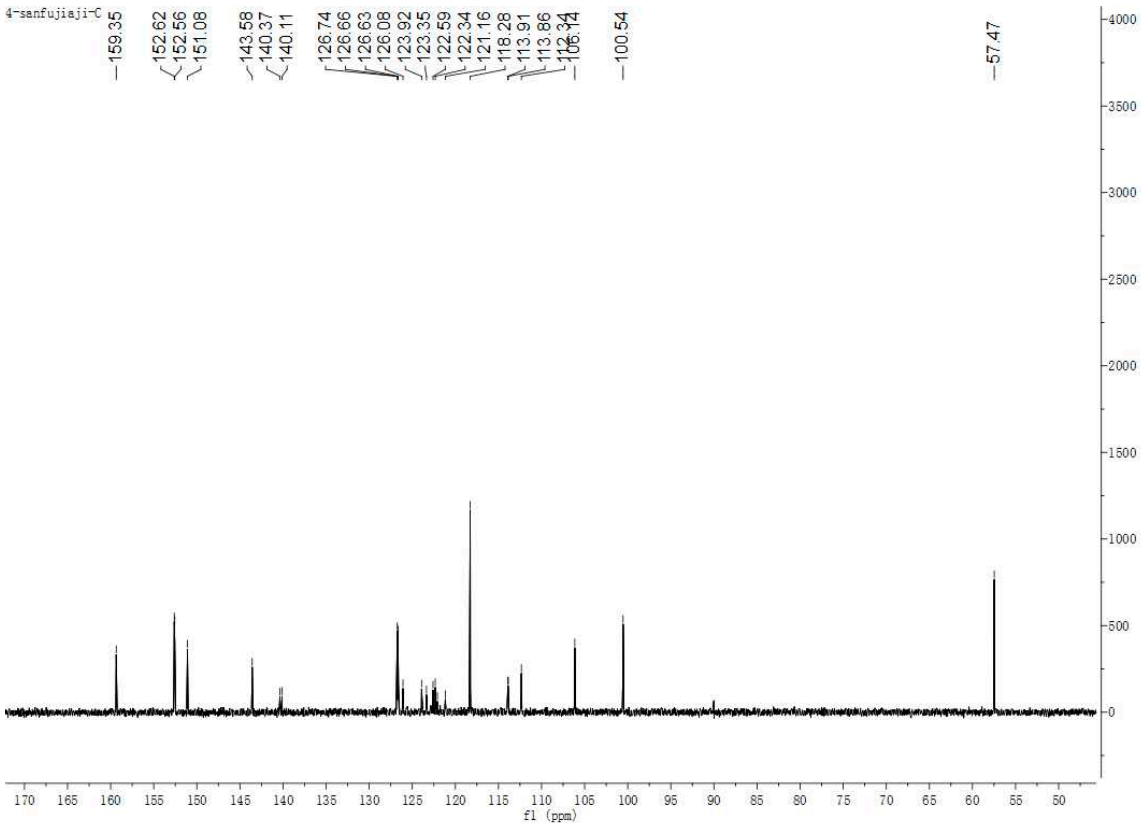

Figure S46. ^{13}C NMR of 5u

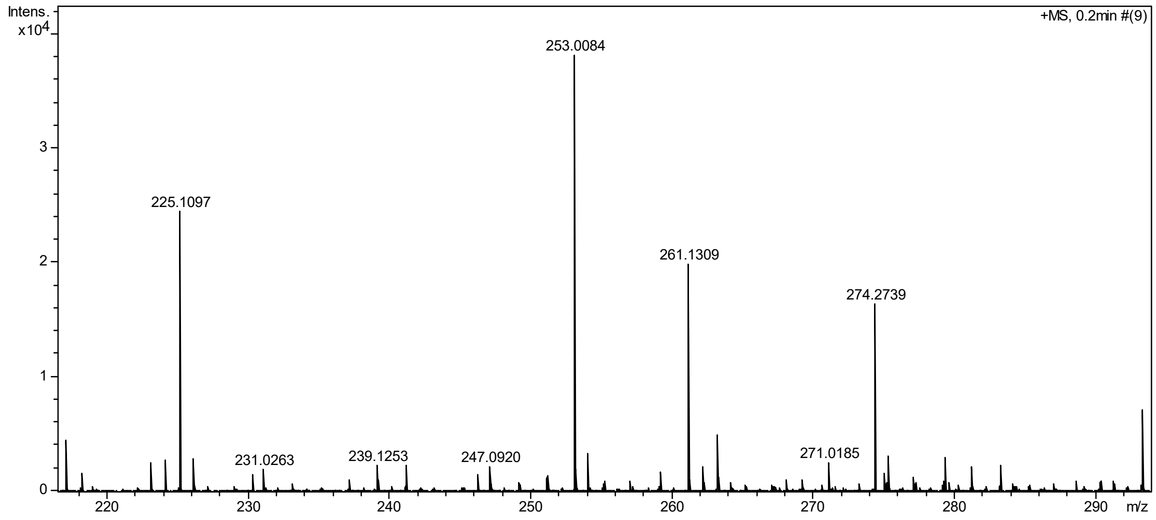

Figure S47. HRMS of 1

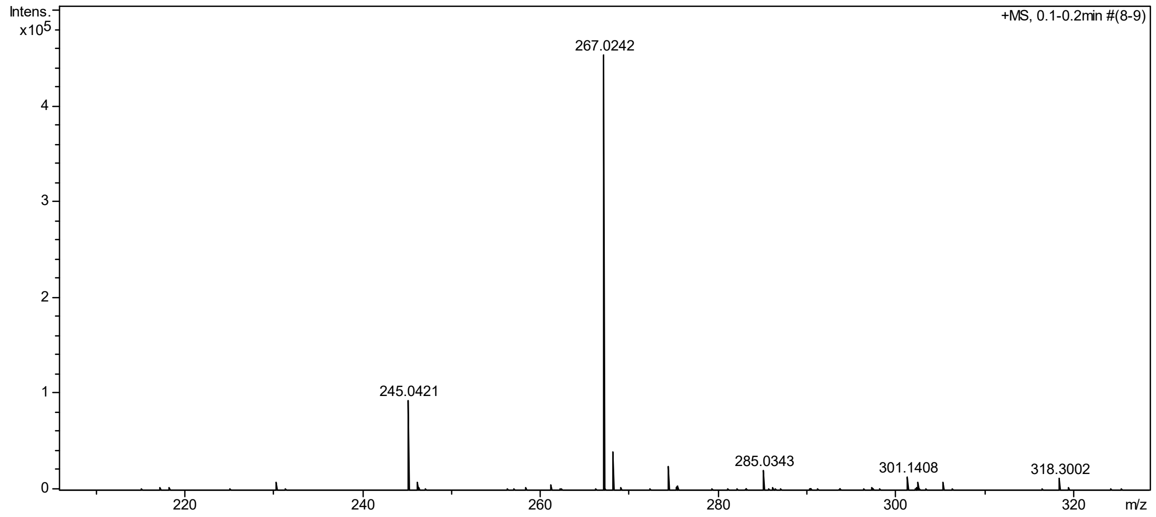

Figure S48. HRMS of 2

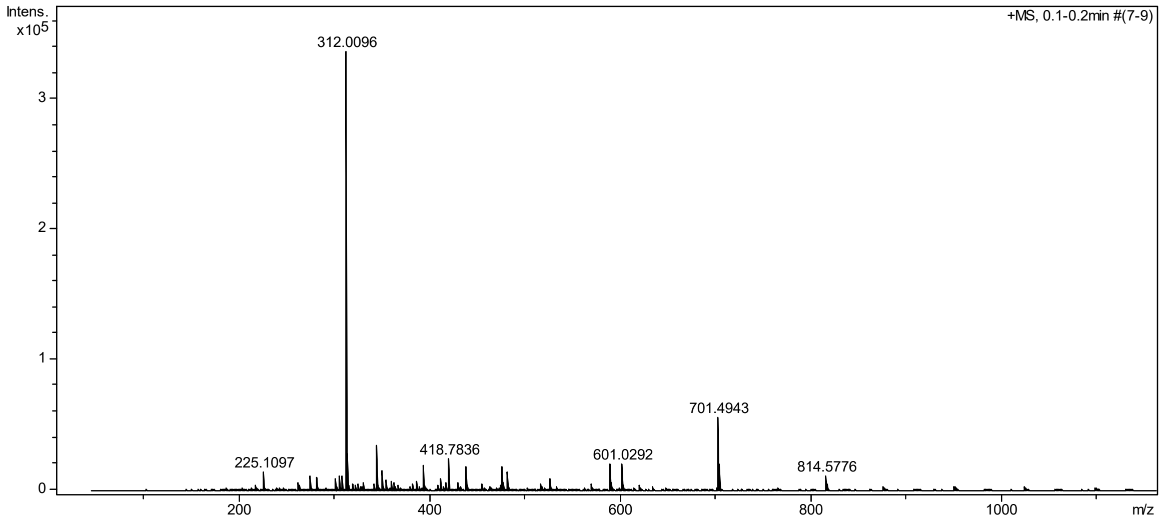

Figure S49. HRMS of 3

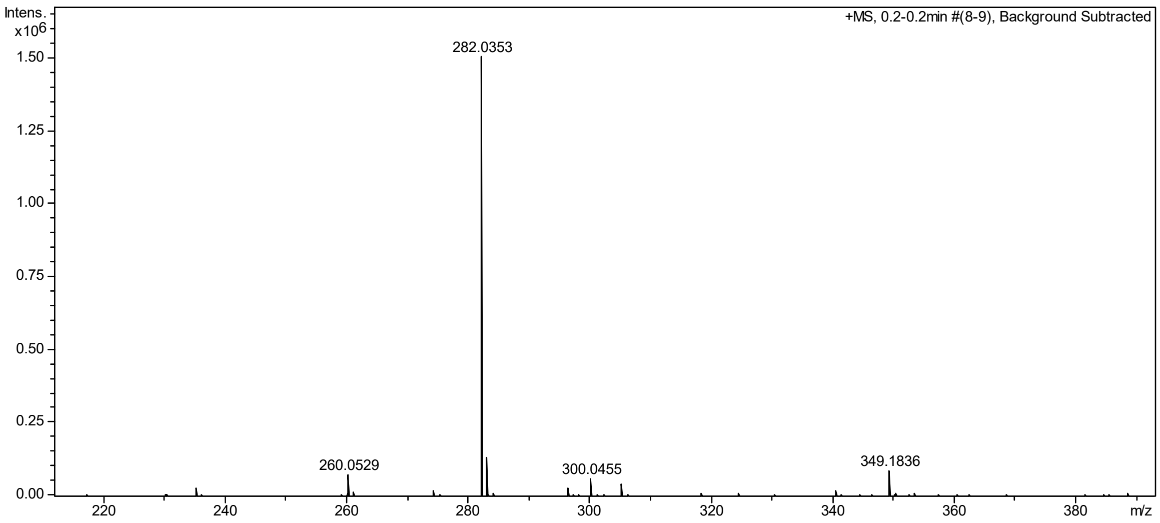

Figure S50. HRMS of 4

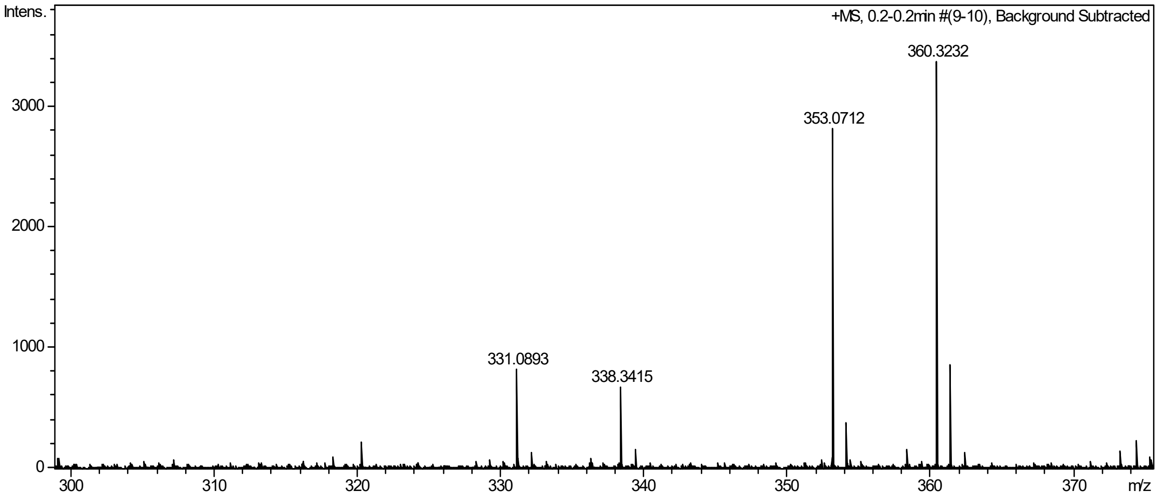

Figure S51. HRMS of 5a

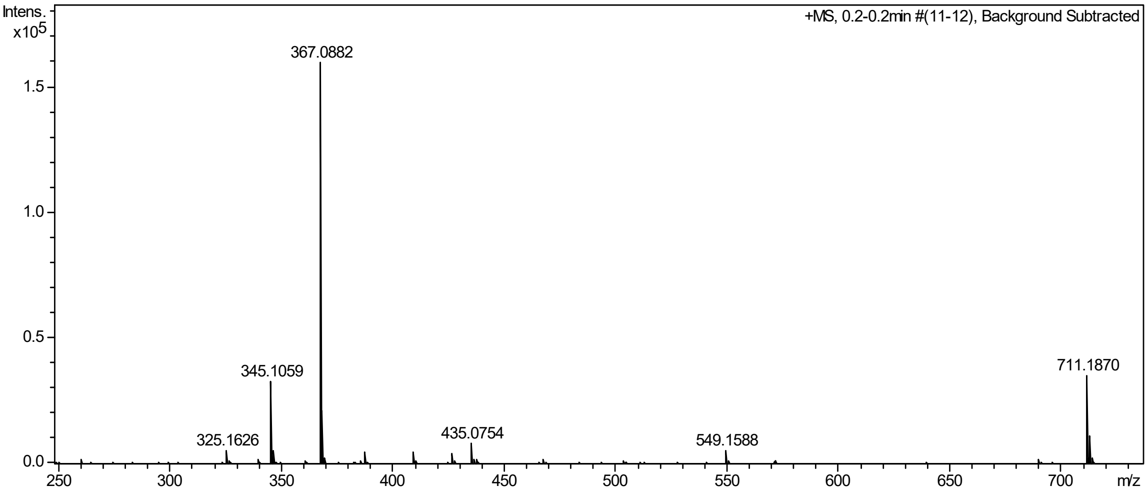

Figure S52. HRMS of 5b

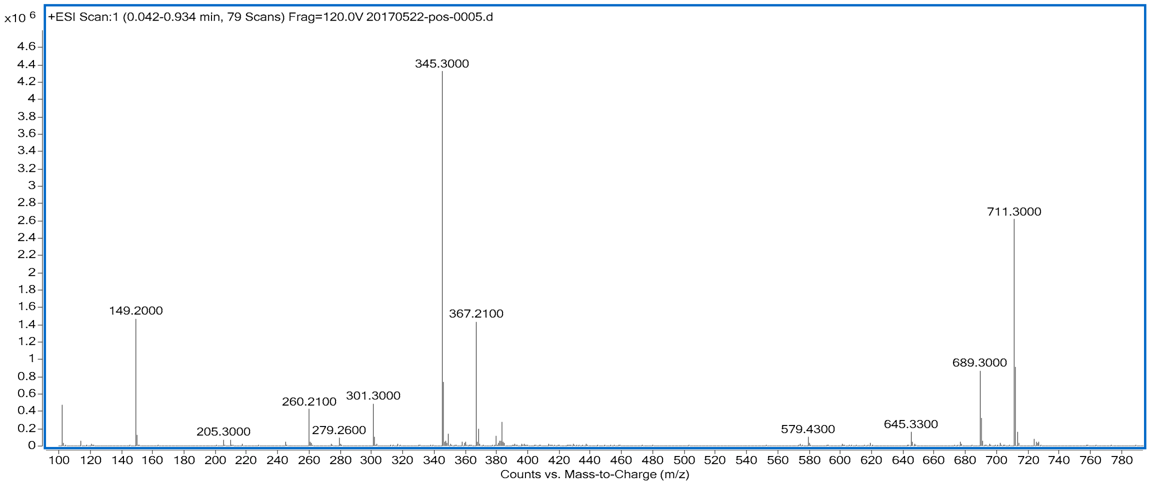

Figure S53. HRMS of 5c

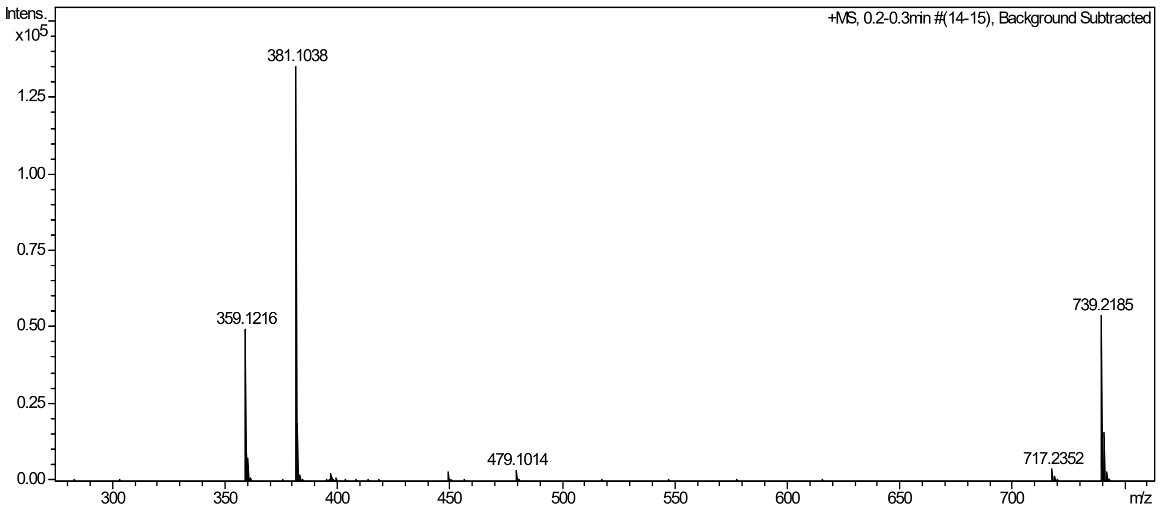

Figure S54. HRMS of 5d

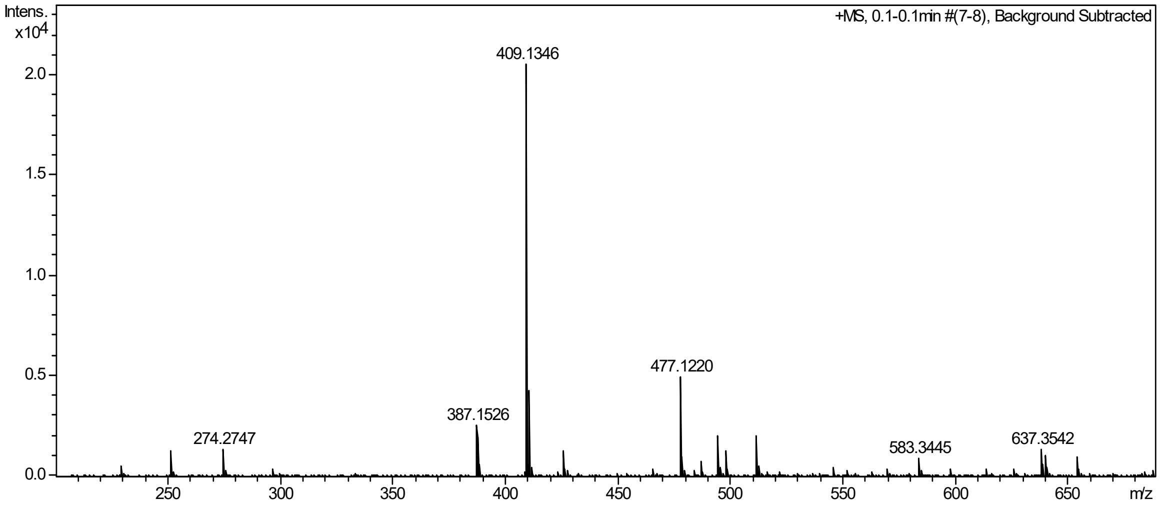

Figure S55. HRMS of 5e

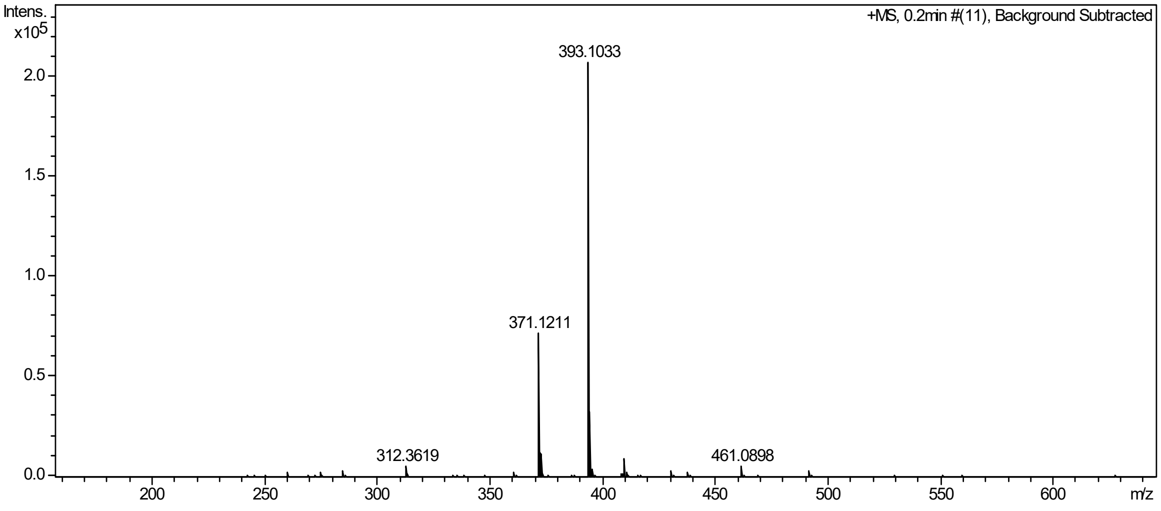

Figure S56 HRMS of 5f

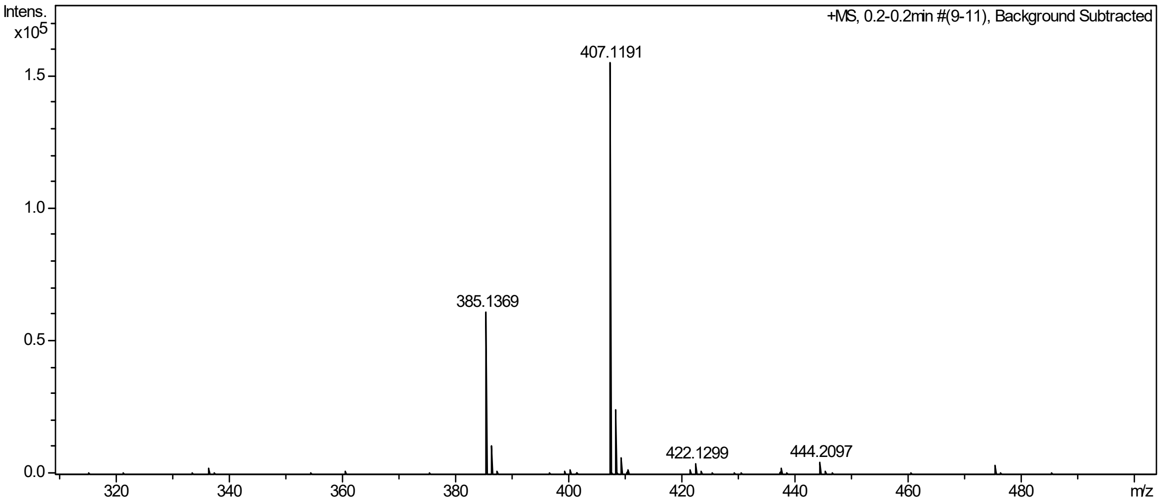

Figure S57. HRMS of 5g

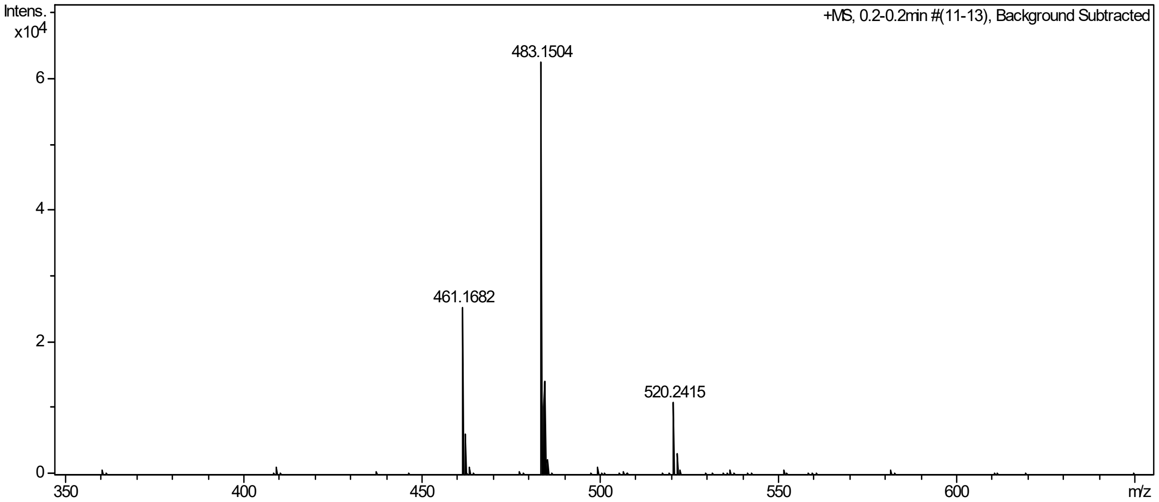

Figure S58. HRMS of 5h

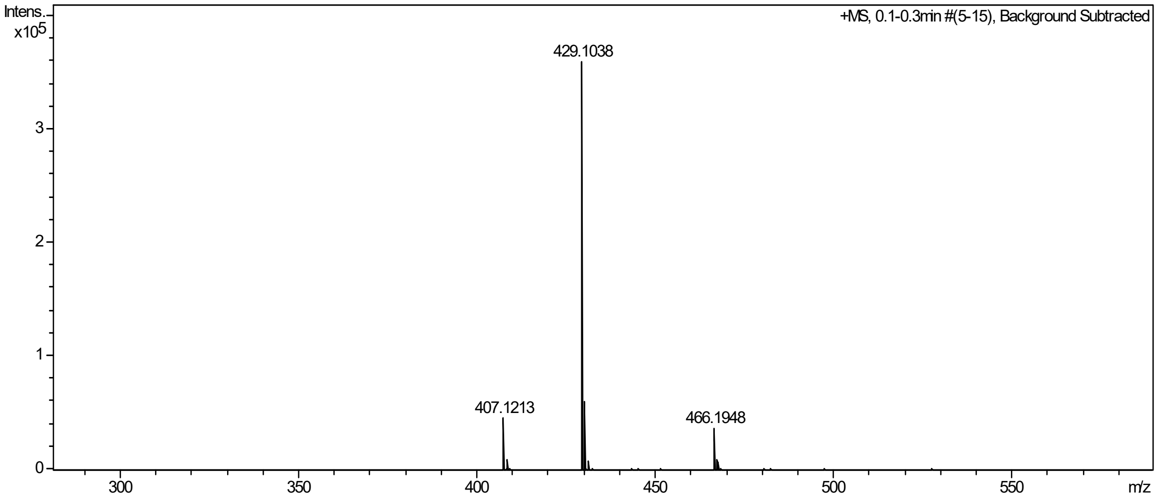

Figure S59. HRMS of 5i

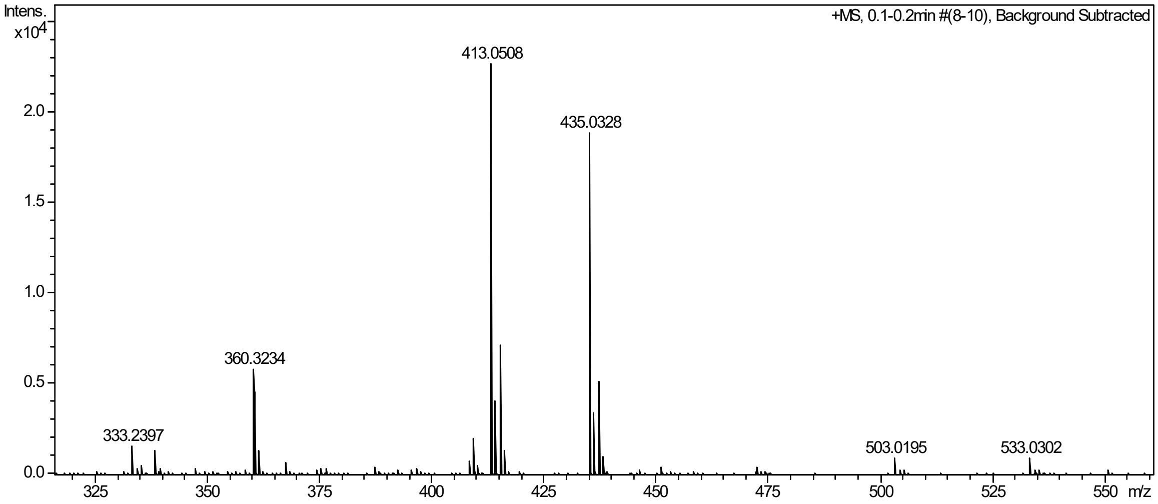

Figure S60. HRMS of 5j

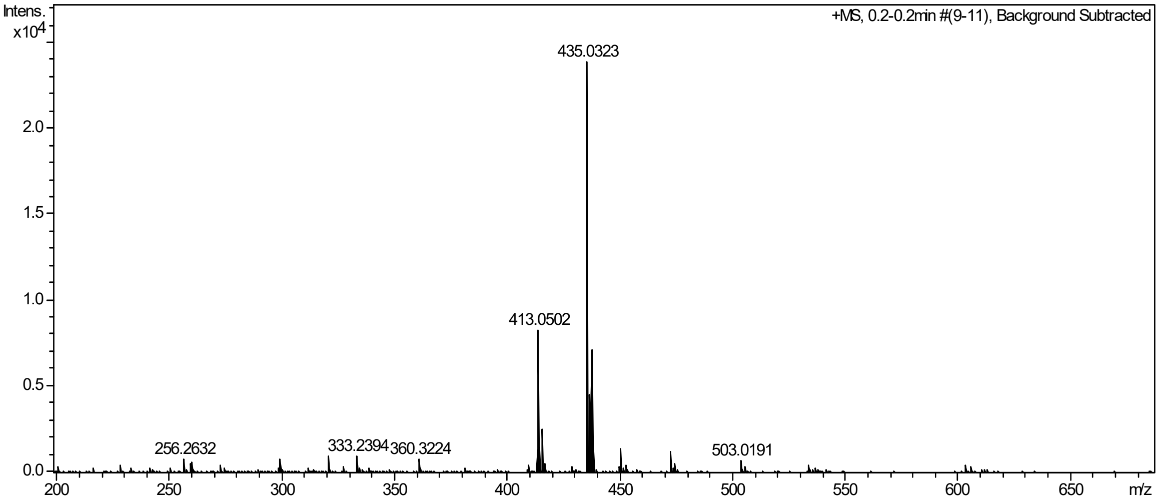

Figure S61. HRMS of 5k

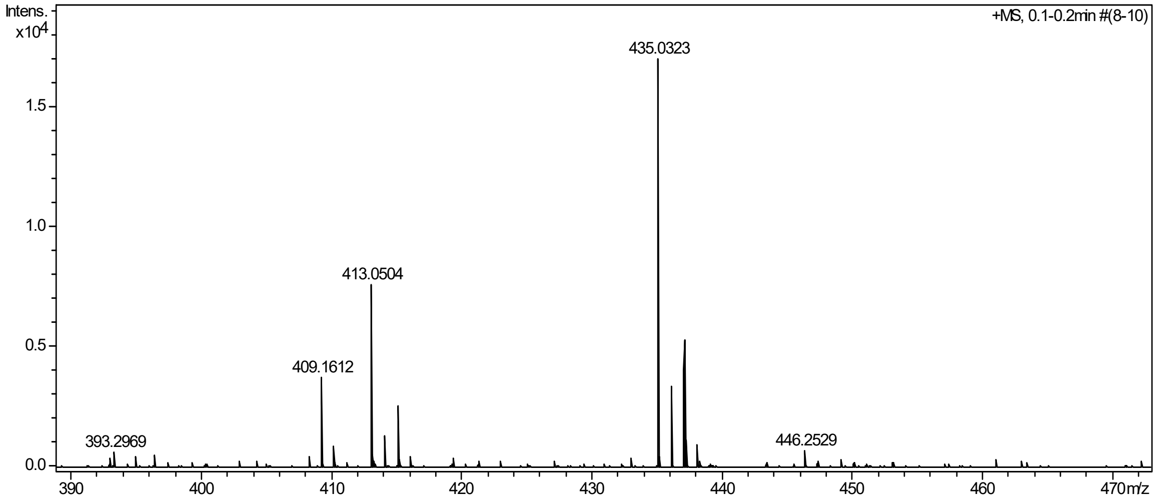

Figure S62 HRMS of 5l

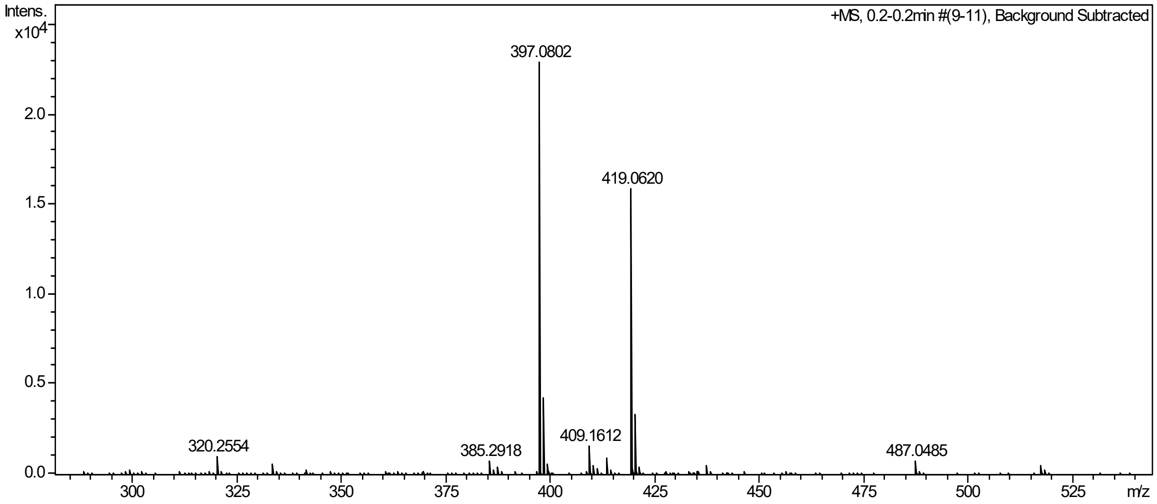

Figure S63. HRMS of 5m

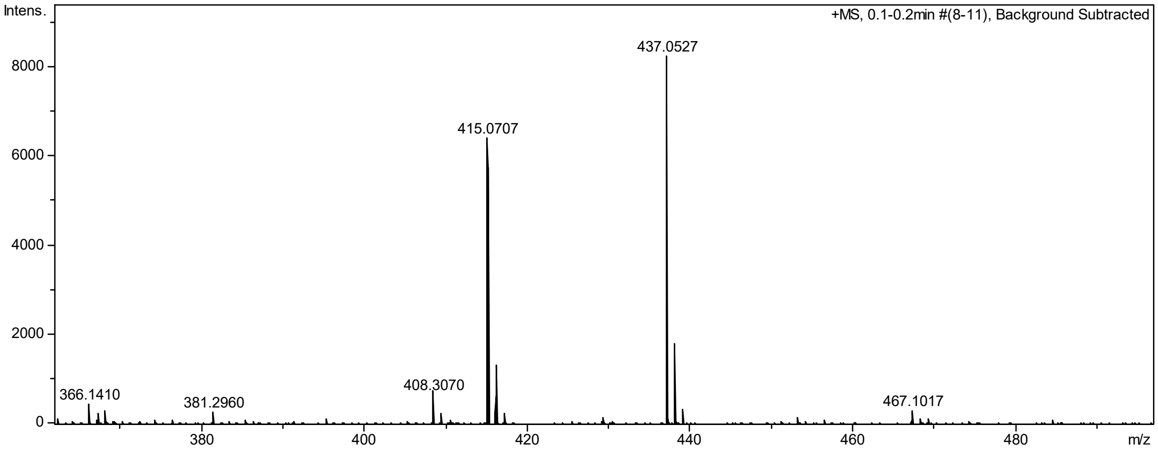

Figure S64. HRMS of 5n

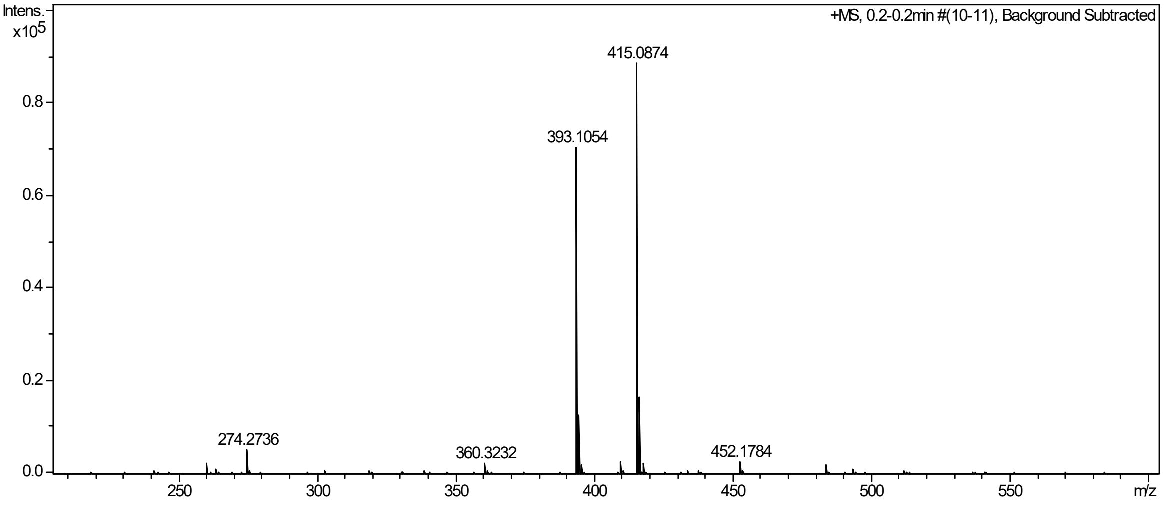

Figure S65. HRMS of 5o

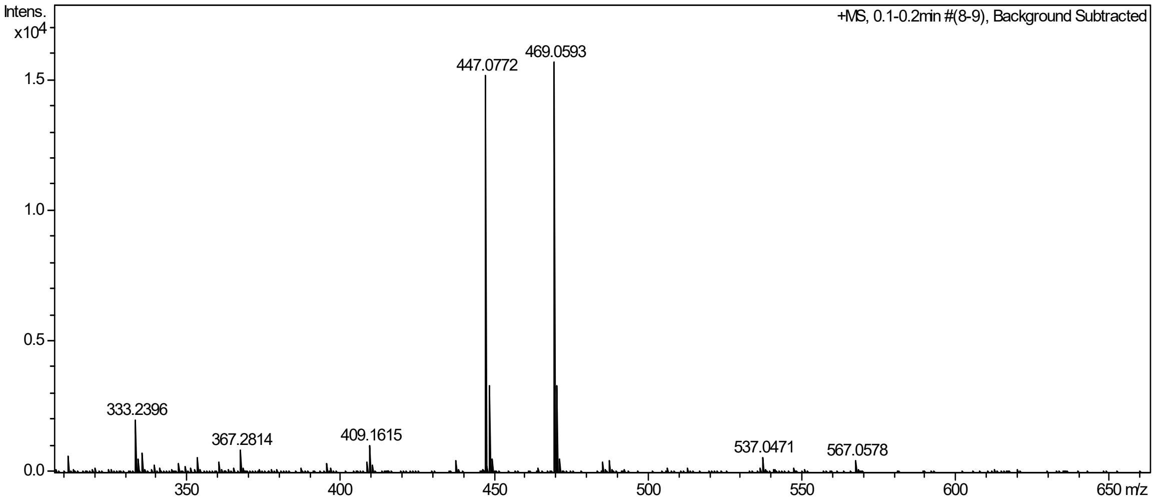

Figure S66 HRMS of 5p

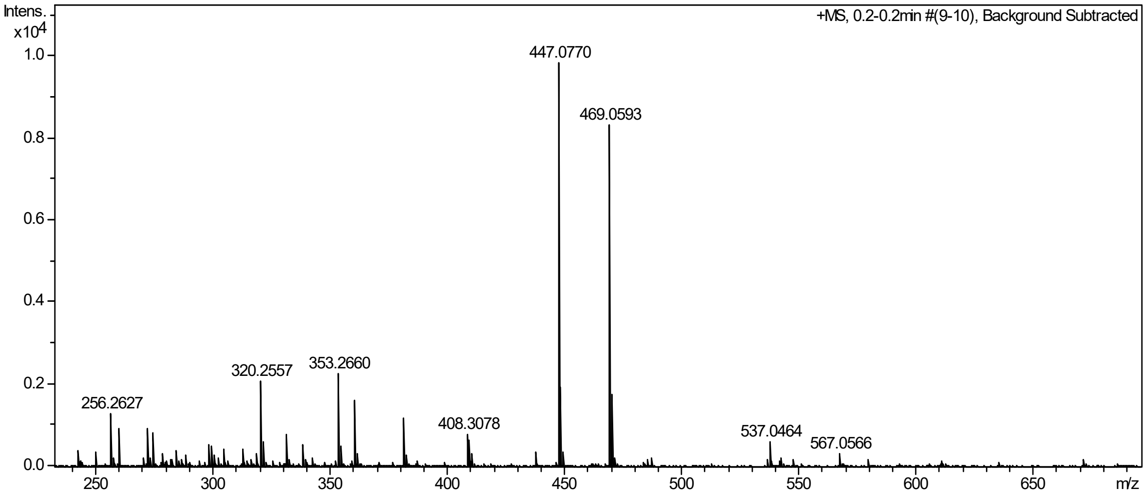

Figure S67. HRMS of 5q

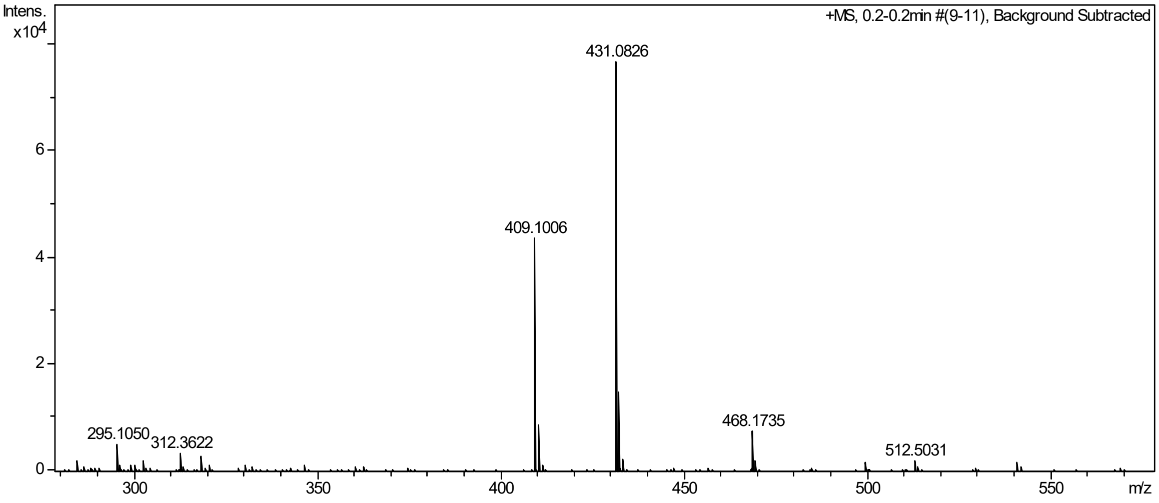

Figure S68. HRMS of 5r

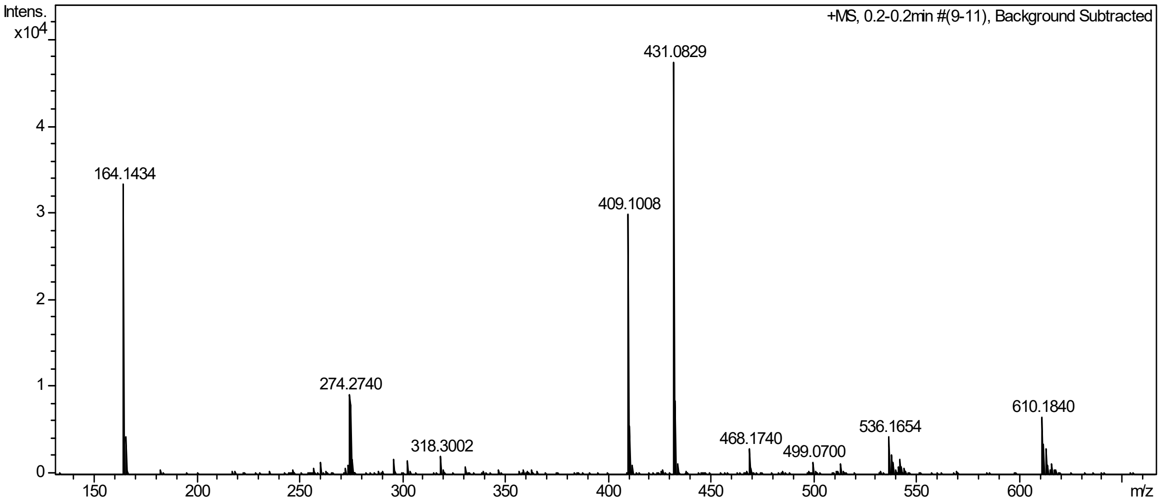

Figure S69. HRMS of 5s

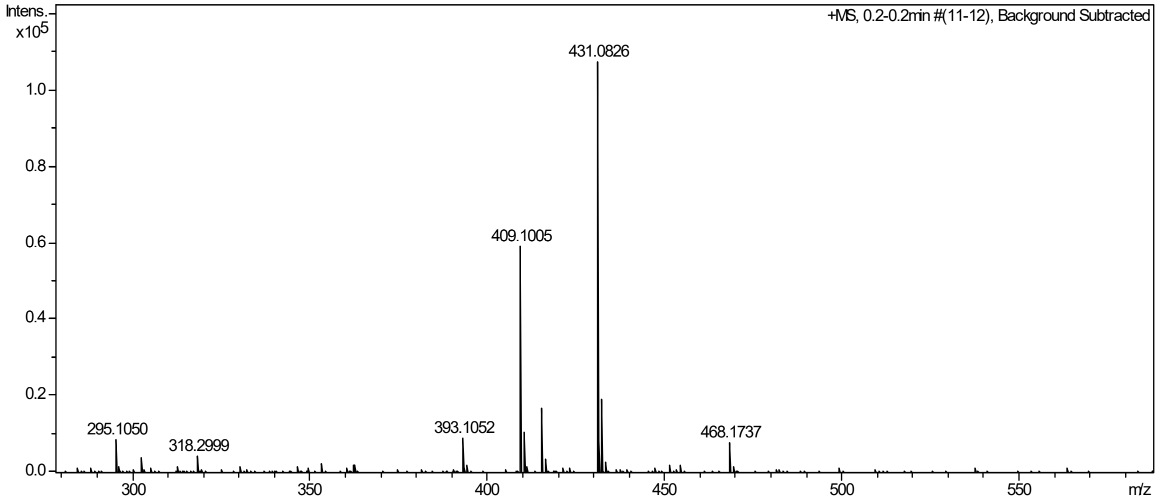

Figure S70. HRMS of 5t

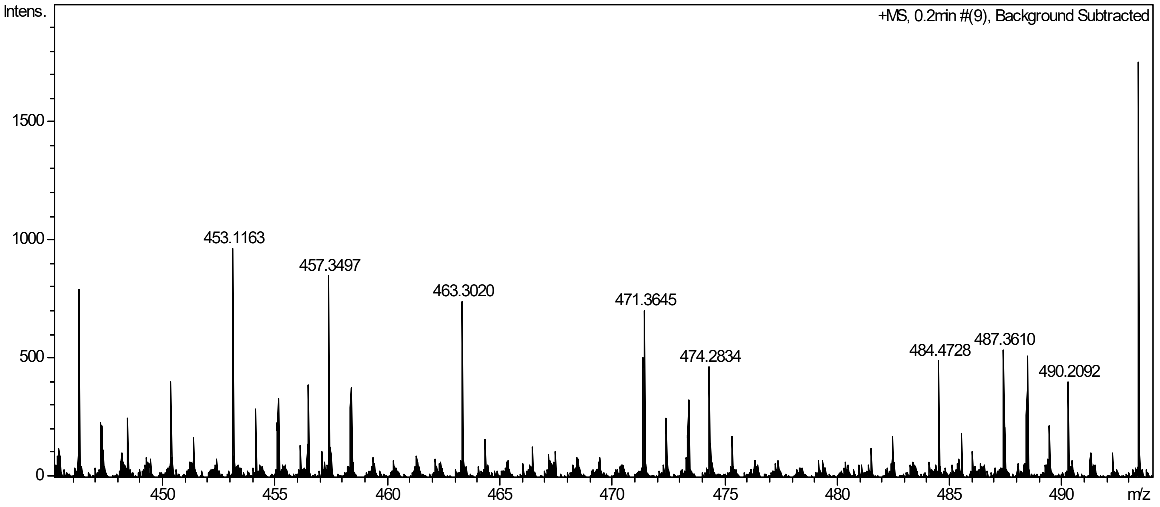

Figure S71. HRMS of 5u
